# Dynamic Optical Lattices Through Conducting Polymer‐Gated Confinement

**DOI:** 10.1002/adma.202520674

**Published:** 2025-12-22

**Authors:** Dongqing Lin, Yulong Duan, Suraya Kazi, Magnus P. Jonsson

**Affiliations:** ^1^ Laboratory of Organic Electronics, Department of Science and Technology (ITN) Linköping University Norrköping Sweden

**Keywords:** conducting polymers, Mie collective lattice resonances, redox switching, tunable light confinement

## Abstract

Organic metasurfaces based on conducting polymers are emerging as new opportunities for active nano‐optic devices, but their tunable plasmonic resonances are limited to infrared wavelengths (≥960 nm) and mainly with low quality‐factors (*Q* < 2). Here we propose the concept of a “conducting polymer gate” to endow organic metasurfaces with switchable high‐*Q* nonlocal resonances within the visible/NIR‐I regime (640–950 nm). Poly(3,4‐ethylenedioxythiophene) (PEDOT) is integrated into organic dielectric nanocylinder lattices and serves as a gate that controls optical leakage from the nanocylinders to the substrate. The quasi‐metallic PEDOT blocks such leakage channel and induces light confinement within the dielectric nanocylinders despite their low refractive index (*n*_die_ < 1.7). This facilitates the generation of Mie collective lattice resonances (CLRs) at the air superstrate with *Q*‐factor reaching 25, which is ∼16 times higher than previously reported organic metasurfaces near this spectral regime. Converting the PEDOT into its dielectric state opens the leakage channel and eliminates the CLRs, which can be reversibly switched by redox reactions. The concept of conducting polymer‐gated optical confinement in low‐index nanostructures provides new routes for intelligent nano‐optics including dynamic nonlocal metasurfaces.

## Graphical abstract

The conducting polymer gate is incorporated into organic dielectric lattices to control the dynamic switching of nonlocal Mie resonances in the visible/NIR regions. The “closed gate” from oxidized PEDOT confines the light within dielectric nanocylinders and activates nonlocal Mie resonances, while the “opened gate” from reduced PEDOT facilitates optical leakage from the dielectric nanocylinders to the substrate, which eliminates the Mie resonances.

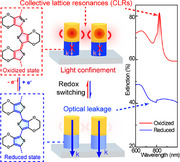

## 1 Introduction

Conducting polymers, with conjugated main‐chains ensuring π‐electron delocalization and endowing electric conductivity [1], have attracted widespread attention for organic electronic devices such as light‐emitting diodes [2], electrochromic displays [3] and solar cells [4]. Furthermore, tuning the redox states of conducting polymers enables modulation of their charge carrier density, which allows reversible transformation of their permittivity (or refractive index) between metallic and dielectric features. By taking advantages of this tunability, conducting polymers, including polyaniline (PANI) [5, 6, 7], poly(3,4‐ethylenedioxythiophene) (PEDOT) [8, 9] and poly(benzodifurandione) (PBFDO) [10], have been employed as active materials of nanoantenna units in optical metasurfaces, which has enabled dynamically switchable surface plasmon resonances [8, 11], tunable lensing [12], and beam steering [5, 6, 9]. However, conducting polymer plasmon resonances are limited to the infrared regime (≥960 nm) [8, 9, 13] and it would be highly desirable to achieve dynamic resonances within the visible/NIR‐I wavelength scope (400–950 nm). Of particular importance are high‐*Q* resonances, which can be provided by nonlocal metasurfaces where intense optical response is activated by far‐field coupling between neighboring nanoantenna units [14]. These are key to improving the performance of active nano‐optic devices, by providing high‐contrast switching [15], angular and spectral selectivity [16] and nonlinear optical properties [17]. Furthermore, dynamic high‐*Q* resonances located within 400–950 nm would enable metasurfaces suitable for integration with photonic circuits [18] or biosensors [19], giving prospects for a wider range of practical applications compared with systems limited to the infrared.

High‐*Q* nonlocal resonances in the visible/NIR‐I regimes have been realized by gold nanoantenna lattices [20], but are challenging to achieve through conducting polymer plasmonics. The reason is that the low charge carrier density of conducting polymers constrains the zero‐crossing point of the permittivity to low frequencies, which prohibits blueshifting plasmonic resonances into the visible regime. Recently, we demonstrated switchable nonlocal PEDOT metasurfaces [15] with plasmonic CLRs with *Q*‐factors improved to 12, but their resonance wavelengths (*λ*_r_ = 2000–4500 nm) were still restricted by the zero‐crossing point of the permittivity at around 900 nm. In the visible regime [21], conducting polymers have been explored as responsive surrounding medium [22] to tune the response of gold nanoparticles [6, 7, 23], although limited to local metasurfaces [7] with broadband optical response dominated by single nanoantenna units.

**FIGURE 1:**
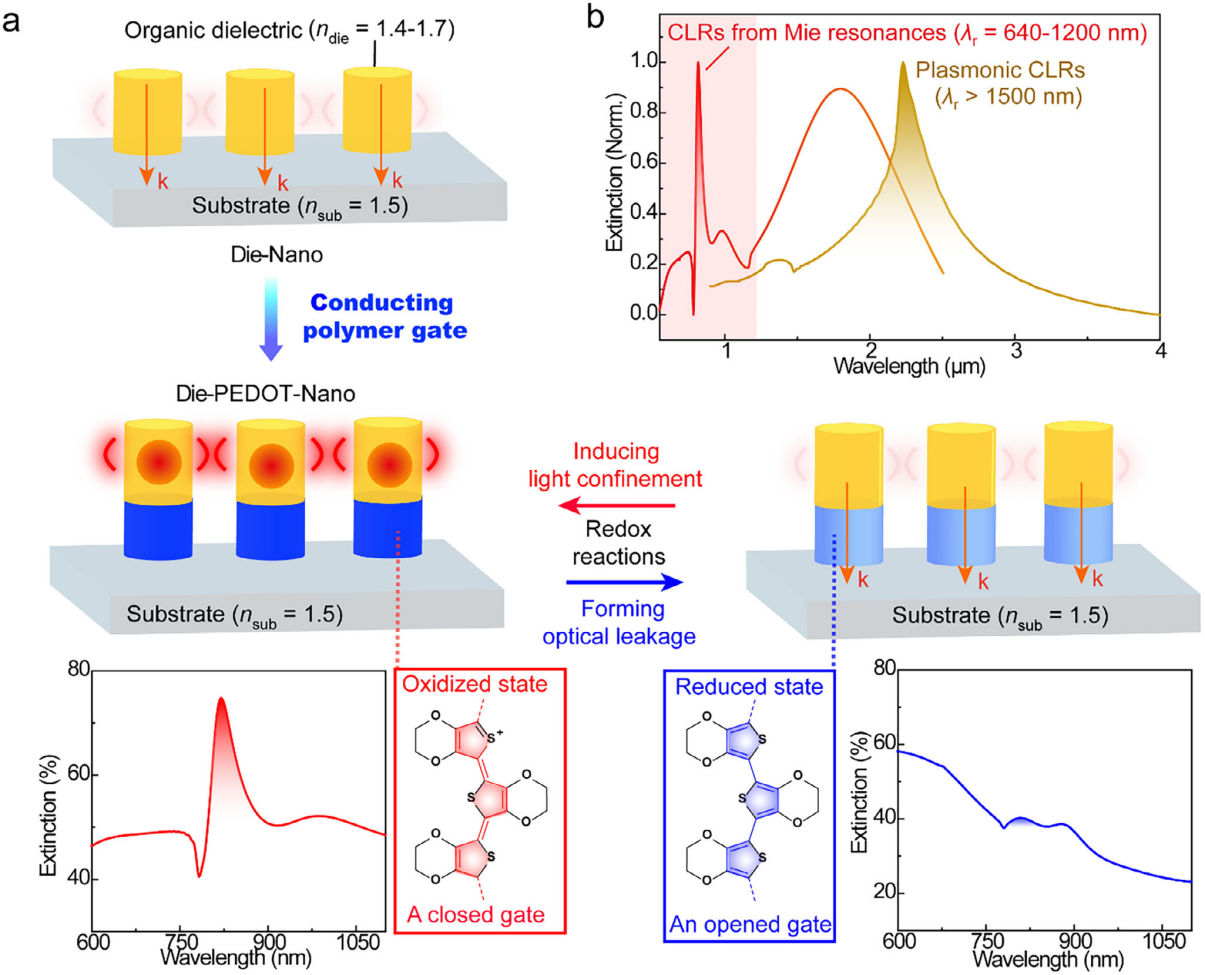
Concept of conducting polymer gate for the switching of nonlocal Mie resonances within visible/NIR‐I wavelengths. (a) Concept design of conducting polymer gates in Die‐PEDOT‐Nano arrays. The orange regions are organic dielectric nanocylinders (with refractive index *n*_die_ = 1.4–1.7). The PEDOT nanocylinders in blue serves as gates to control light confinement (indicated by red circles) within the dielectric nanocylinders, where the oxidized state of PEDOT (in deep blue, as a closed gate) activates light confinement to induce Mie resonances while the reduced state (in light blue, as an opened gate) forms an optical leaky channel which eliminates the Mie resonance. The gating effect is exemplified through simulated spectra for a Die‐PEDOT‐Nano array when PEDOT in oxidized (left) and reduced (right) states. (b) Simulated example extinction spectra highlighting the wavelength difference of collective lattice resonances (CLRs) between from Mie resonances in this work and plasmonic resonances in previous work [15]. The Mie CLRs originate from a Die‐PEDOT‐Nano array, while the plasmonic CLRs stem from a PEDOT nanoantenna array.

In this work, we present a new concept where a conducting polymer is used neither as plasmonic material nor as surrounding medium but as an optical gate. The principle utilizes that metallic surfaces can activate waveguide‐like modes and induce optical confinement in low‐index dielectric nanostructures [24]. This allows the creation of a “conducting polymer gate” which can reversibly induce and eliminate light confinement in low‐index organic nanostructures, leading to switchable high‐*Q* Mie CLRs. As illustrated in Figure 1a, we designed Die‐PEDOT‐Nano lattices consisting of nanocylinders made from PEDOT and an organic dielectric, where the PEDOT separates the dielectric part from the substrate (glass). The organic dielectric has too low refractive index (*n*_die_ < 1.7) to support optical confinement if directly placed on the substrate (Die‐Nano arrays in Figure 1a). However, the quasi‐metallic PEDOT (oxidised state) can serve as “a closed gate” that blocks optical leakage from the dielectric nanocylinders to the substrate, which facilitates confinement of an electric dipole mode within the dielectric nanocylinders. This enables high‐*Q* nonlocal Mie CLRs (*Q* up to 25) at the air superstrate (Figure 1b), with wavelengths tunable in the visible/NIR regimes from around 640 to 1200 nm. Hence, the PEDOT layer can act as efficient gate also at wavelengths where it cannot provide plasmonic resonances, essentially filling the wavelength gap of organic plasmonic CLRs [15]. When converted to its reduced state, the PEDOT layer instead works as “an open gate” by generating optical leakage between the low‐index dielectric nanocylinders and the substrate, eliminating optical confinement and the nonlocal Mie resonances (Figure 1a). Opening and closing the conducting polymer gate by redox reactions thereby enables complete and reversible suppression of sharp resonance peaks, providing new opportunities for dynamically tunable nonlocal metasurfaces in the visible/NIR‐I regime.

## 2 Predicting Mie CLRs Assisted by a Closed Conducting Polymer Gate

**FIGURE 2:**
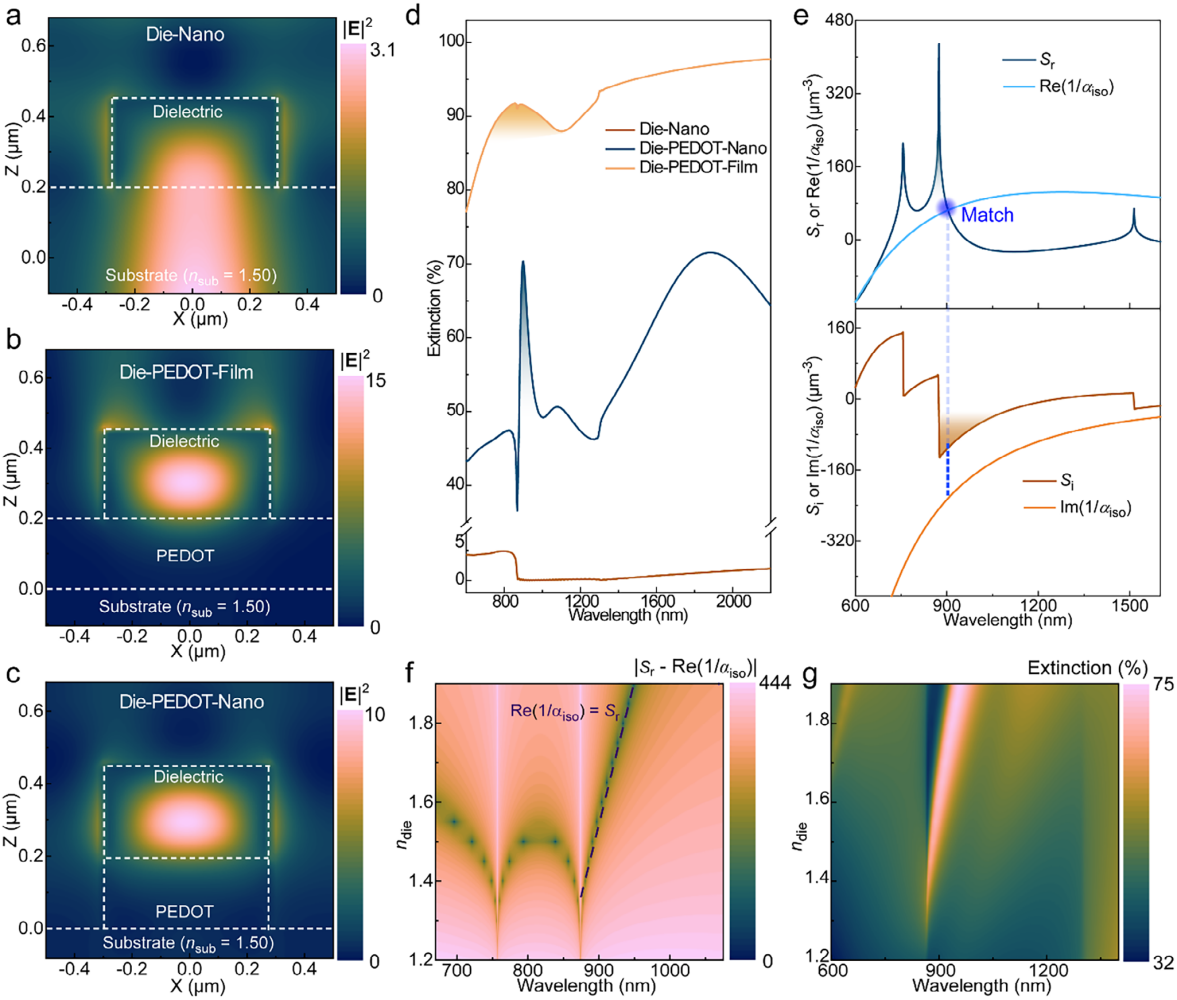
Dipolar calculations and FDTD simulations for Mie CLRs from hexagonal lattices consisting of PEDOT and dielectric nanoantennas. (a–c) Transverse‐magnetic (TM) near‐field distributions (at 870–900 nm) of hexagonal Die‐Nano, Die‐PEDOT‐Film and Die‐PEDOT‐Nano arrays. (d) Simulated extinction spectra of the above three array models. The extinction peak with a narrow region (around 850–910 nm) is assigned to the Mie resonance. (e) *S*‐(1/*α*_iso_) analysis of a Die‐PEDOT‐Nano array model. Both *S* and 1/*α*_iso_ are complex parameters (*S* = *S*_r_ + *iS*_i_ and 1/*α*_iso_ = Re[1/*α*_iso_] + *i*Im[1/*α*_iso_]). The dashed blue line marks the wavelength that fulfills the CLR‐matching condition of Re(1/=*α*_iso_)=*S_r_*. (f) *n*_die_‐dependent |*S*_r_—Re(1/*α*_iso_)| values of Die‐PEDOT‐Nano array. *n*_die_ is the refractive index of the dielectric. (g) *n*_die_‐dependent extinction spectra of Die‐PEDOT‐Nano array. In all panels, the dielectric nanocylinders have a diameter *d* = 0.56 µm and a height *h* = 0.25 µm. The height of the PEDOT nanoantennas and film are 0.2 µm.

As a key precondition for nonlocal Mie resonances, we first tested whether optical fields can be confined within dielectric nanocylinders if adding a quasi‐metallic PEDOT layer as the closed state of a conducting polymer gate. The dielectric nanocylinders (diameter *d* = 0.56 µm, height *h* = 0.25 µm) with refractive index *n*_die_ = 1.6 were arranged in a hexagonal array with periodicity *r* = 1.0 µm. If these dielectric nanocylinders are directly located on the substrate (Die‐Nano arrays), finite‐difference time‐domain (FDTD) simulations reveal that the electrical field at wavelengths near *n*_die_ *d* ≈ 870–900 nm (Figure 2a) is leaked from the nanocylinders to the substrate (*n*_sub_ ≈ 1.5). This is expected because of the small refractive index contrast [25] and the large surface overlap between the dielectric nanocylinders and the substrate [26]. The introduction of a non‐patterned quasi‐metallic PEDOT film (*h* = 0.2 µm, an electric conductivity around 1000 S cm^−1^) between the dielectric nanocylinders and the substrate (Die‐PEDOT‐Film, Figure 2b) suppresses such optical leakage and leads to 4 times enhancement of the electrical field intensity (|**E**|^2^) within the dielectric nanocylinders compared to Die‐Nano arrays (without PEDOT). Meanwhile, the magnetic field is circularly distributed around the nanocylinder edges (Figure S2). Both features can be assigned to an electric dipole mode in the dielectric nanocylinders [27]. In addition, we find no clear confinement of the magnetic field within the dielectric nanocylinders and the distribution of the electric field around the outside nanocylinder edges, which indicates that there is no magnetic dipole resonance. Efficient light confinement only with electric dipolar resonance was also observed when patterning also the PEDOT to obtain Die‐PEDOT‐Nano arrays (Figure 2c). These results confirm our proposed concept that quasi‐metallic PEDOT can work as a closed gate that blocks optical leakage and facilitate efficient light confinement within low‐index dielectric nanocylinders.

Figure 2d presents simulated extinction spectra of Die‐Nano, Die‐PEDOT‐Film, and Die‐PEDOT‐Nano arrays (all with *r* = 1.0 µm). The Die‐Nano array only has an extremely weak peak around *λ*_r_ ≈ 850 nm, consistent with the high optical loss from the leakage channel. Adding a PEDOT film (in Die‐PEDOT‐Film array) eliminates such optical leakage and induces a broad resonance (full width at half maximum FWHM = 400‐500 nm) at *λ*_r_ ≈ 860 nm. Patterning the PEDOT to obtain Die‐PEDOT‐Nano array gives similar light confinement but a much sharper resonance at *λ*_r_ ≈ 900 nm, with *Q*‐factor increasing from < 2 to 21 (FWHM = 42 nm). Such high‐*Q* resonance is distinguished from the localized Mie resonance of an isolated Die‐PEDOT‐Nano unit (Figure S5) and suggests nonlocality of the resonance mode. The stark difference from the Die‐PEDOT‐Film array implies that efficient nonlocality is only supported if the PEDOT is patterned and integrated into the nanocylinders, which lowers the PEDOT coverage and suppresses loss related to its high extinction coefficient. The broader peak at around 1850 nm for the Die‐PEDOT‐Nano array, which is not present for the Die‐PEDOT‐Film array, is due to a localized surface plasmon resonance in the PEDOT nanodisks [8, 15].

To verify nonlocality of the Die‐PEDOT‐Nano array resonance, we analyzed the CLR‐matching conditions of the system. Because the nanoantenna units only showed electric dipoles, the periodic polarizability of the full array *α*_p_ = (1/*α*_iso_—*S*)^−1^ was calculated based on electrical dipole components [27]. Here, 1/*α*_iso_ (complex type: 1/*α*_iso_ = Re[1/*α*_iso_] + *i*Im[1/*α*_iso_]) is the isolated reverse polarizability of a single dielectric nanocylinder and *S* (complex type: *S* = *S*_r_ + *iS*_i_) is the electric dipole sum (*S*) in a lattice (see Materials and Methods). To induce efficient nonlocal coupling and CLR at *λ*_r_, *α*_p_ should fulfill Re[1/*α*_iso_] = *S*_r_ (*S*_r_ > 0) and exhibit a small value of |Im(1/*α*_iso_)—*S*_i_|. We calculated *S* for the air superstrate because the observed *λ*_r_ is close to the wavelength position of CLR $(\sqrt[{3}]{2/3}r$) for a hexagonal lattice with air as medium, while CLR at the substrate [15] would occur at longer wavelengths ($\sqrt[{3}]{2/3}n_{\mathrm{sub}}r$) far from *λ*_r_. Figure 2e marks the matching position at Re(1/*α*_iso_) = *S*_r_, and it occurs at 904 nm that is very close to *λ*_r_ ≈ 900 nm in the simulated extinction spectrum (Figure 2d). The large *S*_r_ suggests efficient electric dipolar coupling between adjacent nanocylinder units. Meanwhile, the large magnitude of *S*_i_ significantly reduces damping relaxations of the dielectric nanocylinders. These results demonstrate that the Mie resonance reaches CLR‐matching conditions at *λ*_r_, verifying the clear nonlocal behavior. Despite PEDOT having similar real refractive index to air, |*S*_r_| and |*S*_i_| sharply decrease to ∼0 if calculating *S* using PEDOT as medium instead of air (Figure S6). This is due to the large extinction coefficient of PEDOT, which hinders efficient nonlocal coupling.

We further demonstrate the conceptual generality of conducting polymer gating by changing refractive index of the dielectric layer (*n*_die_) for the same dimensions of hexagonal Die‐PEDOT‐Nano arrays. Figure 2f shows that dielectric nanocylinders down to *n*_die_ as low as 1.35 can fulfill Re(1/*α*_iso_) = *S*_r_ for CLR‐matching conditions. Indeed, simulated extinction spectra evidence narrow and intense resonance peaks (*λ*_r_ ≈ 870–930 nm) for *n*_die_ ≥ 1.35 (Figure 2g). Furthermore, this concept works also for other conducting polymers such as poly(3,4‐ethylenedioxythiophene:sulfate) (PEDOT:Sulf) (Figure S11, with a conductivity of 5200 S cm^−1^) and PBFDO (Figure S14, with an average conductivity of ∼900 S cm^−1^), for which we also observed efficient electric dipolar resonances within the dielectric nanocylinders (if *n*_die_ = 1.6, |**E**|^2^ [= 9‐15), and sharp resonances at *λ*_r_ ≈ 890–900 nm for *n*_die_ ≥ 1.40. The above results not only confirm the excellent generality of the concept, but also that the conducting polymer gate can lower the *n*_die_ requirement for nonlocal Mie resonances from 2–4 [27, 28] to 1.4, which can be easily available for a broad range of dielectric materials including organic polymers (usually *n*_die_ = 1.4–1.8) [29].

## 3 Experimental Demonstrations of High‐*Q* Organic CLRs

**FIGURE 3:**
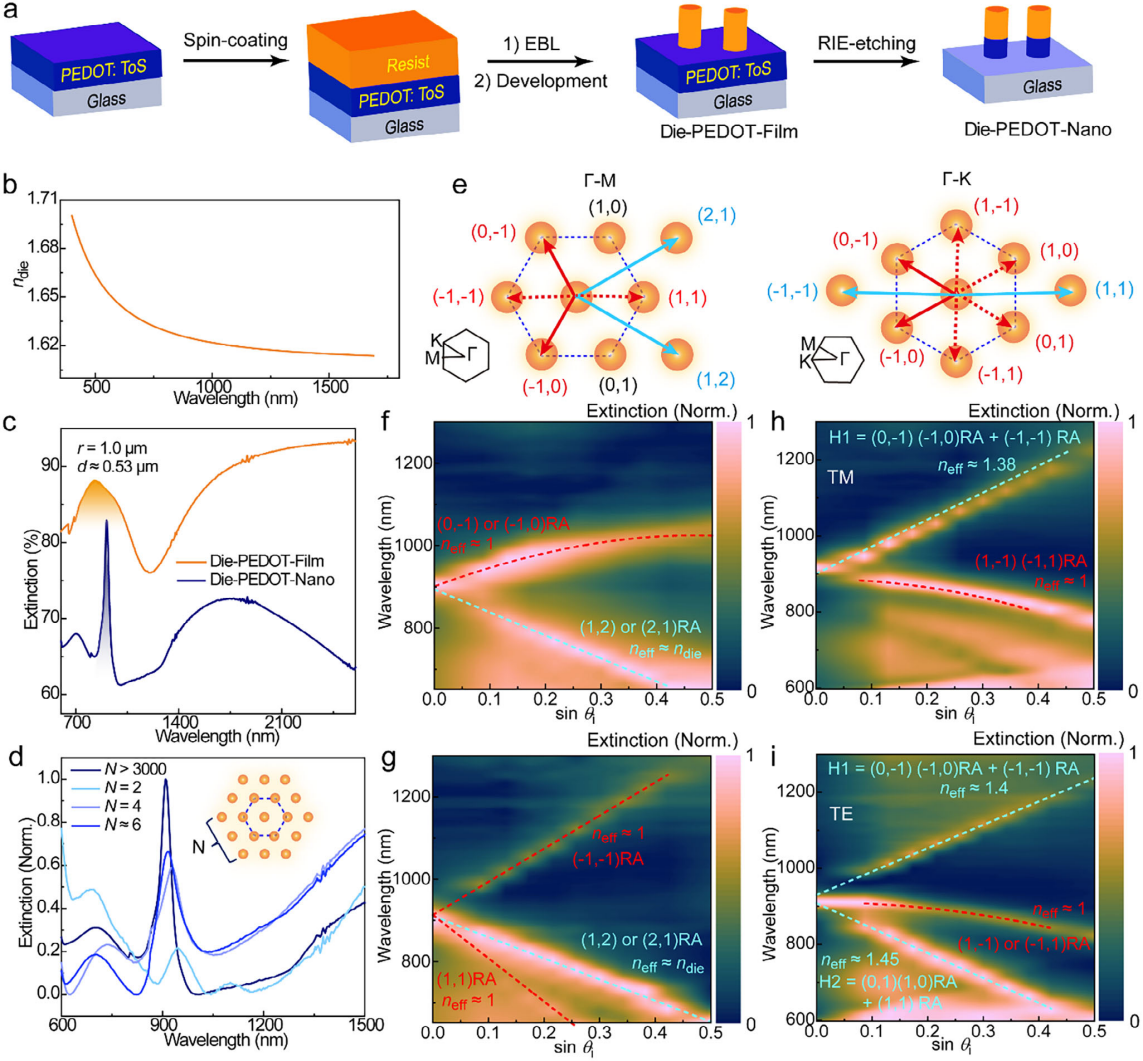
Preparation and Extinction spectra of Die‐PEDOT‐Nano arrays (*r* = 1.0 µm and *d* = 0.52 µm). (a) Schematic process flow of preparing nonlocal Die‐PEDOT‐Nano metasurfaces, including spin‐coating electron‐beam negative resist, electron‐beam lithography (EBL), development, and dry‐etching with oxygen plasma. (b) *n*_die_ of ma‐N 2405 after electron beam exposure, measured by spectroscopic ellipsometry. (c) Extinction spectra of Die‐PEDOT‐Nano and Die‐PEDOT‐Film arrays. The extinction peak with a shallow region (at around 850–910 nm) was assigned to the Mie resonance. (d) Dependence on the extinction spectra of the layer number (*N*) for arrays. (e) Rayleigh Anomaly (RA) analysis in hexagonal reciprocal lattice diagrams along the Γ−M and Γ−K directions, respectively. (f–i) Angle‐dependent extinction spectra (experimental results) of the same Die‐PEDOT‐Nano array in Γ−M_TM_, Γ−M_TE_, Γ−K_TM_ and Γ−K_TE_ modes, respectively. The assignments of RAs were marked corresponding to the arrows in (e).

To experimentally test the concept of conducting polymer‐gated Mie CLRs, we fabricated a hexagonal Die‐PEDOT‐Nano array (*d* = 0.535 µm and *r* = 1.0) through electron beam lithography on a glass slide (Figure 3a) and characterized the lattice by scanning electron microscopy (SEM, Figure S15). Here, the negative resist ma‐N 2405 [30] serves as the organic dielectric material, with refractive index *n*_die_ = 1.61–1.64 (Figure 3b) which is within the theoretically predicted range to allow for CLRs (Figure 2g). Figure 3c presents extinction results and show that the Die‐PEDOT‐Nano array produces a sharp Mie resonance at *λ*_r_ ≈ 910 nm. The resonance linewidth is 37 nm which gives a high *Q*‐factor around 25, similar to the simulation results. As shown in Figure 3d, the resonance intensity gradually decreased if decreasing the number of neighboring nanocylinders (layer number, *N*) of the hexagonal array, and the resonance completely vanished if the nanocylinders were disordered and positioned in different orientations on the substrate (Figure S20). These results experimentally confirm that the high‐*Q* Mie resonance originates from the nonlocal dipolar coupling in the lattice. For the corresponding Die‐PEDOT‐Film array, we only observed a broad peak (*λ*_r_ ≈ 850 nm and *Q* = 2.4, Figure 3c), agreeing with the theoretical findings that it is not sufficient to use PEDOT as substrate but that the Mie CLR is only supported if integrating the PEDOT into the nanocylinders.

Angle‐dependent extinction measurements provide deeper insight into the angular response of the organic high‐*Q* CLRs. Figure 3e illustrates the diffraction orders of Rayleigh Anomalies (RA) along the Γ–M and Γ–K directions [31], where each mathematical relationship among *λ*_r_, *r*, effective refractive index (*n*_eff_) and incident angle (*θ*_i_) was deduced in Equations S1–S50. Along the Γ‐M direction, the sharp resonance at *λ*_r_ ≈ 910 nm possesses an angular response following an equation λ_r_  ≈  *r* sin (*θ*_i_+  π/3), which can be assigned to the first diffraction order of (0,−1) (−1,0) RAs under TM polarized excitation (Figure 3f). Under transverse‐electrical (TE) polarization (Figure 3g), two angular responses with equations $\lambda_{r}\approx \frac{\sqrt{3}}{2}r\;(n_{\mathrm{eff}}-\sin\;\theta_{i}$) and $\lambda_{r}\approx \frac{\sqrt{3}}{2}r\;(n_{\mathrm{eff}}+\sin\;\theta_{i}$) are also present for the sharp resonance, belonging to the first diffraction order of (1,1) and (−1,−1) RAs, respectively. These three kinds of RAs stem from the dipole radiation [32]. Furthermore, *n*_eff_ extracted from these RAs approaches 1, confirming that the nonlocal dipolar coupling exists at the air superstrate rather than along the glass substrate. The above findings agree with the calculation of *S* that predicts the presence of efficient dipolar coupling (*S*_r_ = 62 um^−3^) and the suppression of damping relaxations (*S*_i_ ≈ −110 um^−3^) in the air medium.

We also observed another angular response approximately following $\lambda_{r}\approx \frac{1}{2}n_{\mathrm{eff}}r-\frac{\sqrt{3}}{4}r\sin\;\theta_{i}$ at *λ*_r_ = 910 nm, which can be assigned to the second diffraction order of (2,1) (1,2) RAs (Figure 3f,g). These second‐order RAs are not due to the PEDOT only but exist also if removing the substrate (Figure S26). Interestingly, *n*_eff_ extracted for these (2,1) (1,2) RAs is close to *n*_die_, revealing that there is another nonlocal coupling interaction between the dielectric nanocylinders. Through the analysis of CLR‐matching conditions using the dielectric nanocylinders as the *S* medium (Figure S27), this high‐order coupling cannot reduce |Im(1/*α*_iso_)—*S*_i_| for stronger electric dipole polarizability, ruling out the contribution from dipole radiation. Considering the coupling direction being approximate to the in‐plane component of the incident wave vector (Figure S24b), the coupling can instead be attributed to the diffraction grating effect for the dielectric nanocylinder array. We note that such second‐order RAs do not exist at resonance wavelengths in plasmonic hexagonal CLR arrays [31], but could be observed and confirmed for Mie CLRs of hexagonal Die‐Nano arrays (without PEDOT) if increasing *n*_die_ ≥ 2.2 to allow for light confinement (Figures S28–S30). Hence, even with the low *n*_die_ ≈ 1.6 of the organic nanocylinders, the light confinement induced by the conducting polymer gate can also facilitate second‐order RAs similar to those of Die‐Nano array that requires higher *n*_die_ > 2.

The angle‐dependent spectra further imply that the hexagonal Die‐PEDOT‐Nano array may also support second‐order RAs mixed with first‐order RAs along the Γ–K direction. In Figure 3h,i, the angular response marked H1 followed a linear equation λ_r_  ≈  0.64*r*(*n*_eff_ +  sin *θ*_i_), which is different from both $\lambda_{r}\approx \;0.75r(\frac{2}{\sqrt{3}}n_{\mathrm{eff}}+\sin\;\theta_{i})$ related to the first order of (0,−1) (−1,0) RAs and also different from λ_r_  ≈  0.5*r*(*n*_eff_  +  sin *θ*_i_) of the second order (−1,−1) RAs. Furthermore, *n*_eff_ for this H1 mode was evaluated to 1.40, which is roughly the average between that of air (*n*_air_ = 1) and the dielectric nanocylinders that serve as the media for the first and second‐order RAs, respectively. The results thereby suggest that the H1 mode corresponds to a mixed coupling mode based on the above two kinds of RAs. Similarly, the angular response marked H2 fitted with an equation λ_r_  ≈  0.61*r*(*n*_eff_ − sin *θ*_i_) and an intermediate *n*_eff_ = 1.38 also has a mixed feature, here between (0,1) (1,0) RAs ($\lambda_{r}\approx \frac{\sqrt{3}}{2}n_{\mathrm{eff}}r-\frac{3}{4}r\sin\;\theta_{i}$) at the air superstrate and (1,1) RA ($\lambda_{r}\approx \;\frac{r}{2}\left[n_{\mathrm{eff}}-\sin\;\theta_{i}\right]$) in the dielectric nanocylinders. Such mixed coupling modes could be partly observed also in Die‐Nano arrays (without PEDOT) if increasing *n*_die_ ≥ 2.2 (Figures S28, S29). By contrast, plasmonic CLRs in hexagonal arrays [31] only possess pure first orders of (0,−1) (−1,0) RAs or (0,1) (1,0) RAs rather than the mixed modes. These mixed H1 and H2 coupling modes can thereby be seen as characteristic of Mie CLRs and related to the effect of light confinement in dielectric nanocylinders. Hence, the light confinement assisted by conducting polymer gate also works for the activation of mixed nonlocal coupling for high‐*Q* Mie resonances.

## 4 Achieving Visible/NIR CLRs Through Tuning Lattice Periodicity

**FIGURE 4:**
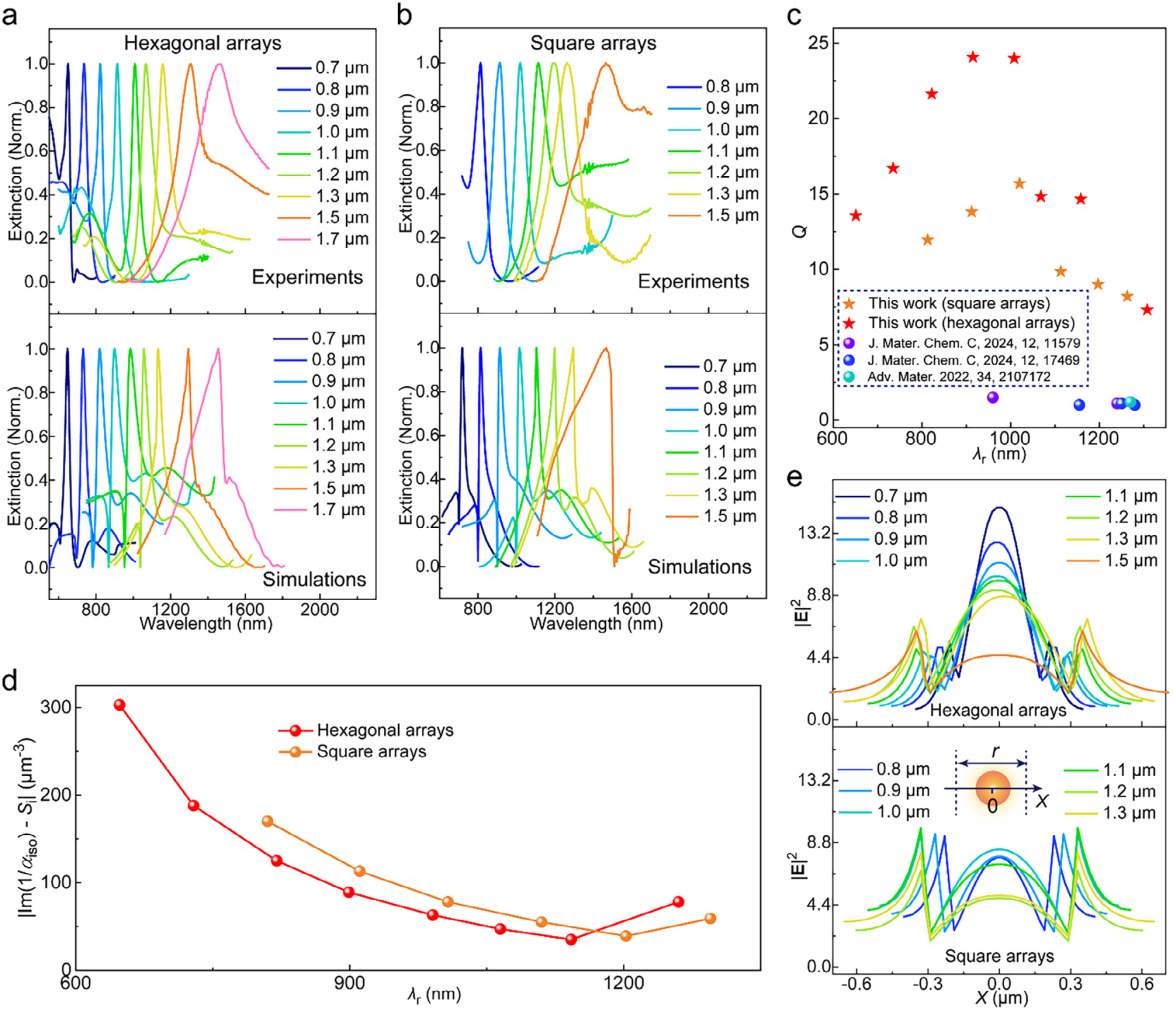
Extinction spectra and *Q*‐factor analysis for Die‐PEDOT‐Nano arrays. (a) Periodicity‐dependent extinction spectra of hexagonal arrays from experimental results and FDTD simulations. The nanoantenna diameters were set as *d* = 0.36 µm (*r* = 0.7 µm), 0.39 µm (*r* = 0.8 µm), 0.48 µm (*r* = 0.9 µm), 0.535 µm (*r* = 1.0 µm) and 0.6 µm (*r* = 1.1∼1.7 µm), respectively. (b) Periodicity‐dependent extinction spectra of square arrays from experimental results and FDTD simulations. The nanoantenna diameters were set as *d* = 0.39 µm (*r* = 0.7 µm), 0.41 µm (*r* = 0.8 µm), 0.50 µm (*r* = 0.9 µm), and 0.62 µm (*r* = 1.0∼1.7 µm), respectively. (c) Experimentally evaluated *Q*‐factors according to this work and previous works on organic nanoantennas with resonance peaks at *λ*_r_ < 1300 nm. (d) Damping relaxation part |*S*_i_—Im(1/*α*_iso_)| of CLR under various *λ*_r_, respectively. (e) The distribution of electrical field intensity (|**E**|^2^) within a periodic size. The line detector passes through the dielectric nanoantenna (at the fixed height *z* ≈ 0.3 µm). These |**E**|^2^‐distributions of hexagonal and square arrays are based on the TM mode.

To evaluate the achievable wavelength range for high‐*Q* CLRs with Die‐PEDOT‐Nano arrays, we varied the periodicity *r* and diameter *d* (structural characterizations in Figures S31–S33) to maintain matching conditions. Experimental and simulated extinction spectra (Figure 4a) demonstrate that *λ*_r_ can be controlled from 640 to 1500 nm upon varying *r* from 0.7 to 1.7 µm. The Mie resonance peaks at *λ*_r_ = 630∼1160 nm (*r* = 0.7∼1.3 µm) obey the *S*‐1/*α*_iso_ relationships of CLR‐matching conditions (Figure S34) and follow the angular response covering first and second orders of RAs (Figures S35–S40), both of which firmly identify the nonlocality. Moreover, these resonances have large extinction depths (20∼30%, Figure S43) and narrow resonance linewidths ranging from 38 to 80 nm. Apart from hexagonal arrays, square lattices with *r* = 0.7∼1.3 µm also achieve narrow Mie resonances with *λ*_r_ = 700–1300 nm (Figure 4b), where the nonlocality is also confirmed by *S*‐1/*α*_iso_ analysis (Figure S46) and angle‐dependent extinction spectra (Figures S47–S53). Importantly, our results show that organic short‐wavelength CLRs can be achieved in the visible/NIR‐I spectral regions (*λ*_r_ < 950 nm), which cannot be covered through surface plasmon resonances of PEDOT nanoantennas (Figure S55) due to the limited plasma frequency [13].

Figure 4c presents experimentally obtained *Q*‐factors of these organic CLRs at different *λ*_r_ (or *r*). According to the nonlocal coupling modes in Die‐PEDOT‐Nano arrays, we deduced that the *Q*‐factors can be influenced by coupling interactions from both first and second‐order RAs. Because the relevant first‐order RAs originate from electric dipole radiation at the air superstrate, they affect *Q*‐factors mainly by dipolar radiative loss from damping relaxation, which is quantified by |*S*_i_—Im(1/*α*_iso_)| at *λ*_r_ [15, 20]. Along with increasing *r* of hexagonal arrays, |*S*_i_—Im(1/*α*_iso_)| decreases from 300 to < 50 µm^−3^ (Figure 4d), which enables better suppression of dipolar radiation loss. These results lead to increasing *Q*‐factors, from 13 (*r* = 0.7–0.8 µm, *λ*_r_ = 650–750 nm) to 25 (*r* = 1.0 µm, *λ*_r_ ≈ 910 nm). The above changes for |*S*_i_—Im(1/*α*_iso_)| and *Q*‐factors are also confirmed for square arrays (with *r* = 0.8–1.0 µm). However, our results show that the *Q*‐factors decrease if further increasing *r* to 1.1–1.3 µm, which is contradictory to decreasing |*S*_i_—Im(1/*α*_iso_)|. This suggests that the *Q*‐factors, especially for Die‐PEDOT‐Nano arrays with larger periodicity (*r* > 1.0 µm), could be constrained by other factors which are related to the coupling interactions from second‐order RAs.

Considering the relationship of second‐order RAs with the electric dipole resonance in the dielectric nanocylinders, we investigated whether its resonance intensity could influence the *Q*‐factors. Usually, the stronger electric dipole resonance in dielectric nanocylinders suggests the more efficient storage of electromagnetic energy [33], which enhances light‐matter interactions and improves *Q*‐factors [34]. Figure 4e illustrates the dependence of *r* on the electrical field distributions at *λ*_r_, where a Die‐PEDOT‐Nano unit is located at *X* = 0. Increasing *r* of the hexagonal arrays continuously lowers |**E**|^2^ [2 within the dielectric nanocylinders, from 15 (*r* = 0.7 µm) to 4.4 (*r* = 1.5 µm). This change is accompanied with reduced extracted *n*_eff_ of the mixed H1 or H2 modes along the Γ–K directions if *r* ≥ 1.1 µm (Figures S38–S42). These results indicate that the weaker electric dipole resonance in dielectric nanocylinders is linked to impairing the coupling interactions from the second‐order diffraction grating effect, which reduces light‐matter interactions and lowers *Q*‐factors of CLRs for large *r* (Figure 4c). Similarly, weaker |**E**|^2^ within the nanocylinders (Figure 4e) and lower *n*_eff_ of mixed coupling modes (marked HS in Figures S47–S52) in square Die‐PEDOT‐Nano arrays agree with lower *Q*‐factors than those from hexagonal arrays for the same *r*. Combining the above analysis, *r* = 1.0–1.1 µm in hexagonal arrays forms an optimum that achieves efficient coupling interactions simultaneously from both first‐order and second‐order RAs, leading to the maximum *Q*‐factor up to 25. Such high *Q*‐factor can be 16–20 times higher than surface plasmon resonances (*Q* = 1–1.5) of conducting polymer nanoantennas (*λ*_r_ < 1300 nm) in previous works [10, 11, 13].

## 5 Opening and Closing the Conducting Polymer Gate for Switchable CLRs

**FIGURE 5:**
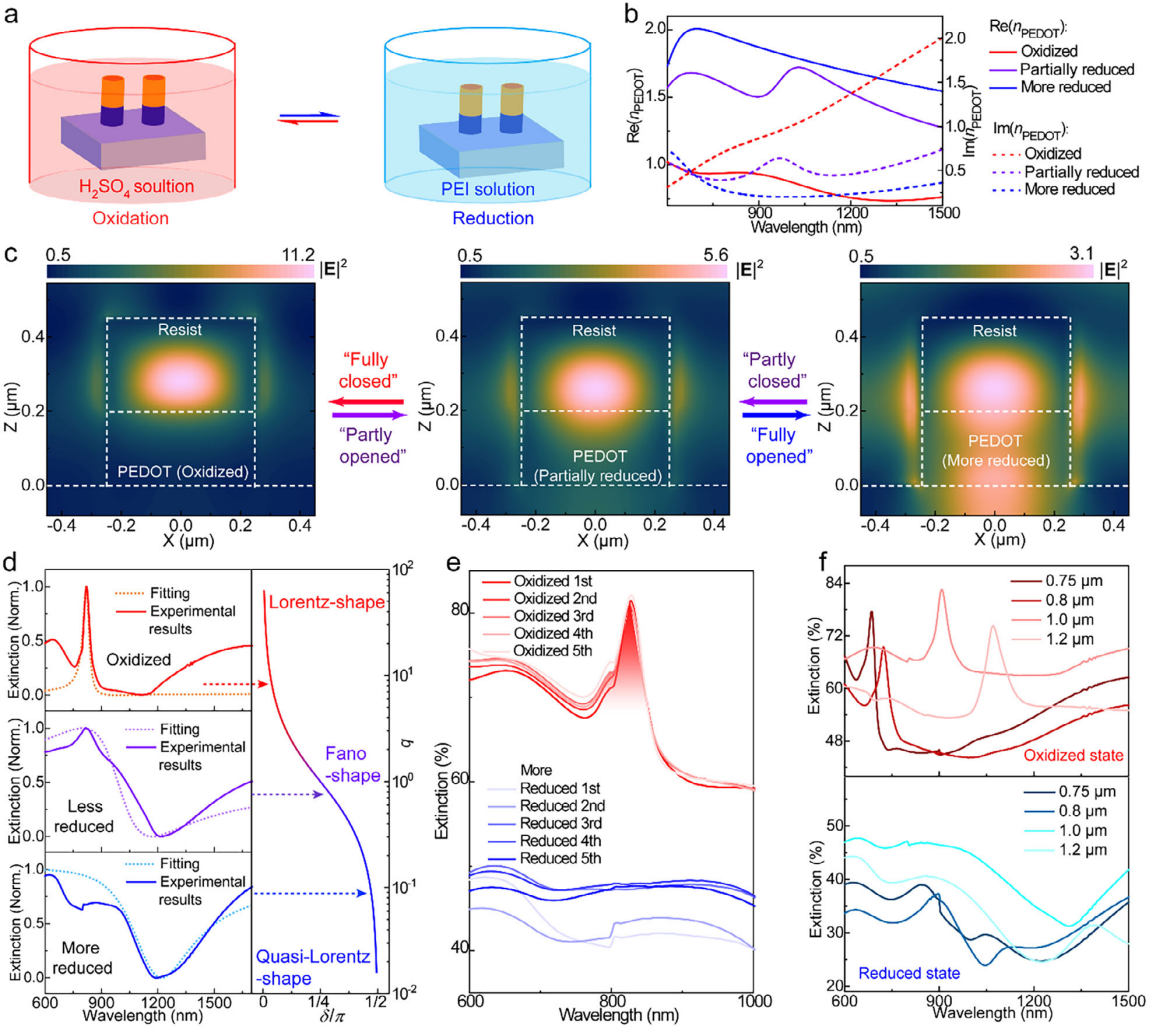
Switching of visible and near‐IR CLRs by redox reactions. (a) Illustration of redox reaction conditions of PEDOT nanoantennas. (b) Refractive index of PEDOT in oxidized, partially reduced and more reduced states, respectively. (c) The electrical field distributions of Die‐PEDOT‐Nano in the hexagonal arrays (*r* = 0.9 µm), including the oxidized (the conducting polymer gate in “fully closed” states), partially reduced (the conducting polymer gate in “partly opened” or “partly closed” state), and more reduced states (the conducting polymer gate in “fully opened” state). (d) Normalized extinction spectra (experimental results) of Die‐PEDOT‐Nano arrays with PEDOT in oxidized, partially reduced, and more reduced states, respectively. The fitting with Fano resonance models gives the Fano asymmetry parameter *q* = cot *δ*. (e) Measured extinction spectra of Die‐PEDOT‐Nano arrays (*d* = 0.5 µm and *r* = 0.9 µm) during redox cycles in 5 times. (f) Measured extinction spectra of Die‐PEDOT‐Nano arrays with other periods and nanoantenna diameters in a redox cycle. The periodicities include *r* = 0.75 µm (*d* = 0.38 µm), 0.8 µm (*d* = 0.41 µm), 1.0 µm (*d* = 0.53 µm), and 1.2 µm (*d* = 0.61 µm).

We demonstrate that the visible/NIR CLRs can be fully switched off and on again by tuning the redox state of the PEDOT layer in the nanocylinders. Although the dielectric nanocylinders maintain their permittivity during redox tuning, the transformation of PEDOT between metallic (oxidized) and dielectric (reduced) states enables tuning of the light confinement within the dielectric nanocylindersand thereby the nonlocal Mie resonances. Figure 5a illustrates the redox conditions including oxidation with sulfuric acid and reduction with poly(ethylenimine) (PEI). Figure 5b presents the refractive index of PEDOT (*n*_PEDOT_) in its oxidized state, partially reduced state, and more reduced state (reduction degree higher than the partially reduced state but lower than fully reduced state as reported before [15]). Upon reduction, the real part Re(*n*_PEDOT_) increases from 0.9–1.0 in its oxidized state to 1.80–1.95 and the imaginary part Im[*n*_PEDOT_] decreases from 0.8–0.9 to 0.2–0.3. These changes confirm the transformation from metallic to dielectric behavior. This affects the possibility for optical confinement as can be investigated from near‐field distributions of Die‐PEDOT‐Nano arrays in the different redox states (*d* = 0.5 µm and *r* = 0.9 µm). In the oxidized PEDOT state, the electrical field is efficiently concentrated within the dielectric nanocylinders (Figure 5c), and the magnetic field can be also blocked by the PEDOT nanoantennas (Figure S63a). These features reflect that the oxidized PEDOT resemble a fully closed gate that supports light confinement. When transforming the PEDOT into a partially reduced state, |**E**|^2^ within the dielectric is lowered by 2 times, and both the electric (Figure 5c) and magnetic fields (Figure S63c) are expanded into the PEDOT layer. These changes indicate that the partially reduced PEDOT is analogous to a partly opened/closed gate that limits light confinement. In the more reduced state, the PEDOT serves as a fully opened gate to form a leaky channel for both the electric (Figure 5c) and magnetic fields (Figure S63e), which eliminates the light confinement within the nanoantennas. Thus, the light confinement can be dynamically controlled by the conducting polymer gate, transformable between opened and closed states.

Based on the tuneability of light confinement, we verified associated changes in the extinction of Die‐PEDOT‐Nano arrays (*r* = 0.9 µm, *d* = 0.48 µm) through a Fano resonance model [35, 36] (Figure 5d, Figure S64). In such model, the CLR at *λ*_r_ = 828 nm is applied for the discrete state, and the Fano invisibility [35, 37] at *λ*_C_ = 1200 nm (Figure S65) serves as the continuum. In the oxidized PEDOT state, we observed a sharp resonance at *λ*_r_, and the Fano analysis exhibits a low phase shift (*δ* ≈ 0.05π) and a high asymmetry factor (*q* = 6.84) for a Lorentz shape. This feature is related to very low damping relaxation (γ_Fano_ ≈ 0.07 eV at *λ*_r_), consistent with electric dipole resonance confined in the dielectric nanocylinders due to the blockage of the optical leakage channel. Reducing the PEDOT broadens the resonance (in partially reduced state) and even eliminates it (in the more reduced state), and the Lorentz‐shaped resonance converts into Fano (δ ≈ 0.31π and *q* = 0.66) and quasi‐Lorentz shapes (*δ* ≈ 0.53π and *q* = 0.1), respectively. The results can be understood as the case that the contribution of the discrete state becomes minor when gradually enhancing γ_Fano_ by 1–2 orders of magnitude, agreeing with the diminishment of light confinement and the formation of the electromagnetic leaky channel into the substrate. Furthermore, the above Fano and Quasi‐Lorentz shapes can be recovered to the Lorentz shape upon reoxidation reactions. Thus, such Fano resonance can be modulated in a whole *q*‐scope in extinction spectra, which facilitates the switchable function for this nonlocal Mie resonance.

Figure 5e demonstrates reversible switching of a nonlocal Mie resonance (*r* = 0.9 µm) by transforming the conducting polymer gate between closed (oxidized) and opened (more reduced) states for several cycles. The difference in extinction depth (between Lorentz and quasi‐Lorentz shapes) is 30‐35 percentage points, larger than that for the localized surface plasmon resonance around ∼1800 nm (10–20 percentage points, Figure S66). Importantly, reduction fully eliminates the CLR and completely recovers it upon oxidation. After 4–5 times of redox cycles, the oxidation still recovers the diffraction orders of the RAs in angle‐dependent extinction spectra (Figure S67), and *Q*‐factors are maintained at *Q* = 23, which confirm the reappearance of the nonlocality. The slight increase in background extinction in the reduced state with number of cycles may be due differences in reduction efficiency. The switchable CLRs can be provided also at other wavelengths using other Die‐PEDOT‐Nano arrays (*r* = 0.75, 0.8, 1.0 and 1.2 µm, respectively, Figure 5f, Figures S69, S70). Moreover, the resonance wavelength can cover the visible region (*λ*_r_ < 740 nm, *r* = 0.75 and 0.8 µm). To our best knowledge, this is the first all‐organic nanoantenna arrays that realize dynamically switchable resonances in the visible wavelength range.

## 6 Conclusion

This work demonstrates switchable visible/NIR‐I CLRs from all‐organic metasurfaces based on PEDOT acting as optical leakage gate. The resonance wavelength is not limited to the negative permittivity region of PEDOT, but could span 640‐1200 nm by varying the array periodicity. *Q*‐factors can be as high as 25, greatly exceeding previous organic metasurfaces in this range. The high‐*Q* CLRs originate from nonlocal coupling based on dipole radiation at the air superstate (first order) and second‐order diffraction grating effect within the dielectric nanocylinders, which is related to the effect of light confinement induced by the quasi‐metallic PEDOT. Reducing the PEDOT to its low‐conducting state opens the optical gate and forms an electromagnetic leaky channel which eliminate light confinement thanks to the low refractive index of the dielectric polymer. As a result, the concept facilitates complete and reversible switching of visible/NIR CLRs. The work contributes significantly to the development of organic metasurfaces by simultaneously addressing key challenges related to low *Q*‐factors and providing dynamically tunable resonance at short wavelengths.

## 7 Experimental Section

### 7.1 Calculations of *S*, *α*_iso,_ and *∆*

In CLR‐matching conditions of dielectric nanocylinder arrays, the electrical dipole sum (*S*) was calculated through the equation:

(1) $$S=\sum_{j}^{N}\exp\left(ikr_{j}\right)\left[\frac{(1-ikr_{j})(3\mathrm{co}s^{2}\theta_{j}\;-\;1)}{r_{j}^{3}}+\frac{k^{2}\mathrm{si}n^{2}\theta_{j}}{r_{j}}\right]$$

where *r_j_* denotes the center‐to‐center distance between two dielectric nanocylinders, *θ*_j_ denotes the angle between the electric polarization and the lattice vector of dielectric nanocylinder arrays, *N* is the number of other dielectric nanocylinders in the lattice, **k** is the wave vector with the magnitude *k* = 2*πn*_eff_/*λ*, *n*_eff_ is the effective refractive index of CLRs. In this work, as CLR occurs at the air superstrate, here we set *n*_eff_ = 1 to calculate *S*.

Considering that the Mie resonances of Die‐PEDOT‐Nano arrays only originate from the electric dipole resonance (without magnetic dipole and quadrupole resonances), here we calculated the isolated electric polarizability (*α*_iso_) of dielectric nanocylinder units only from the first‐order electric Mie coefficient [38], which is analogous to plasmonic dipolar resonances. Based on the preconditions of pure electric dipole resonance (as the first‐order electric Mie coefficient), the mathematical equation of *α*_iso_ of dielectric nanoantennas is almost the same as those used for plasmonic nanoantennas (as shown in the references [38, 39]), which serves as an approximation of *α*_iso_ of the dielectric nanoantenna units. *α*_iso_ of a single dielectric nanocylinder was then calculated with the modified long‐wavelength approximation [20]:

(2) $$\alpha_{iso}=\alpha_{s}{\left[1-\frac{k^{2}}{a}\alpha_{s}-\frac{2}{3}ik^{3}\alpha_{s}\right]}^{-1}$$

where *α*_s_ demotes the static non‐corrected isolated polarizability of a dielectric nanocylinder:

(3) $$\alpha_{s}=\frac{a^{2}b}{3}\frac{\varepsilon_{d}-\;\varepsilon_{s}}{\varepsilon_{s}+L(\varepsilon_{d}-\;\varepsilon_{s})}$$

where *a* and *b* are the long and short semiaxes’ lengths of the oblate spheroid, respectively, *L* is evaluated as a geometric factor, and *ε*_d_ and *ε*_s_ are the relative permittivity of the dielectric material and the surrounding medium, respectively. Here we calculated the *ε*_s_ = *S*_air_ *ε*_air_ + *S*_PEDOT_ *ε*_PEDOT_ by considering the square ratio of air (*S*_air_) and the square ratio of the interface between PEDOT (permittivity as *ε*_PEDOT_) and dielectric nanocylinder (*S*_PEDOT_). The *ε*_air_ is 1.

### 7.2 Thin‐Film Deposition of PEDOT

The preparation of PEDOT film (acid‐treated PEDOT:Tosylate [PEDOT:Tos]) is referred to previously reported literature [15]. Briefly, we spin‐coated the oxidant reagent (the mixture with 2 g of tri‐block PEG‐PPG‐PEG co‐polymer [from Sigma Aldrich company], 2 g of Clevios C‐B 54 V3 [from Heraeus company in Germany], and 5 g of absolute ethanol) on precleaned glass substrates under a spinning speed of 1500 RPM and then annealing these substates at at 70°C for 1 min. To perform vapor phase polymerization (VPP), we placed these oxidant‐coated films in a vacuum chamber to react with EDOT vapor (formed through heating liquid EDOT under vacuum) for 40 min. After the VPP reaction, unreacted residues were removed by rinsing these films (with a thickness of ∼0.2 µm) with ethanol, followed by drying with nitrogen gas. To ensure the oxidized (dopped) state, we soaked these PEDOT films in sulfuric acid solution (concentration: 3 m) for 10 min at room temperature, followed by washing with deionized (DI) water, drying with nitrogen flow and annealing on a hot plate (140°C).

### 7.3 Periodic Array Fabrication and Structural Characterization

The PEDOT films were spin‐coated with ma‐N 2405 negative resist (Micro Resist Technology) at 3000 rpm, followed by baking the resist at 125°C for 2–3 min to achieve the resist thickness of 0.5∼0.6 µm. We used Raith Voyager 100 electron beam lithographer (at 50 kV) to expose specific regions for ordered periodic arrays. The beam current was 1.5∼1.6 nA, and the area dose and curved elements were set as 160 µC cm^−2^. Because PEDOT:Tos film can ensure sufficient charge dissipation during e‐beam lithography, we did not additionally add a conductive layer. After EBL procedures, we developed the negative resist for 90–120 s by using ma‐D 525 developer (Micro Resist Technology), followed by soaking in DI water for 30 s and blow‐drying with nitrogen guns. Then we dry‐etched PEDOT film with ma‐N 2405 mask through RIE Vacutec etcher, where the reactive oxygen plasma was set under the conditions of 50 W power, 150 mTorr pressure and 125 s dry‐etching time. After the dry‐etching, the PEDOT nanodisks with the ma‐N 2405 resist on the top were achieved in a periodic array. The final periodic structures were visualized without any additional coating using Carl Zeiss Sigma 500 scanning electron microscope operating at 1 kV voltage with SE2 detector.

### 7.4 Preparation of Highly Disordered Die‐PEDOT‐Nano Arrays

The whole process of preparing highly disordered arrays is shown in Figure S20. The PEDOT films were spin‐coated with polyvinyl alcohol (PVA) solution (solvent: DI water; concentration: 2 g ml^−1^), followed by annealing at 140°C for 5 min. Then we spin‐coated the ma‐N 2405 negative resist (at 3000 rpm) on the PVA layer. The following procedures from baking the resist to EBL process were the same as the preparation of ordered periodic arrays. After EBL process, we developed the sample (using the developer ma‐D 525) for 90 s to achieve a highly disordered array of negative resist. Then we performed the dry‐etching under the same conditions of ordered periodic arrays to achieve the highly disordered arrays consisting of PEDOT and negative resist nanocylinders.

### 7.5 Redox‐Cycling

The reduction reaction was performed with branched poly(ethylenimine) (PEI) solution (*M*_w_ ≈ 800, from Sigma–Aldrich company), where DI water is the solvent and the volume ratio of PEI to DI water is about 1:3∼1:4. For more reduced state of PEDOT, we soaked the sample in PEI solution at 90∼100°C for 15 min, and then used the nitrogen gun to remove the PEI solution on the metasurfaces. Furthermore, we cannot use DI water to remove PEI as DI water can transform the more reduced state to the partially reduced state (still showing a CLR peak of Mie resonance). The residue of PEI cannot be completely removed by heating the sample at 120°C (like reported in previous works [15]), because the PEI vapor can react with the negative resist and the dielectric nanocylinders were completely consumed so that such nonlocal Mie resonance cannot be recovered. Luckily, the slight residue of PEI could be removed when soaking the sample in sulfuric acid solution (concentration: 1 M; for 5 min) that transforms the PEDOT nanoantennas in oxidized state, and the CLR is recovered.

### 7.6 Extinction Spectra Characterization

We used UV‐Vis‐NIR spectrophotometer (Perkin Elmer Lambda 900) to acquire extinction spectra in the wavelength range of 0.6∼2.2 µm. For angle‐dependent extinction spectra, we added an angle‐resolution transmission accessory and an aperture (diameter of 5 mm). A polarizer was also used to control the TM and TE polarizations. As the spectral intensity was much lower when using the aperture and polarizer, we conducted a smoothing process for angle‐dependent extinction spectra (based on the Savitzky‐Golay method, the second polynomial order, 15 points of windows, during which the peak wavelength does not change). Such smoothing process did not change the resonance wavelength at various incident angles.

### 7.7 Ellipsometry Characterization

The ellipsometry data of the acid‐treated PEDOT:Tos films in partially reduced (soaking the sample in PEI solution at 20°C for 30 min, followed by washing with DI water) and more reduced states (soaking the sample in PEI solution at 90°C for 5 h followed by drying with nitrogen flow and heating at 150°C for 30 min, which enables to fully remove PEI from the PEDOT surface) were measured by two different ellipsometers corresponding to UV‐vis‐NIR (400–1690 nm) and MIR (1690–10000 nm) wavelength regions. The PEDOT film was deposited on silica substrates (with an ultrathin SiO_2_ layer of 200 nm on the top of silica layer). All ellipsometry spectra were measured at 20°C. The UV–vis–NIR wavelength regions were measured using a J. A. Woollam Co. RC2 spectroscopic ellipsometer under incident angles of 45°, 55°, 65°, and 75°. The MIR wavelength regions were tested using a J. A. Woollam Co. IR‐VASE spectroscopic ellipsometer under incident angles of 50°, 60°, and 70°. The anisotropic Drude–Lorentz model [8, 40] was used to fit and analyze the refractive index of PEDOT film in different redox states, based on the CompleteEASE (J. A. Woollam Co.) software, where the parameters in Drude‐Lorentz models were confirmed by Matlab software. The ellipsometry data (within 400–1690 nm) of the negative resist ma‐N 2405 (also exposed through EBL processes) was acquired by the J. A. Woollam Co. RC2 spectroscopic ellipsometer under incident angles of 45°, 55°, 65°, and 75°, and the Cauchy model was used for fitting the ellipsometry data through the CompleteEASE (J. A. Woollam Co.) software. The refractive index of PBFDO and the oxidized state of aicd‐treated PEDOT:ToS is referred to previous work [10, 15].

### 7.8 Optical Numerical Simulations

FDTD simulations, including far‐field (extinction spectra) and near‐field (electrical field distribution) properties, were performed through Ansys Lumerical FDTD software (https://www.ansys.com/products/optics/fdtd). The PEDOT‐based periodic arrays were constructed through periodic boundaries (Bloch type) along *X*‐ and *Y*‐axis, and PML boundaries were set along *Z*‐axis, which is parallel to the wave vector of incident light (by using a Bloch/periodic type plane wave). As two‐dimensional power monitors that achieve near‐field distributions were set within XZ plane, the electric vector was set as X‐axis for TM mode, and Y‐axis for TE mode. The glass substrate was constructed by a dielectric material with the constant refractive index *n*_sub_ = 1.5. The *n*_PEDOT_ was referred to the analysis of anisotropic Drude–Lorentz model from the experimental ellipsometry data and *n*_die_ of negative resist was referred to the fitting with Cauchy model. For simulations of angle‐dependent extinction spectra, we used a BFAST type plane wave as the light source. The mesh size of PEDOT nanoantennas, PEDOT film and dielectric nanocylinders were set as 20 × 20 × 10 nm^3^. For the localized model with a single nanoantenna (*d* = 0.56 µm), we used PML boundaries along all directions and selected Total‐Field Scattered‐Field (TFSF) source as the light source. The cross‐section spectra (Figure S3) were achieved by box‐like power detectors.

## Funding

European Research Council (Consolidator Grant, 101086683); Knut and Alice Wallenberg Foundation (Wallenberg Academy Fellow, 2019.0163, 2020.0301); Swedish Research Council (VR, Consolidator Grant, 2020‐00287); Swedish Government Strategic Research Area in Materials Science on Functional Materials at Linköping University (Faculty Grant SFO‐Mat‐LiU No. 2009 00971).

## Conflicts of Interest

The authors declare no conflict of interest.

## Supporting information




**Supporting File**: adma71848‐sup‐0001‐SuppMat.docx

## Data Availability

The data that support the findings of this study are available in the supporting information of this article and at Zenodo at https://doi.org/10.5281/zenodo.17979287.
