# Supplementary material for: Dynamic Optical Lattices Through Conducting Polymer‐Gated Confinement
Source: Adv Mater. 2025 Dec 22;38(10):e20674. doi: 10.1002/adma.202520674 (PMC12910422; doi:10.1002/adma.202520674)
Supplement: Supplementary file 1 — Supporting File: adma71848‐sup‐0001‐SuppMat.docx [file ADMA-38-e20674-s001.pdf]

Supporting Information

Dynamic Optical Lattices through Conducting Polymer-Gated Confinement

*Dongqing Lin, Yulong Duan, Suraya Kazi, Magnus P. Jonsson**

Supporting Note 1: Theoretical models for nonlocal organic Mie resonances.

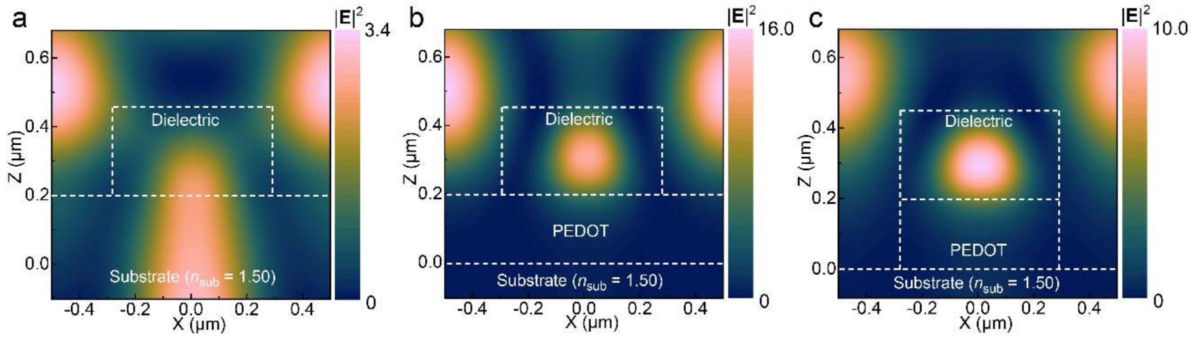

Figure S1. Transverse-electric (TE) near-field distributions at the wavelength of 870-900 nm. (a) (b) (c) Hexagonal arrays consisting of pure dielectric nanoantennas (Die-Nano), dielectric nanoantennas-PEDOT film (Die-PEDOT-Film), and PEDOT-dielectric hybridized nanocylinders (Die-PEDOT- Nano), respectively. The Die- Nano has a diameter d = 0.56 μm and a height h = 0.25 μm. The refractive index of dielectric material is n_{die} = 1.60, and the refractive index of substate is n_{sub} = 1.50. In (b), the thickness of PEDOT film is 0.20 μm. In (c), the PEDOT patterned in the nanocylinders has a diameter d = 0.56 μm and a height h = 0.20 μm. These nanoantennas were spaced in the hexagonal arrays, where the periodic distance is r = 1.0 μm. The directions of wave vector and electric vector are along Z-axis and Y-axis, respectively.

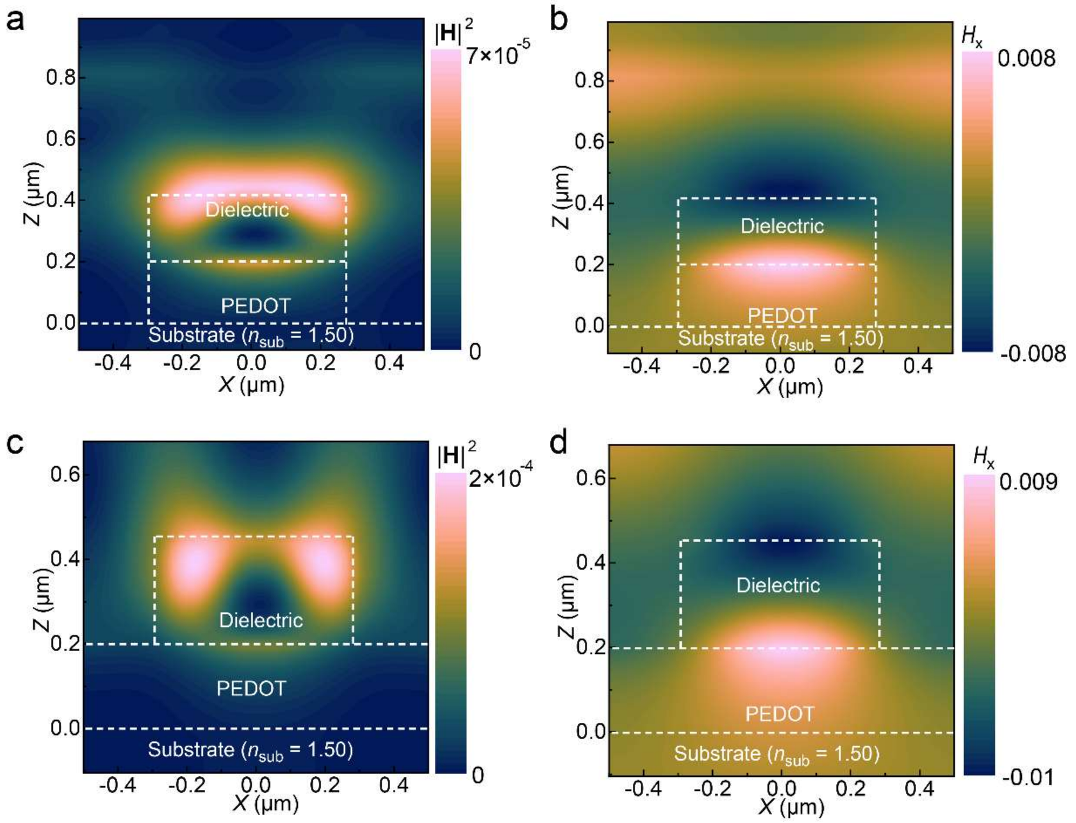

Figure S2. Magnetic near-field distribution of Die-PEDOT-Nano and Die-PEDOT-film arrays at individual resonance wavelengths. (a) (b) Intensity of magnetic field ($|\mathbf{H}|^2$) and the magnetiude of magnetic field along X-axis (H_x) of Die-PEDOT-Nano arrays, respectively. The near-field distribution is based on the resonance wavelength $\lambda_r \approx$ 900 nm. (c) (d) Intensity of magnetic field ($|\mathbf{H}|^2$) and the magnetiude of magnetic field along X-axis (H_x) of Die-PEDOT-

Film arrays, respectively. The near-field distribution is based on the resonance wavelength $\lambda_r \approx$ 870 nm. The directions of wave vector, electric vector and magnetic vector of incident light are along *Z*-axis, *Y*-axis and *X*-axis, respectively. These near-field distributions are based on the TE mode. In (a) (b), the magnetic field has a circular pattern rather than being confined within the dielectric nanocylinders. Thus, such Mie resonance can be assigned to electric dipole[1].

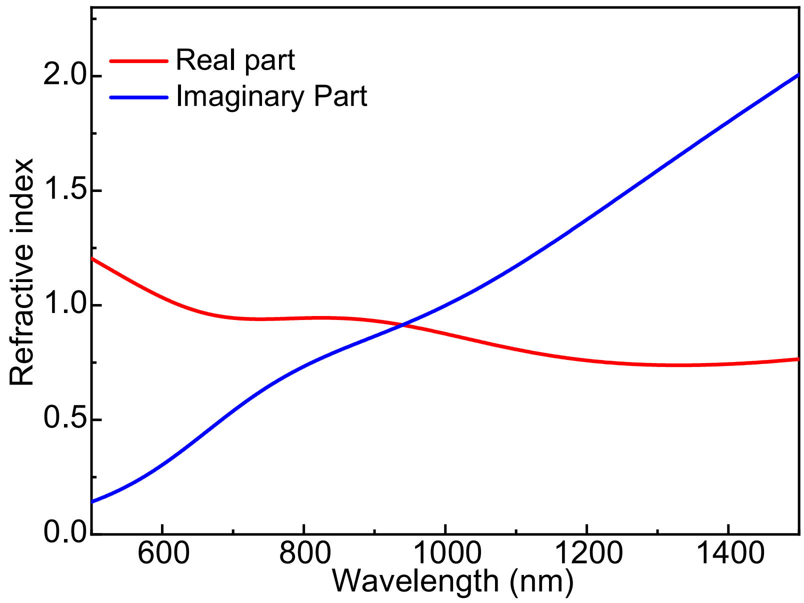

Figure S3. Refractive index of acid-treated PEDOT:FeToS. These data were referred to the previous work[2].

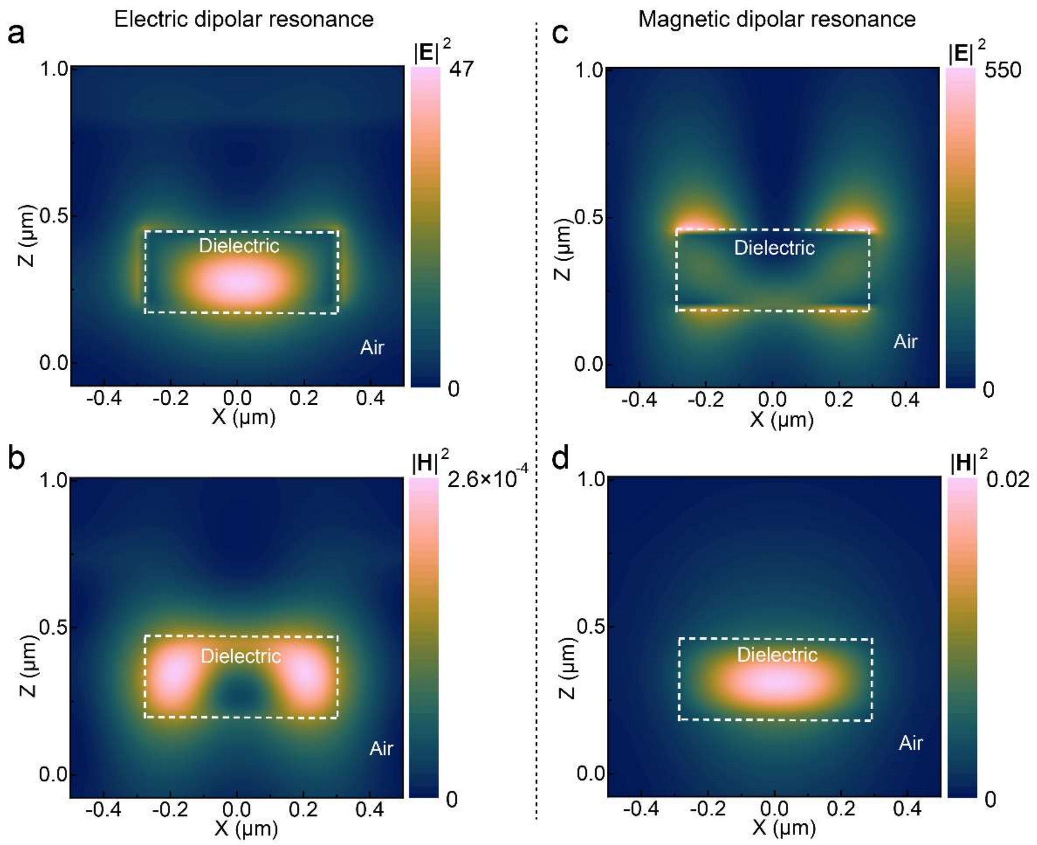

Figure S4. Near-field distribution of Die-Nano in air. (a) (b) Intensity of electric ($|\mathbf{E}|^2$, in TM mode) and magnetic field ($|\mathbf{H}|^2$, in TE mode), respectively, under electric dipolar resonance at the wavelength $\lambda_r \approx 905$ nm. In these two figures, the electric field is confinemd within the cenral region of dielectric nanocylinders but magnetic field is distributed in a circular type around the edge of dielectric nanocylinders, which are consistent with the feature of electric dipole resonance[1]. (c) (d) Intensity of electric ($|\mathbf{E}|^2$, in TM mode) and magnetic field ($|\mathbf{H}|^2$, in TE mode), respectively, under magnetic dipolar resonance at the wavelength $\lambda_r \approx 917$ nm. In these two figures, the magnetic field is confined within the central region of dielectric nanocylinders but electric field is distributed at the outer edge of dielectric nanocylinders, which agree with the feature of magnetic dipole resonance[1]. In this model, we removed the substrate so that these Die-Nano arrays are directly arranged in air. In (a) and (c), the directions of wave vector, electric vector and magnetic vector of incident light are along *Z*-axis, *X*-axis and *Y*-axis, respectively. In (b) and (d), the directions of wave vector, electric vector and magnetic vector of incident light are along *Z*-axis, *Y*-axis and *X*-axis, respectively.

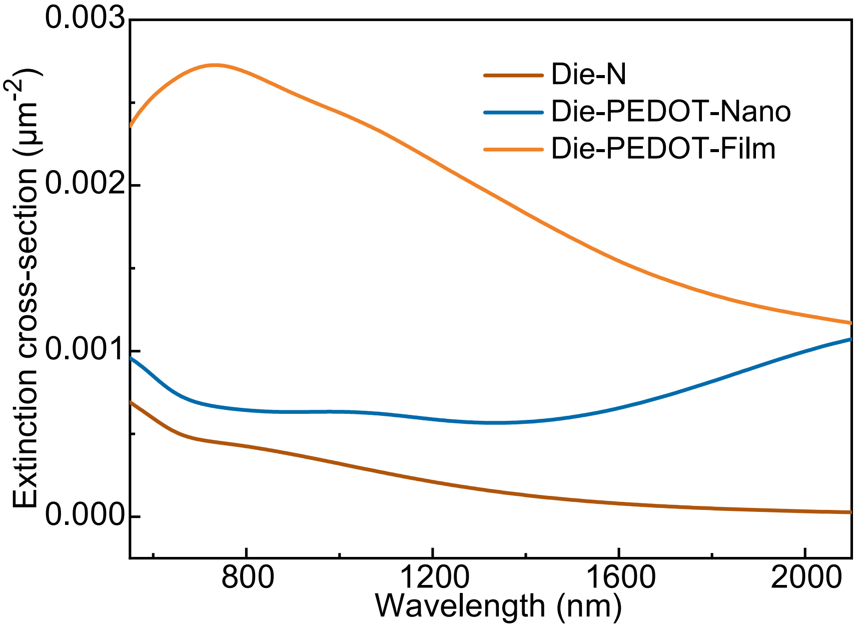

Figure S5. Simulated extinction cross-section of a single nanoantenna. The Die-Nano, Die-PEDOT-Nano, and Die-PEDOT-Film only represents the single nanostructures rather than arrays, and the resonance peak only represents the localized mode. The Die-Nano has a diameter d = 0.56 μm and a height h = 0.25 μm. The refractive index of dielectric material is n_{die} = 1.60, and the refractive index of substate is n_{sub} = 1.50. The thickness of PEDOT film is 0.20 μm. The PEDOT patterned in the nanocylinders has a diameter d = 0.56 μm and a height h = 0.20 μm. The extinction spectra were calculated through box-like detectors.

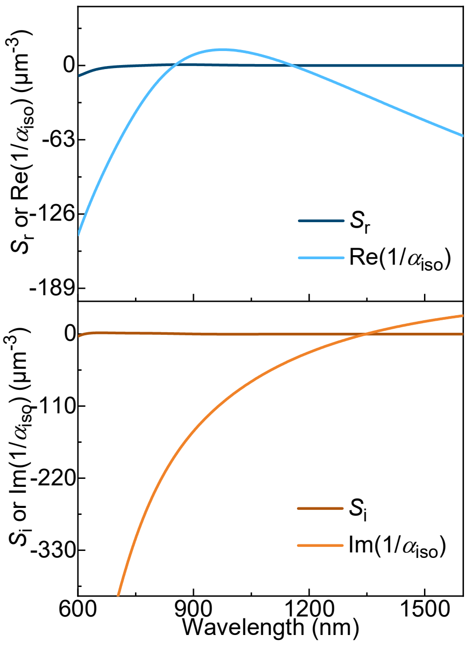

Figure S6. *S*-(1/α_{iso}) analysis of Die-PEDOT-Film arrays. The array factor (S) includes the real part (S_r) and the imaginary part (S_i), similar to 1/α_{iso} (real part: Re[1/α_{iso}]; imaginary part: Im[1/α_{iso}]). In this model, the refractive index of acid-treated PEDOT:ToS film (Figure S3), serving as the substrate for CLRs, is used in the calculation of S. The dielectric nanocylinders have a diameter d = 0.56 μm and a height h = 0.25 μm. The thickness of PEDOT film is 0.2 μm.

According to the equations: $S = \sum_j^N \exp(i\mathbf{k}r_j)\,[\frac{(1-i\mathbf{k}r_j)(3\cos^2\theta_j - 1)}{r_j^3} + \frac{\mathbf{k}^2\sin^2\theta_j}{r_j}]$ and the wave vector $|\mathbf{k}|$

$= 2\pi n_{sub}/\lambda$ (see Methods), the large imaginary part of refractive index (or extinction coefficient) in n_{sub} can lead to the real part of $\exp(i\mathbf{k}r_j) < 0$, which sharply lowers the magnitude of S. Similarly, the Im(n_{PEDOT}) significantly decays the amplitude of S to 0 ($S_r \approx S_i \approx 0$) if using PEDOT film as the S medium. Even so, we still observed Re($1/\alpha_{iso}$) = $S_r \approx 0$ at the wavelength of 855 nm, agreeing with $\lambda_r \approx 860$ nm in the simulated extinction spectrum.

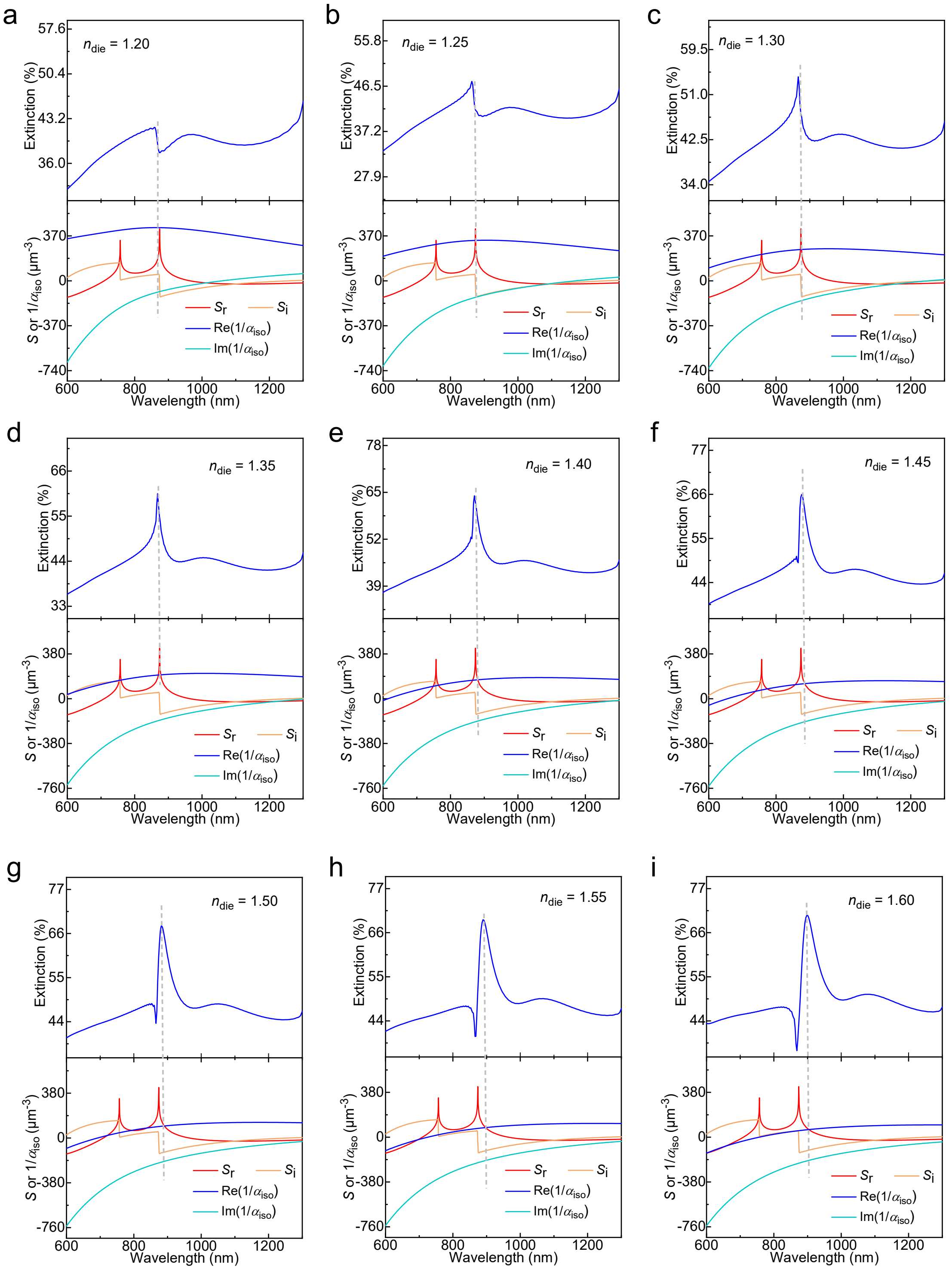

Figure S7. Analysis of CLR-matching conditions and extinction spectra for hexagonal Die-PEDOT-Nano arrays with different refractive index of dielectric nanocylinders (n_{die} ranging from 1.20 to 1.60). The acid-treated PEDOT:ToS was selected for the part of conducting polymer nanoantennas. The extinction spectra were achieved via FDTD simulations.

In all panels, dielectric nanocylinders have a diameter d = 0.56 μm and a height h = 0.25 μm. The height of PEDOT layer is 0.2 μm. The periodic distance of Die-PEDOT-Nano arrays was set as r = 1.0 μm.

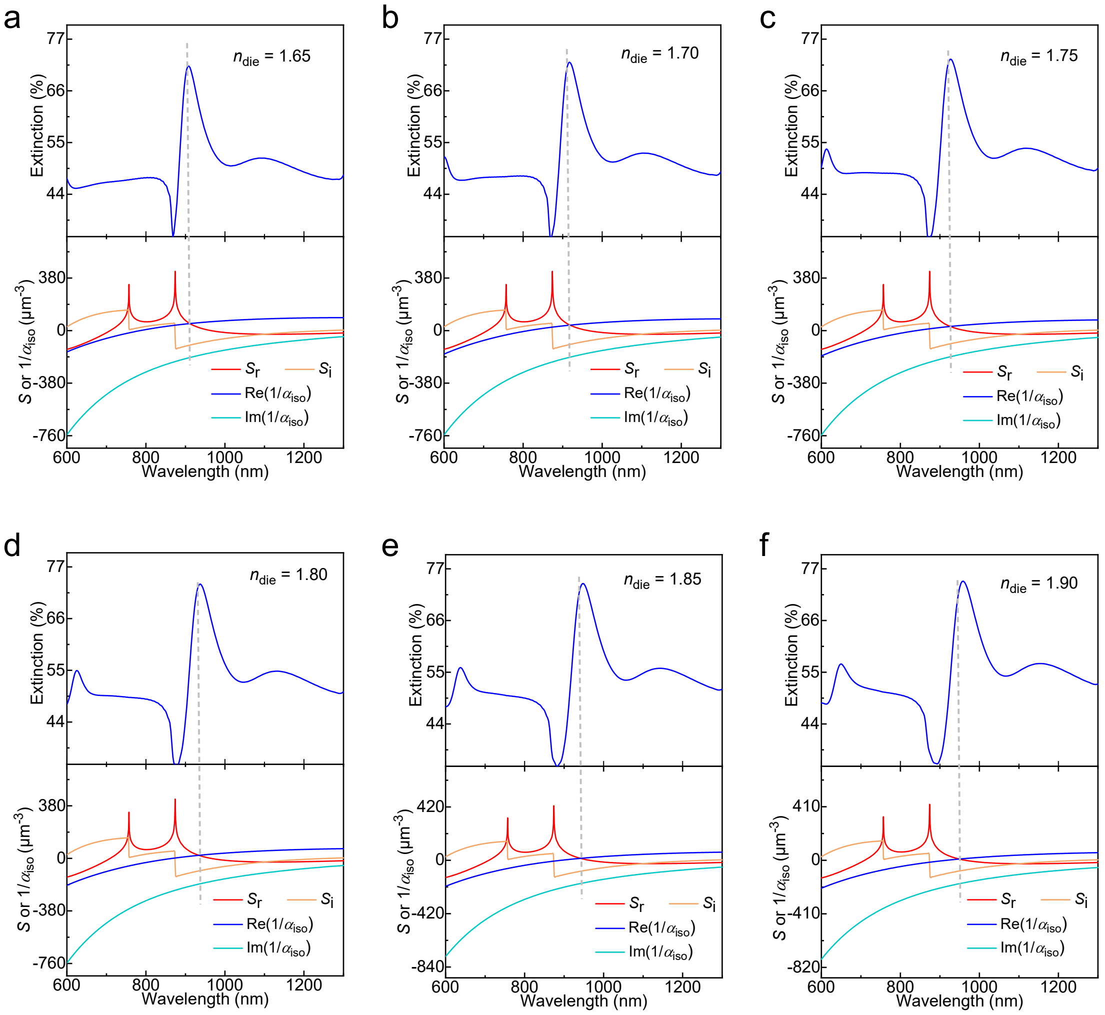

Figure S8. Analysis of CLR-matching conditions and extinction spectra for hexagonal Die-PEDOT-Nano arrays with different refractive index of dielectric nanocylinders (n_{die} ranging from 1.65 to 1.90). The acid-treated PEDOT:ToS was selected for the part of conducting polymer nanoantennas. The extinction spectra were achieved via FDTD simulations. In all panels, dielectric nanocylinders have a diameter d = 0.56 μm and a height h = 0.25 μm. The height of PEDOT layer is 0.2 μm. The periodic distance of Die-PEDOT-Nano arrays was set as r = 1.0 μm.

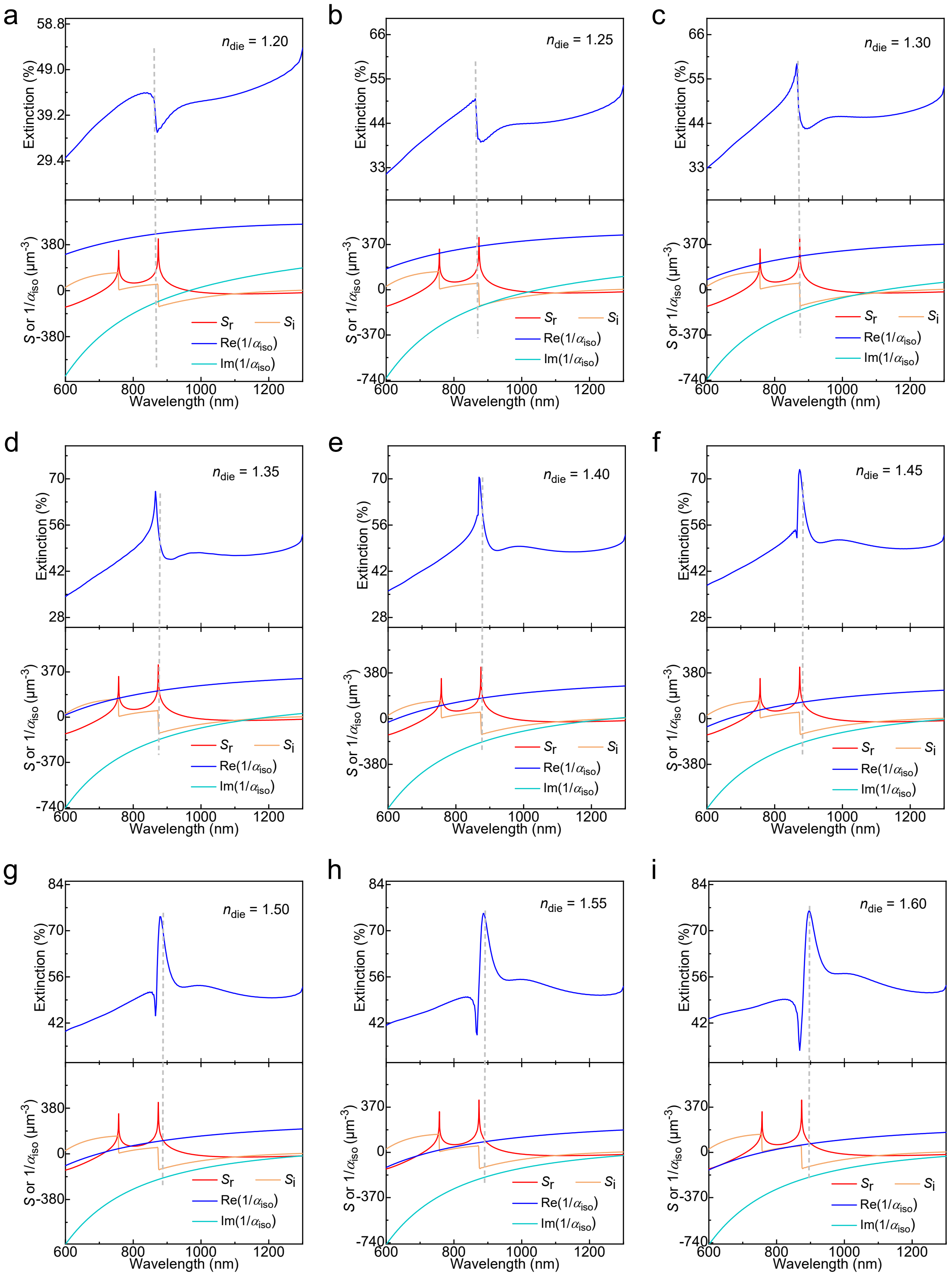

Figure S9. Analysis of CLR-matching conditions and extinction spectra for hexagonal Die-PEDOT-Nano arrays with different refractive index of dielectric nanocylinders (n_{die} ranging from 1.20 to 1.60). The PEDOT:Sulf was selected for the part of conducting polymer nanoantennas. The extinction spectra were achieved via FDTD simulations. In all panels, dielectric nanocylinders have a diameter d = 0.56 μm and a height h = 0.25 μm. The height of PEDOT layer is 0.2 μm. The periodic distance of Die-PEDOT-Nano arrays was set as r = 1.0 μm.

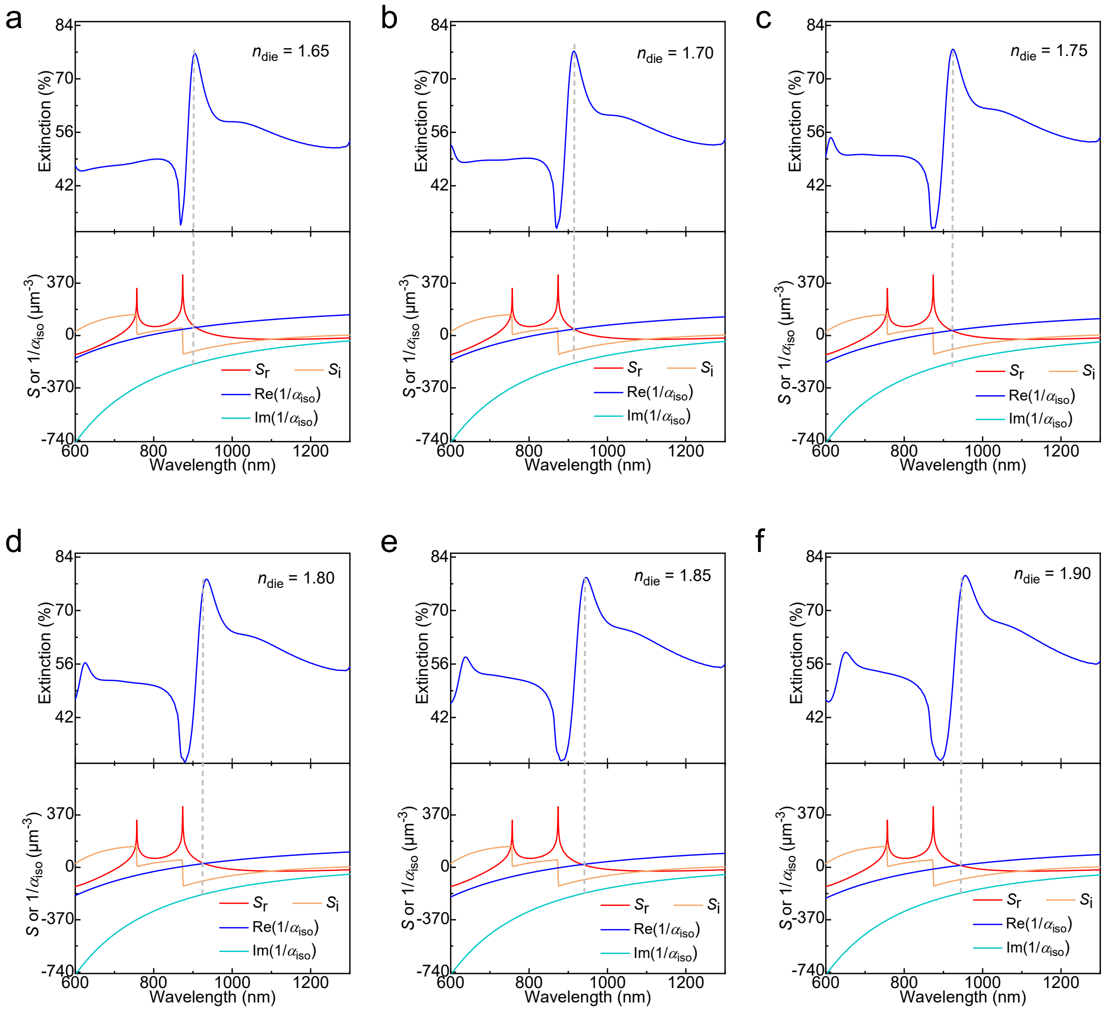

Figure S10. Analysis of CLR-matching conditions and extinction spectra for hexagonal Die-PEDOT-Nano arrays with different refractive index of dielectric nanocylinders (n_{die} ranging from 1.65 to 1.90). The PEDOT:Sulf was selected for the part of conducting polymer nanoantennas. The extinction spectra were achieved via FDTD simulations. In all panels, dielectric nanocylinders have a diameter d = 0.56 μm and a height h = 0.25 μm. The height of PEDOT layer is 0.2 μm. The periodic distance of Die-PEDOT-Nano arrays was set as r = 1.0 μm.

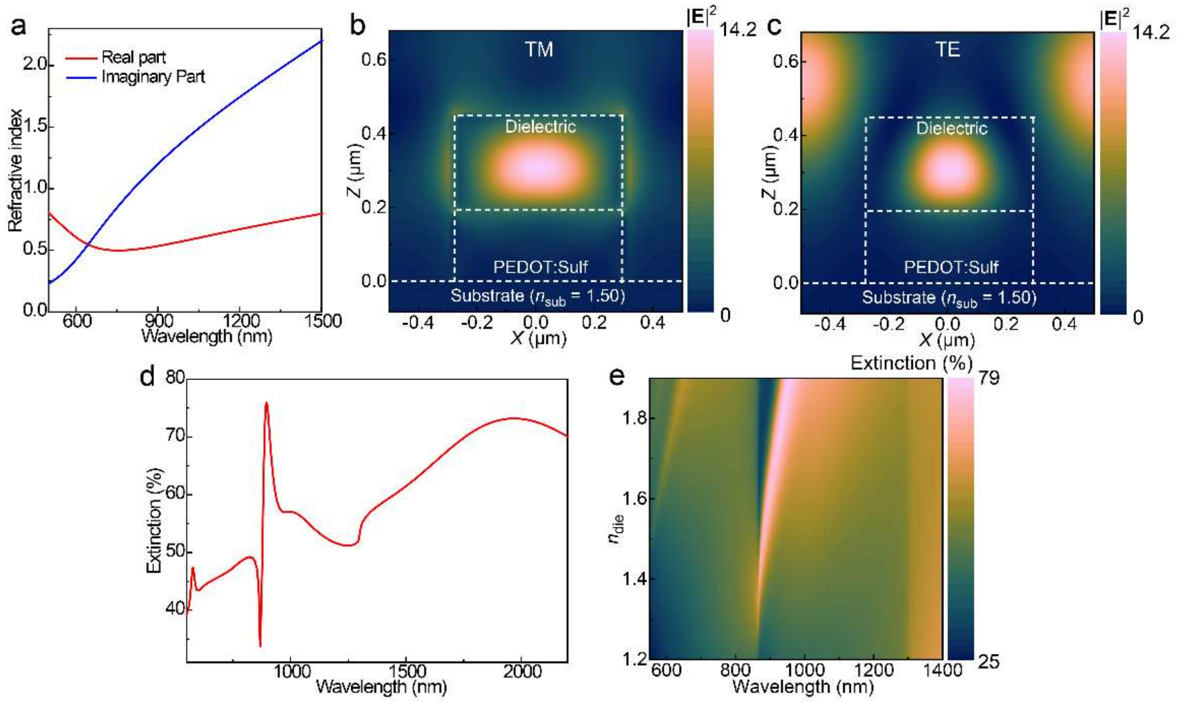

Figure S11. The effect of blocking gate from PEDOT:Sulf nanoantennas. (a) Refractive index of PEDOT:Sulf. The real part of refractive index ranges from 0.5 to 0.7 in the wavelength region of 700-1300 nm (Referred to the previous work[3]). (b) (c) TM and TE near-field distributions (near 900 nm) of hexagonal arrays consisting of Die-PEDOT-Nano units, respectively. The dielectric nanocylinder has a diameter d = 0.56 μm and a height h = 0.25 μm. The height of PEDOT layer is 0.2 μm. (d) Simulated extinction spectrum of Die-PEDOT-Nano array with the periodicity r = 1.0 μm. (e) n_{die}-dependent extinction spectra of the Die-PEDOT-Nano array model. The sharp Mie resonance could be observed if $n_{die} \geq 1.4$.

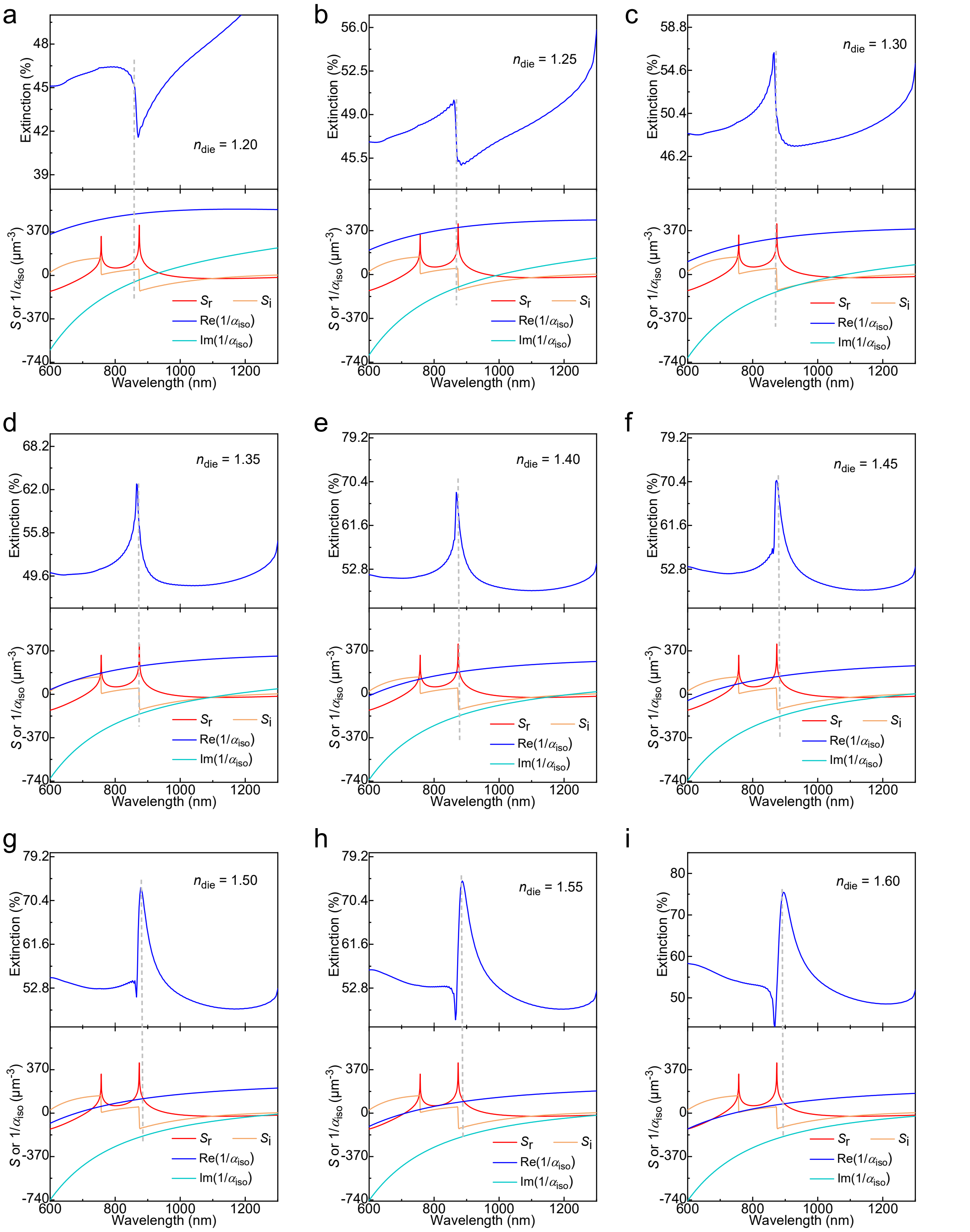

Figure S12. Analysis of CLR-matching conditions and extinction spectra for hexagonal Die-PBFDO-Nano arrays with different refractive index of dielectric nanocylinders (n_{die} ranging from 1.20 to 1.60). The PBFDO was selected for the part of conducting polymer nanoantennas. The extinction spectra were achieved via FDTD simulations. In all panels, dielectric nanocylinders have a diameter d = 0.56 μm and a height h = 0.25 μm. The height of PBFDO layer is 0.2 μm. The periodic distance of Die-PBFDO-Nano arrays was set as r = 1.0 μm.

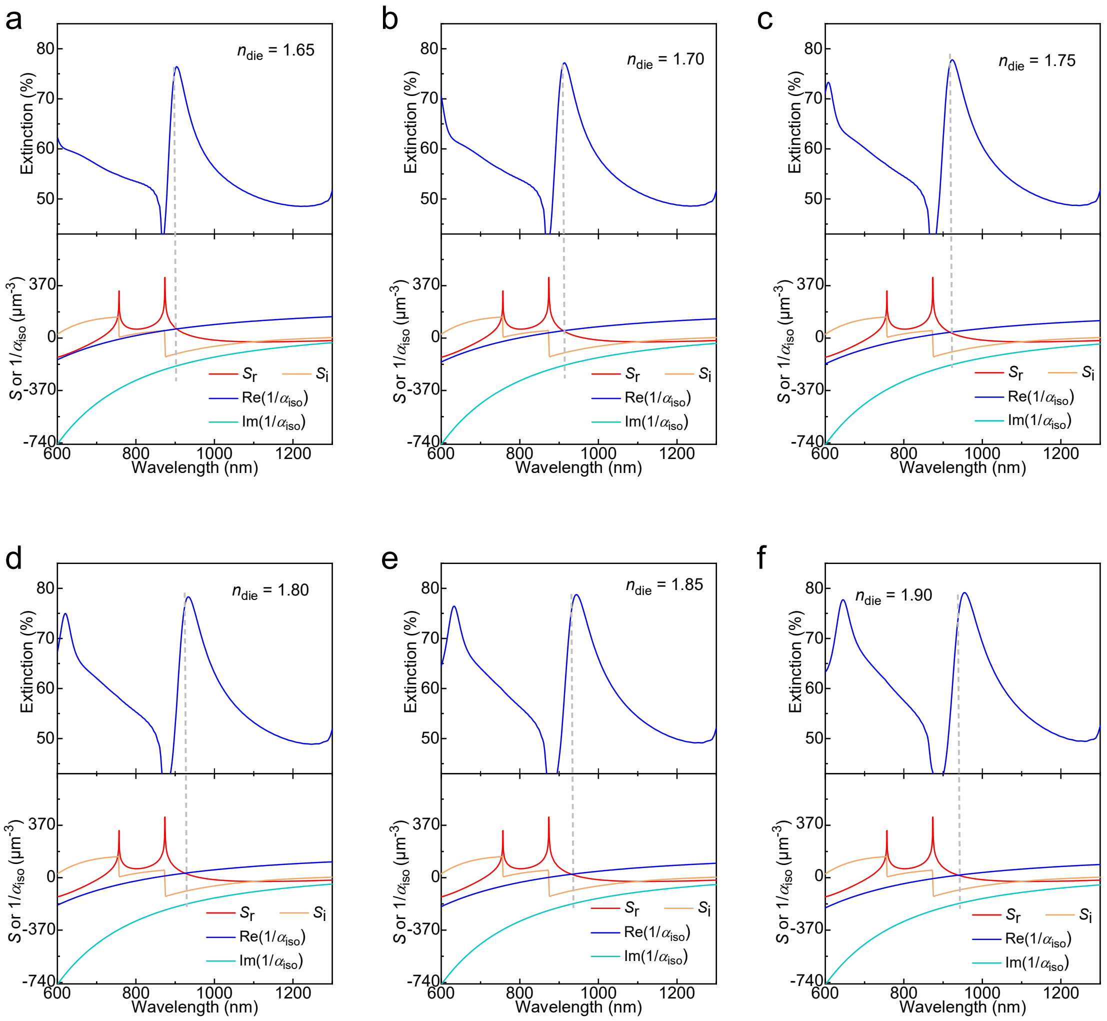

Figure S13. Analysis of CLR-matching conditions and extinction spectra for hexagonal Die-PBFDO-N arrays with different refractive index of dielectric nanocylinders (n_{die} ranging from 1.65 to 1.90). The PBFDO was selected for the part of conducting polymer nanoantennas. The extinction spectra were achieved via FDTD simulations. In all panels, dielectric nanocylinders have a diameter d = 0.56 μm and a height h = 0.25 μm. The height of PBFDO layer is 0.2 μm. The periodic distance of Die-PBFDO-Nano arrays was set as r = 1.0 μm.

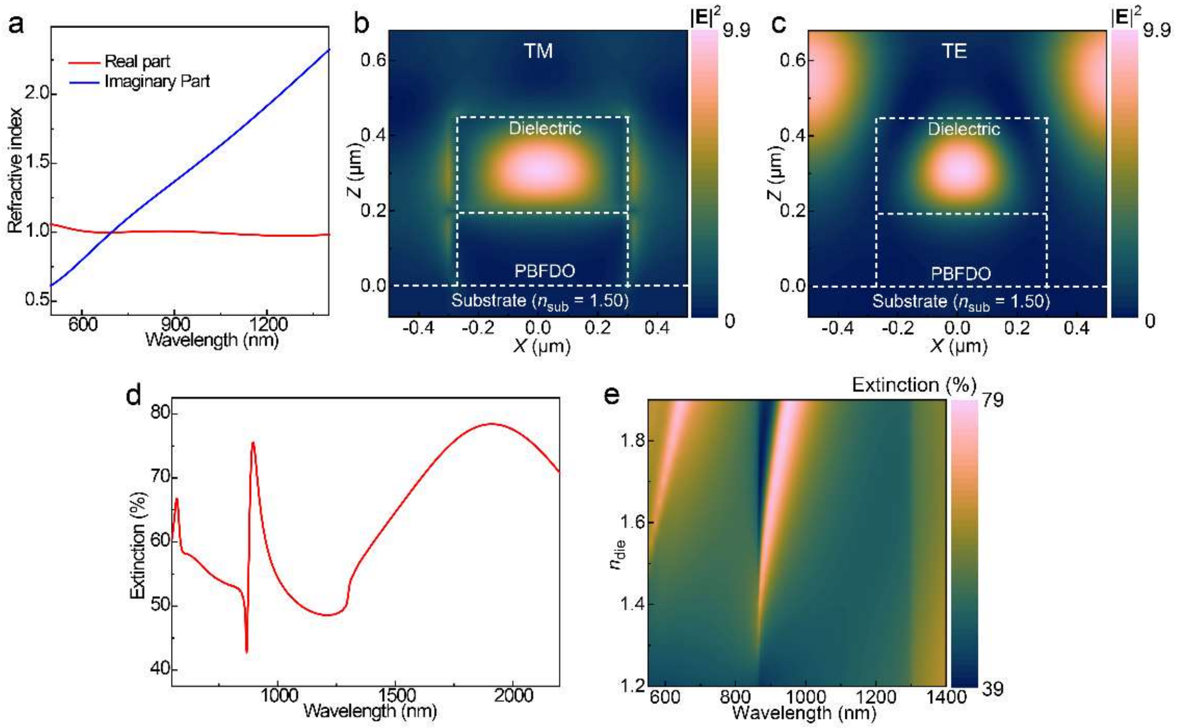

Figure S14. The effect of blocking gate from PBFDO nanoantennas. (a) Refractive index of PBFDO. The real part of refractive index is approximate to 1.0 in the wavelength region of 700-1300 nm (referred to the previous work[4]). (b) (c) TM and TE near-field distributions (near the wavelength of magnetic resonances λ_{MD}) of hexagonal arrays consisting of Die-PBFDO-Nano units, respectively. The dielectric nanocylinder has a diameter d = 0.56 μm and a height h = 0.25 μm. The height of PBFDO layer is 0.2 μm. (d) Simulated extinction spectrum of Die-PBFDO-Nano array with the periodicity r = 1.0 μm. (e) n_{die}-dependent extinction spectra of the Die-PBFDO-Nano array model. The sharp Mie resonance can be observed if $n_{die} \geq 1.45$.

Supporting Note 2: Primarily experimental demonstration of nonlocal Mie resonances (r = 1 μm)

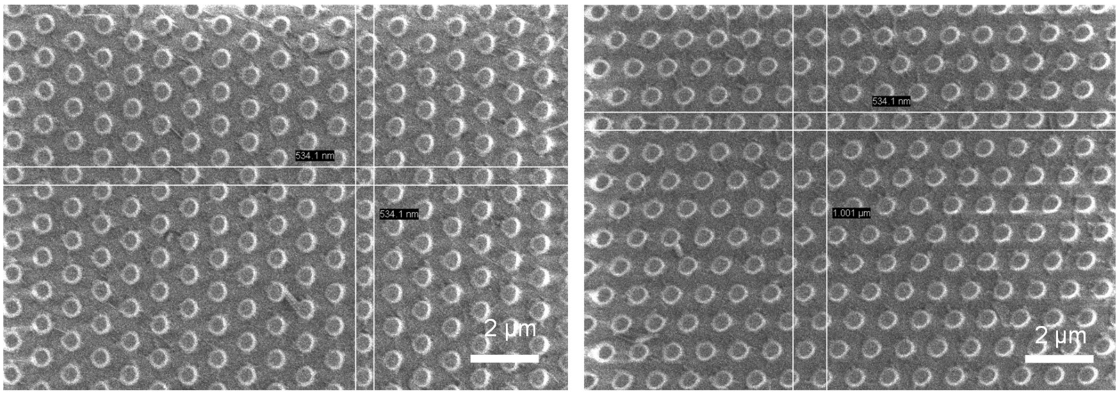

Figure S15. SEM images of Die-PEDOT-Nano arrays prepared through electron beam lithography. The hybridized nanoantenna unit has a diameter d = 0.535 μm in a hexagonal array with the periodicity r = 1.0 μm.

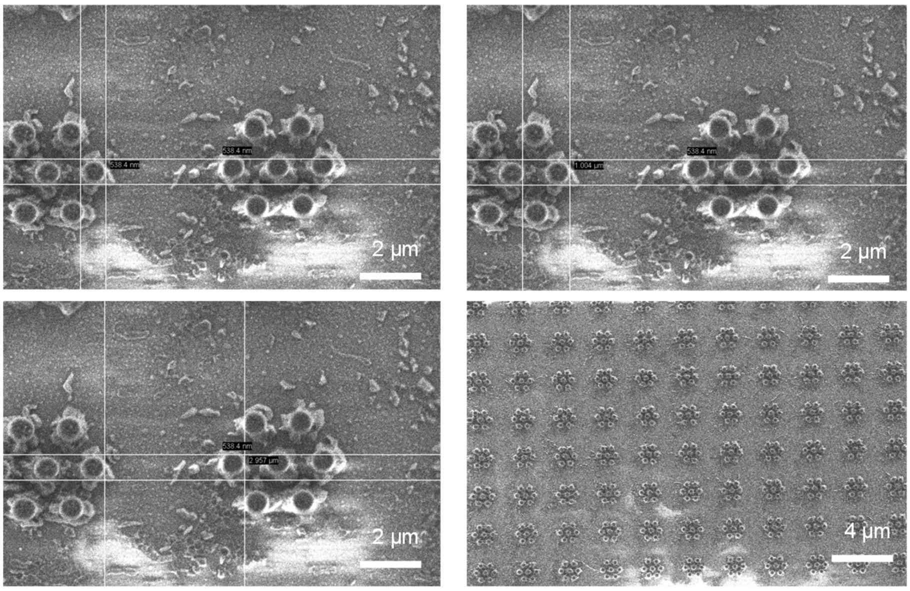

Figure S16. SEM images of Die-PEDOT-Nano arrays (N = 2) prepared through electron beam lithography. The hybridized nanoantenna unit has a diameter d = 0.538 μm in a hexagonal array with the periodicity r = 1.0 μm. To suppress the nonlocal coupling interactions between adjacent two array matrix regions, the distance between neighboring nanoantenna units belonging to two different matrix regions is enlarged to $3r$ = 3.0 μm.

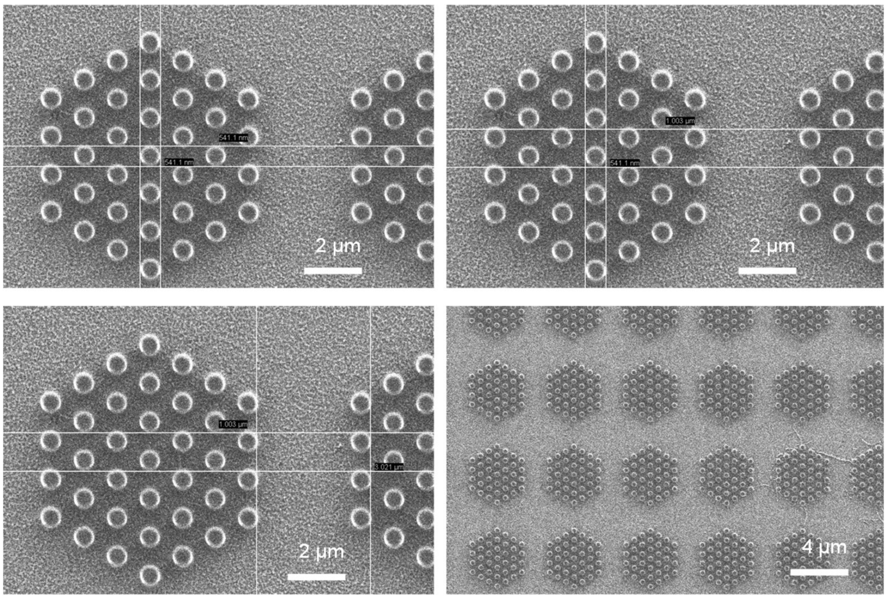

Figure S17. SEM images of Die-PEDOT-Nano arrays (N = 4) prepared through electron beam lithography. The hybridized nanoantenna unit has a diameter d = 0.54 µm in a hexagonal array with the periodicity r = 1.0 µm. To suppress the nonlocal coupling interactions between adjacent two array matrix regions, the distance between neighboring nanoantenna units belonging to two different matrix regions is enlarged to 3r = 3.0 µm.

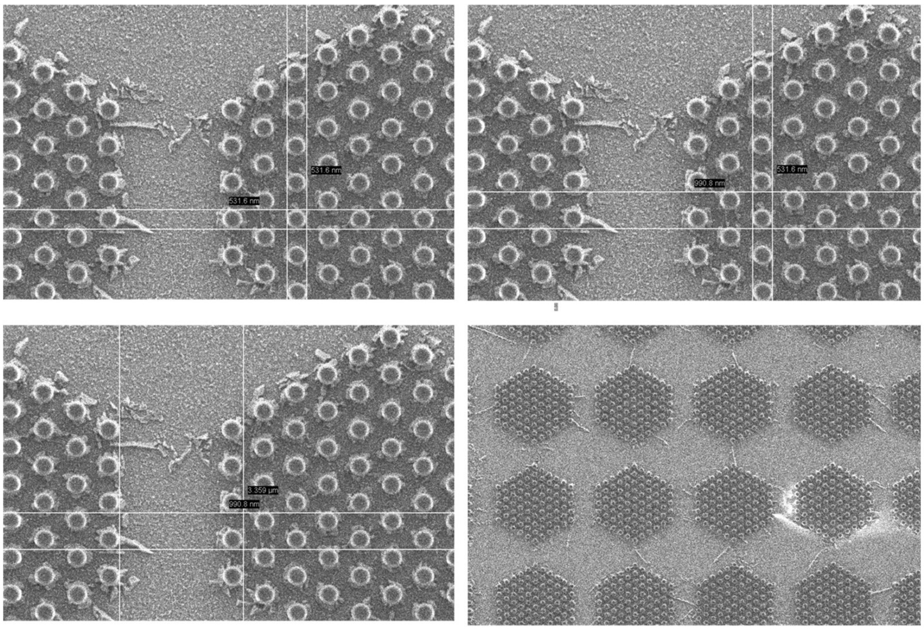

Figure S18. SEM images of Die-PEDOT-Nano arrays (N = 6) prepared through electron beam lithography. The hybridized nanoantenna unit has a diameter d = 0.531 µm in a hexagonal array with the periodicity r = 1.0 µm. To suppress the nonlocal coupling interactions between adjacent two array matrix regions, the distance between neighboring nanoantenna units belonging to two different matrix regions is enlarged to 3r = 3.0 µm.

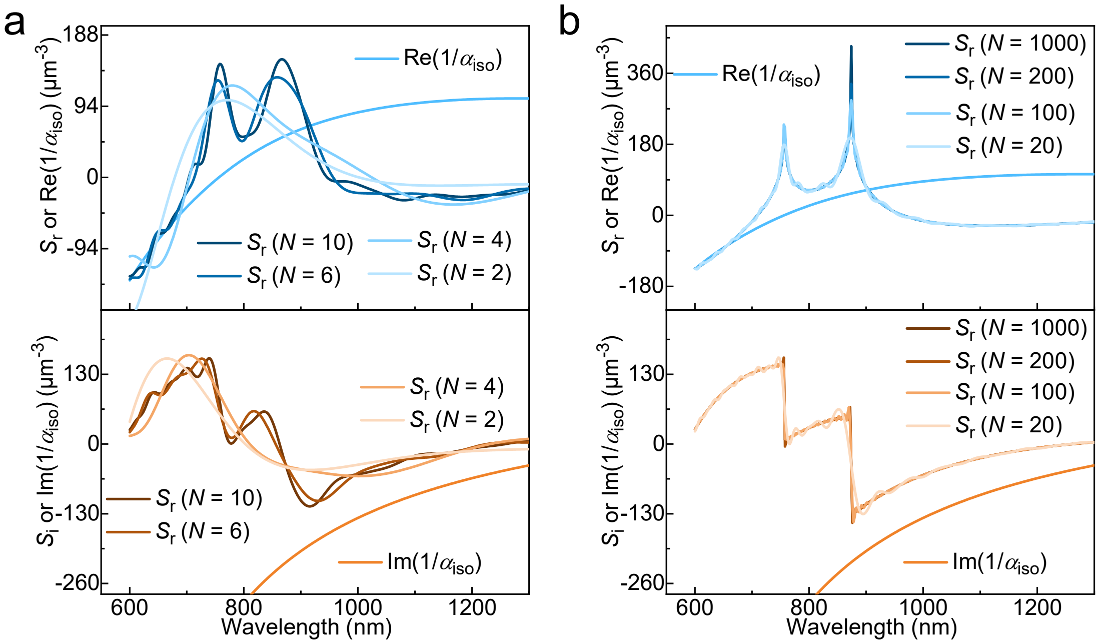

Figure S19. Array factors (*S*) dependent on the layer number (*N*) of hexagonal arrays of Die-PEDOT-Nano units. The conditions including the periodicity r = 1.0 μm and the refractive index of air n_{air} = 1.0 in wave vector were used for these calculations. (a) N ranges from 2 to 10. (b) N ranges from 20-1000.

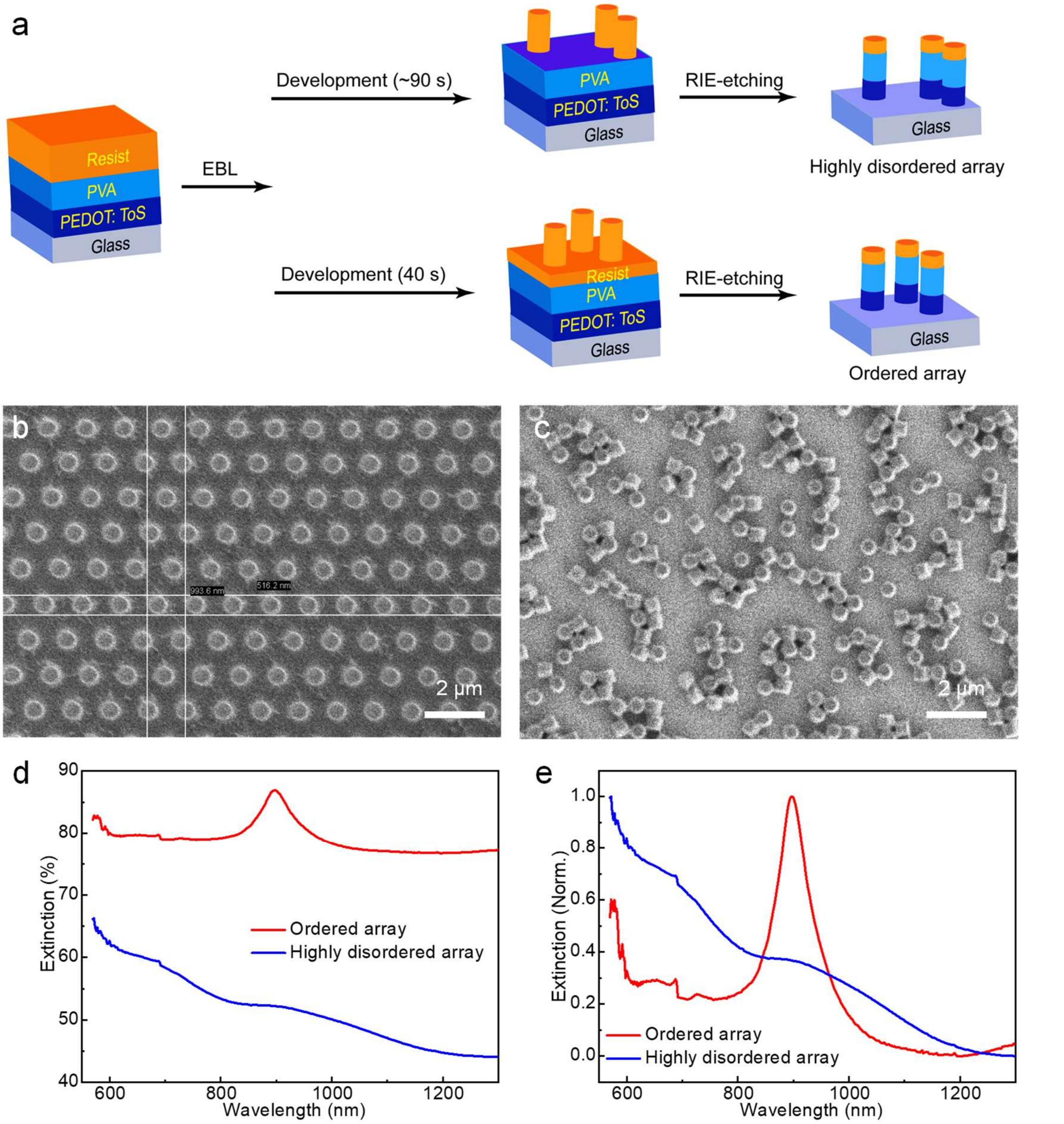

Figure S20. Extinction spectra of ordered and highly disordered arrays consisting of Die-PEDOT-Nano units. (a) Nanofabrication processes to control the ordered and highly disordered arrays. Before spin-coating negative resist, we spin-coated PVA solution (DI water as the solvent) as the control layer. After electron beam lithography (EBL) process, the short time of development (40 s) could maintain the ordered array, while elongating the development time (to 90 s) could generate the highly disordered array. (b) (c) SEM images of ordered and highly disordered array structures, respectively. The hybridized nanoantenna unit has a diameter d = 0.51 μm. For the ordered array, the periodicity is r = 1.0 μm. (d) (e) absolute and normalized extinction spectra of ordered and high disordered arrays, respectively.

Supporting Note 3: Angular dispersive relationships in hexagonal lattice

The angular relationship between the resonance wavelength (λ_r) and incident angle (θ_i) can be deduced with the wave vector (**k**), the wave vector component that is parallel to the lattice ($k_{||} = |\mathbf{k}|\sin\theta_i$), and the reciprocal lattice vector **G**, which has an equivalent relationship[5]:

$$|\mathbf{k}| = |\mathbf{G} + k_{||}| \quad \text{(S1)}$$

In a hexagonal array (referred to the previous work[2]), the equation between λ_r and periodicity (r) is:

$$\lambda_r = \sqrt[3]{\frac{2}{3}}\, n_{eff} r \quad \text{(S2)}$$

where n_{eff} is the refractive index for the environment of CLRs. Here, we used the effective periodicity r_{eff}:

$$r_{eff} = \sqrt[3]{\frac{2}{3}}\, r \quad \text{(S3)}$$

As $\sqrt[3]{\frac{2}{3}} \approx 0.874 \approx \frac{\sqrt{3}}{2}$, we used the approximate formula: $r_{eff} \approx \frac{\sqrt{3}}{2} r$

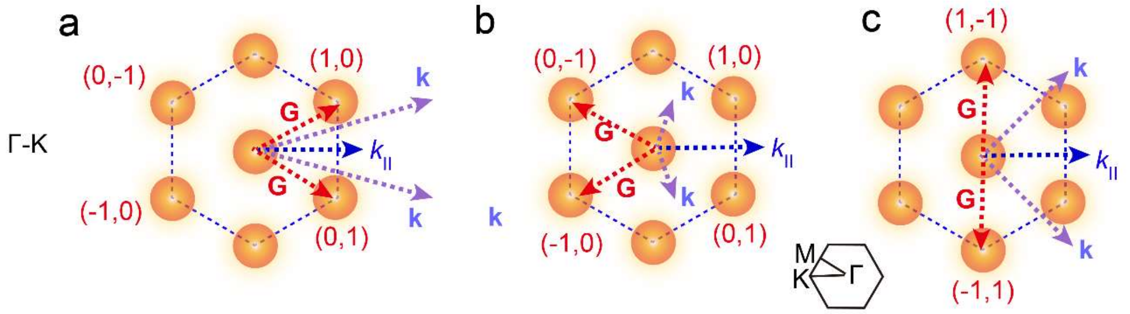

Figure S21. First diffraction orders of Rayleigh Anomalies (RA)s along the Γ-K direction of hexagonal reciprocal lattice diagrams. Here, the the Γ-K direction is parallel to the in-plane component of wave vector of incident light. **G** is the reciprocal lattice vector, **k** is the wave vector, and $k_{||}$ is the wave vector component parallel to the lattice.

In the first diffraction orders of Rayleigh Anomalies (RA)s, we used $|\mathbf{G}| = 2\pi/r_{eff}$. According to Equation S1, (1,0) (0,1) RAs in Figure S21a have an equivalent relationship:

$$\left(\frac{2\pi}{r_{eff}}\cos\frac{\pi}{3}\right)^2 + \left(\frac{2\pi}{r_{eff}}\cos\frac{\pi}{6} + \frac{2\pi}{\lambda_r}\sin\theta_i\right)^2 = \left(\frac{2\pi}{\lambda_r}\right)^2 \quad \text{(S4)}$$

Then we could achieve a quadratic equation with λ_r:

$$\lambda_r^2 + \sqrt{3}\lambda_r r_{eff}\sin\theta_i + r_{eff}^2(\sin^2\theta_i - 1) = 0 \quad \text{(S5)}$$

We solved this equation to achieve a formula among λ_r ($\lambda_r > 0$), r_{eff} and $\sin\theta_i$:

$$\lambda_r = -\frac{\sqrt{3}}{2} r_{eff}\sin\theta_i + \frac{r_{eff}}{2}\sqrt{4 - \sin^2\theta_i} \quad \text{(S6)}$$

Considering the effective refractive index n_{eff} ($n_{eff} \geq 1$) based on the Snell law, Equation S6 is transformed into:

$$\lambda_r = -\frac{\sqrt{3}}{2} r_{eff}\sin\theta_i + \frac{r_{eff}}{2}\sqrt{4n_{eff}^2 - \sin^2\theta_i} \quad \text{(S7)}$$

Here we only investigated the scope $\theta_i = 0\sim30°$, which causes $\sin^2\theta_i \leq 0.25 \ll 4n_{eff}^2$. Thus, $\sin^2\theta_i$ can be omitted in Equation S7 and λ_r has a brief formula:

$$\lambda_r \approx -\frac{\sqrt{3}}{2} r_{eff}\sin\theta_i + n_{eff} r_{eff} \quad \text{(S8)}$$

r_{eff} can be changed with r, and then the formula of λ_r is:

$$\lambda_r \approx -\frac{3}{4} r\sin\theta_i + \frac{\sqrt{3}}{2} n_{eff} r \quad \text{(S9)}$$

As (-1,0) (0,-1) RAs in Figure S21b have an opposite direction along X-axis, the Equation S4 was changed into:

$$(\frac{2\pi}{r_{\text{eff}}}\cos\frac{\pi}{3})^2 + (\frac{2\pi}{r_{\text{eff}}}\cos\frac{\pi}{6} - \frac{2\pi}{\lambda_{\text{r}}}\sin\theta_{\text{i}})^2 = (\frac{2\pi}{\lambda_{\text{r}}})^2 \qquad \text{(S10)}$$

After the similarly above process, (-1,0) (0,-1) RAs has a formula of λ_{r}:

$$\lambda_{\text{r}} \approx \frac{3}{4}r\sin\theta_{\text{i}} + \frac{\sqrt{3}}{2}n_{\text{eff}}r \qquad \text{(S11)}$$

In Figure S21c, the (-1,1) (1,-1) RAs exhibits an equation with **G** and $k_{\parallel}$:

$$(\frac{2\pi}{r_{\text{eff}}})^2 + (\frac{2\pi}{\lambda_{\text{r}}}\sin\theta_{\text{i}})^2 = (\frac{2\pi}{\lambda_{\text{r}}})^2 \qquad \text{(S12)}$$

Which gives a formula of λ_{r} (considering n_{eff} from Snell law):

$$\lambda_{\text{r}} = r_{\text{eff}}\sqrt{n_{\text{eff}}^2 - \sin^2\theta_{\text{i}}} \qquad \text{(S13)}$$

Then changing r_{eff} into r can achieve the formula of λ_{r}:

$$\lambda_{\text{r}} \approx \frac{\sqrt{3}r}{2}\sqrt{n_{\text{eff}}^2 - \sin^2\theta_{\text{i}}} \qquad \text{(S14)}$$

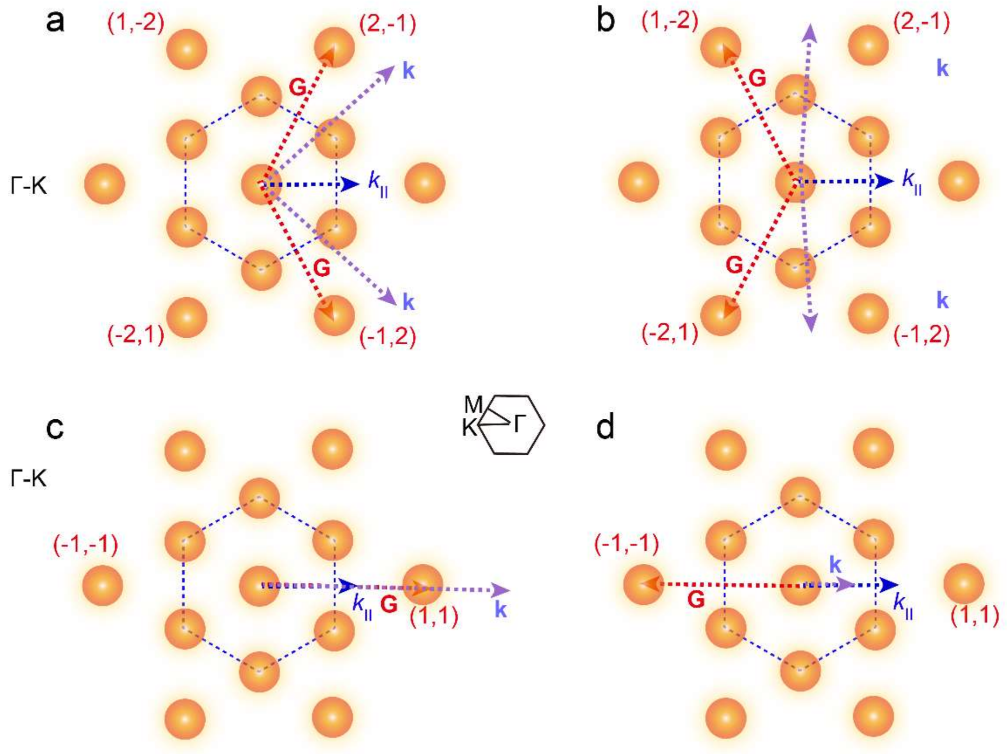

Figure S22. Second diffraction orders of Rayleigh Anomalies (RA)s along the Γ-K direction of hexagonal reciprocal lattice diagrams. Here, the the Γ-K direction is parallel to the in-plane component of wave vector of incident light. G is the reciprocal lattice vector, **k** is the wave vector, and $k_{\parallel}$ is the wave vector component parallel to the lattice.

In the second diffraction orders of Rayleigh Anomalies (RA)s, we used $|\mathbf{G}| = \sqrt{3}\frac{2\pi}{r_{\text{eff}}}$. According to Equation S1, (2,-1) (-1,2) RAs in Figure S22a have an equivalent relationship:

$$(\sqrt{3}\frac{2\pi}{r_{\text{eff}}}\cos\frac{\pi}{6})^2 + (\sqrt{3}\frac{2\pi}{r_{\text{eff}}}\cos\frac{\pi}{3} + \frac{2\pi}{\lambda_{\text{r}}}\sin\theta_{\text{i}})^2 = (\frac{2\pi}{\lambda_{\text{r}}})^2 \qquad \text{(S15)}$$

Then we achieved a quadratic equation with λ_{r}:

$$3\lambda_r^2 + \sqrt{3}\lambda_r r_{eff}\sin\theta_i + r_{eff}^2(\sin^2\theta_i - 1) = 0 \tag{S16}$$

we solved this equation to achieve a formula among λ_r ($\lambda_r > 0$), r_{eff} and $\sin\theta_i$:

$$\lambda_r = -\frac{\sqrt{3}}{6}r_{eff}\sin\theta_i + \frac{r_{eff}}{2}\sqrt{\frac{4}{3} - \sin^2\theta_i} \tag{S17}$$

Considering the effective refractive index n_{eff} ($n_{eff} \geq 1$) based on the Snell law, Equation S17 is transformed into:

$$\lambda_r = -\frac{\sqrt{3}}{6}r_{eff}\sin\theta_i + \frac{n_{eff}r}{\sqrt{3}}\sqrt{1 - \frac{3}{4n_{eff}^2}\sin^2\theta_i} \tag{S18}$$

Here we constructed the equation: $\cos\alpha = \sqrt{1 - \frac{3}{4n_{eff}^2}\sin^2\theta_i}$, and then we achieved $n_{eff}\sin\alpha = \frac{\sqrt{3}}{2}\sin\theta_i$. Then, Equation S18 is transformed into:

$$\lambda_r \approx \frac{n_{eff}}{3}r_{eff}\sin\alpha + \frac{n_{eff}r_{eff}}{\sqrt{3}}\cos\alpha = \frac{2n_{eff}}{3}r_{eff}\sin(\alpha + \frac{\pi}{3}) \tag{S19}$$

Then changing r_{eff} into r can achieve the formula of λ_r:

$$\lambda_r \approx \frac{n_{eff}}{\sqrt{3}}r\sin(\alpha + \frac{\pi}{3}) \tag{S20}$$

Similarly, the (1,-2) (-2,1) RAs in Figure S22b has a formula of λ_r

$$\lambda_r \approx \frac{n_{eff}}{\sqrt{3}}r\sin(\alpha - \frac{\pi}{3}) \tag{S21}$$

In Figure S22c and S22d, as **G** is parallel to $k_{||}$, the relationship

$$\sqrt{3}\frac{2\pi}{r_{eff}} + \frac{2\pi}{\lambda_r}\sin\theta_i = \frac{2\pi}{\lambda_r} \tag{S22}$$

for (-1,-1) RA achieves the formulas:

$$\lambda_r = \frac{r_{eff}}{\sqrt{3}}(1 - \sin\theta_i) \tag{S23}$$

,and the relationship

$$\sqrt{3}\frac{2\pi}{r_{eff}} - \frac{2\pi}{\lambda_r}\sin\theta_i = \frac{2\pi}{\lambda_r} \tag{S24}$$

for (1,1) can achieve the formula:

$$\lambda_r = \frac{r_{eff}}{\sqrt{3}}(1 + \sin\theta_i) \tag{S25}$$

Both considering the conversion by r and n_{eff}, Equation S23 and S25 is transformed into:

$$\lambda_r \approx \frac{r}{2}(n_{eff} - \sin\theta_i) \tag{S26}$$

and:

$$\lambda_r \approx \frac{r}{2}(n_{eff} + \sin\theta_i) \tag{S27}$$

, respectively.

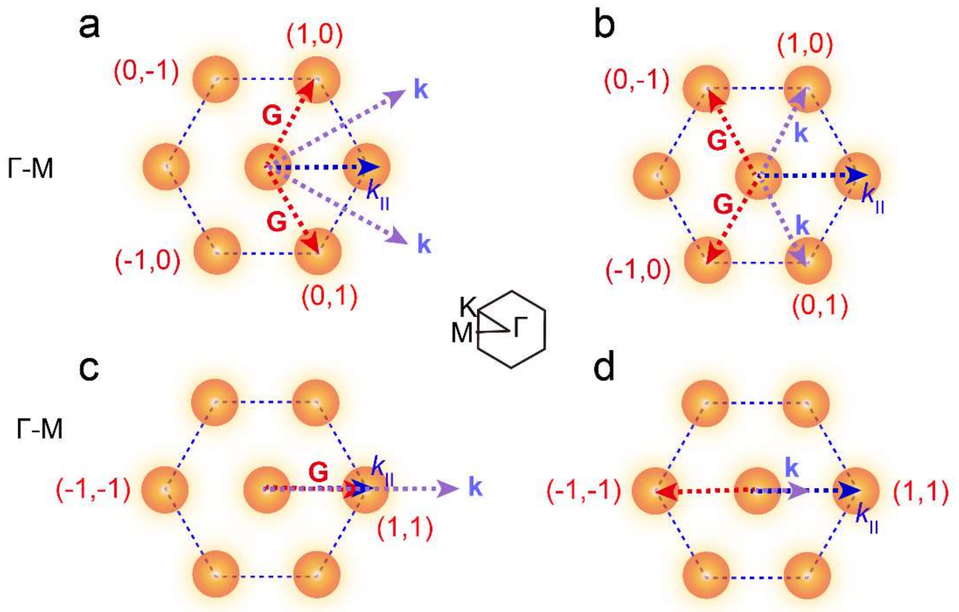

Figure S23. First diffraction orders of Rayleigh Anomalies (RA)s along the Γ-M direction of hexagonal reciprocal lattice diagrams. Here, the the Γ-M direction is parallel to the in-plane component of wave vector of incident light. G is the reciprocal lattice vector, **k** is the wave vector, and $k_{||}$ is the wave vector component parallel to the lattice.

According to Equation S1, (1,0) (0,1) RAs (belonging to the first diffraction orders) in Figure S23a have an equivalent relationship:

$$(\frac{2\pi}{r_{\mathrm{eff}}}\cos\frac{\pi}{6})^2 + (\frac{2\pi}{r_{\mathrm{eff}}}\cos\frac{\pi}{3} + \frac{2\pi}{\lambda_{\mathrm{r}}}\sin\theta_{\mathrm{i}})^2 = (\frac{2\pi}{\lambda_{\mathrm{r}}})^2 \tag{S28}$$

which achieves the formula of λ_{r} by solving a quadratic equation:

$$\lambda_{\mathrm{r}} = -\frac{1}{2}r_{\mathrm{eff}}\sin\theta_{\mathrm{i}} + \frac{\sqrt{3}r_{\mathrm{eff}}}{2}\sqrt{\frac{4}{3} - \sin^2\theta_{\mathrm{i}}} \tag{S29}$$

Considering the effective refractive index n_{eff} ($n_{\mathrm{eff}} \geq 1$), Equation S29 is transformed into:

$$\lambda_{\mathrm{r}} = -\frac{1}{2}n_{\mathrm{eff}}r_{\mathrm{eff}}\sin\theta_{\mathrm{i}} + \frac{\sqrt{3}r_{\mathrm{eff}}}{2}\sqrt{\frac{4}{3}n_{\mathrm{eff}}^2 - \sin^2\theta_{\mathrm{i}}} \tag{S30}$$

We used the equation $\cos\alpha = \sqrt{1 - \frac{3}{4n_{\mathrm{eff}}^2}\sin^2\theta_{\mathrm{i}}}$, in these case:

$$n_{\mathrm{eff}}\sin\alpha = \frac{\sqrt{3}}{2}\sin\theta_{\mathrm{i}} \tag{S31}$$

and we introduced the above equations into Equation S30:

$$\lambda_{\mathrm{r}} = -\frac{1}{\sqrt{3}}r_{\mathrm{eff}}n_{\mathrm{eff}}\sin\alpha + n_{\mathrm{eff}}r_{\mathrm{eff}}\cos\alpha = \frac{2}{\sqrt{3}}n_{\mathrm{eff}}r_{\mathrm{eff}}\sin(\frac{\pi}{3} - \alpha) \tag{S32}$$

Then we changed the α to θ_{i}:

$$\lambda_{\mathrm{r}} = n_{\mathrm{eff}}r\sin(\frac{\pi}{3} - \theta_{\mathrm{i}}) \tag{S33}$$

For the (0, -1) (-1, 0) RAs in Figure S23b, there is a relationship with λ_{r}:

$$(\frac{2\pi}{r_{\mathrm{eff}}}\cos\frac{\pi}{6})^2 + (\frac{2\pi}{r_{\mathrm{eff}}}\cos\frac{\pi}{3} - \frac{2\pi}{\lambda_{\mathrm{r}}}\sin\theta_{\mathrm{i}})^2 = (\frac{2\pi}{\lambda_{\mathrm{r}}})^2 \tag{S34}$$

and it can be solved:

$$\lambda_{\mathrm{r}} = \frac{1}{2}r_{\mathrm{eff}}\sin\theta_{\mathrm{i}} + \frac{\sqrt{3}r_{\mathrm{eff}}}{2}\sqrt{\frac{4}{3} - \sin^2\theta_{\mathrm{i}}} \tag{S35}$$

By using the same processes, the simplified formula of λ_{r} (if n_{eff} equals 1) is:

$$\lambda_{\mathrm{r}} = n_{\mathrm{eff}}r\sin(\frac{\pi}{3} + \theta_{\mathrm{i}}) \tag{S36}$$

For (1,1) RAs in Figure S23c, there is a simple relationship along *X*-axis:

$$\frac{2\pi}{r_{\text{eff}}} + \frac{2\pi}{\lambda_{\text{r}}}\sin\theta_{\text{i}} = \frac{2\pi}{\lambda_{\text{r}}} \quad \text{(S37)}$$

and then we use r_{eff} from Equation S3 to achieve:

$$\lambda_{\text{r}} \approx \frac{\sqrt{3}r}{2}(n_{\text{eff}} - \sin\theta_{\text{i}}) \quad \text{(S38)}$$

Similarly, the relationship of (-1, -1) RAs in Figure S23d:

$$\frac{2\pi}{r_{\text{eff}}} - \frac{2\pi}{\lambda_{\text{r}}}\sin\theta_{\text{i}} = \frac{2\pi}{\lambda_{\text{r}}} \quad \text{(S39)}$$

can transform a formula:

$$\lambda_{\text{r}} \approx \frac{\sqrt{3}r}{2}(n_{\text{eff}} + \sin\theta_{\text{i}}) \quad \text{(S40)}$$

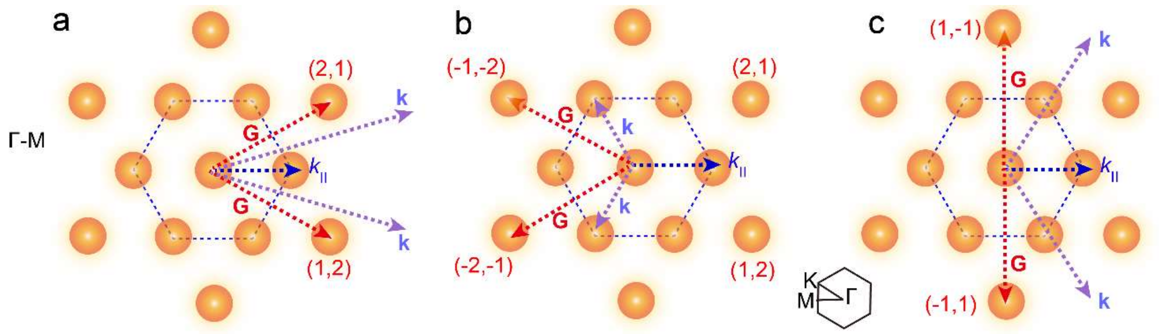

Figure S24. Second diffraction orders of Rayleigh Anomalies (RA)s along the Γ-M direction of hexagonal reciprocal lattice diagrams. Here, the the Γ-K direction is parallel to the in-plane component of wave vector of incident light. G is the reciprocal lattice vector, **k** is the wave vector, and $k_{||}$ is the wave vector component parallel to the lattice.

According to Equation S1, (2,1) (1,2) RAs in Figure S24a have an equivalent relationship:

$$\left(\sqrt{3}\frac{2\pi}{r_{\text{eff}}}\cos\frac{\pi}{3}\right)^2 + \left(\sqrt{3}\frac{2\pi}{r_{\text{eff}}}\cos\frac{\pi}{6} + \frac{2\pi}{\lambda_{\text{r}}}\sin\theta_{\text{i}}\right)^2 = \left(\frac{2\pi}{\lambda_{\text{r}}}\right)^2 \quad \text{(S41)}$$

which generates a quadratic equation with λ_{r}:

$$3\lambda_{\text{r}}^2 + 3\lambda_{\text{r}}r_{\text{eff}}\sin\theta_{\text{i}} + r_{\text{eff}}^2(\sin^2\theta_{\text{i}} - 1) = 0 \quad \text{(S42)}$$

We solve this equation to achieve a formula among λ_{r} ($\lambda_{\text{r}} > 0$), r_{eff} and $\sin\theta_{\text{i}}$:

$$\lambda_{\text{r}} = -\frac{1}{2}r_{\text{eff}}\sin\theta_{\text{i}} + \frac{\sqrt{3}r_{\text{eff}}}{6}\sqrt{4n_{\text{eff}}^2 - \sin^2\theta_{\text{i}}} \quad \text{(S43)}$$

As $\sin^2\theta_{\text{i}} \leq 0.25 \ll 4n_{\text{eff}}^2$, we omit $\sin^2\theta_{\text{i}}$ in Equation S43:

$$\lambda_{\text{r}} \approx -\frac{1}{2}r_{\text{eff}}\sin\theta_{\text{i}} + \frac{\sqrt{3}r_{\text{eff}}n_{\text{eff}}}{3} \quad \text{(S44)}$$

and use r_{eff} to achieve the final formula:

$$\lambda_{\text{r}} \approx -\frac{\sqrt{3}}{4}r\sin\theta_{\text{i}} + \frac{rn_{\text{eff}}}{2} \quad \text{(S45)}$$

Similarly, (-2,-1) (-1,-2) RAs in Figure S24b have an equivalent relationship:

$$\left(\sqrt{3}\frac{2\pi}{r_{\text{eff}}}\cos\frac{\pi}{3}\right)^2 + \left(\sqrt{3}\frac{2\pi}{r_{\text{eff}}}\cos\frac{\pi}{6} - \frac{2\pi}{\lambda_{\text{r}}}\sin\theta_{\text{i}}\right)^2 = \left(\frac{2\pi}{\lambda_{\text{r}}}\right)^2 \quad \text{(S46)}$$

and we achieve a simplified formula of λ_{r} through the sampe processes:

$$\lambda_{\text{r}} \approx \frac{\sqrt{3}}{4}r\sin\theta_{\text{i}} + \frac{rn_{\text{eff}}}{2} \quad \text{(S47)}$$

(1,-1) (-1,1) RAs in Figure S24c have a relationship with $k_{||}$:

$$\left(\sqrt{3}\frac{2\pi}{r_{\text{eff}}}\right)^2 + \left(\frac{2\pi}{\lambda_{\text{r}}}\sin\theta_{\text{i}}\right)^2 = \left(\frac{2\pi}{\lambda_{\text{r}}}\right)^2 \quad \text{(S48)}$$

which can be transformed into:

$$\lambda_r = \frac{\sqrt{3}}{3} r_{eff} \sqrt{1 - \sin^2 \theta_i} \tag{S49}$$

Further considering n_{eff} and r, this equation is:

$$\lambda_r \approx \frac{1}{2} r \sqrt{n_{eff}^2 - \sin^2 \theta_i} \tag{S50}$$

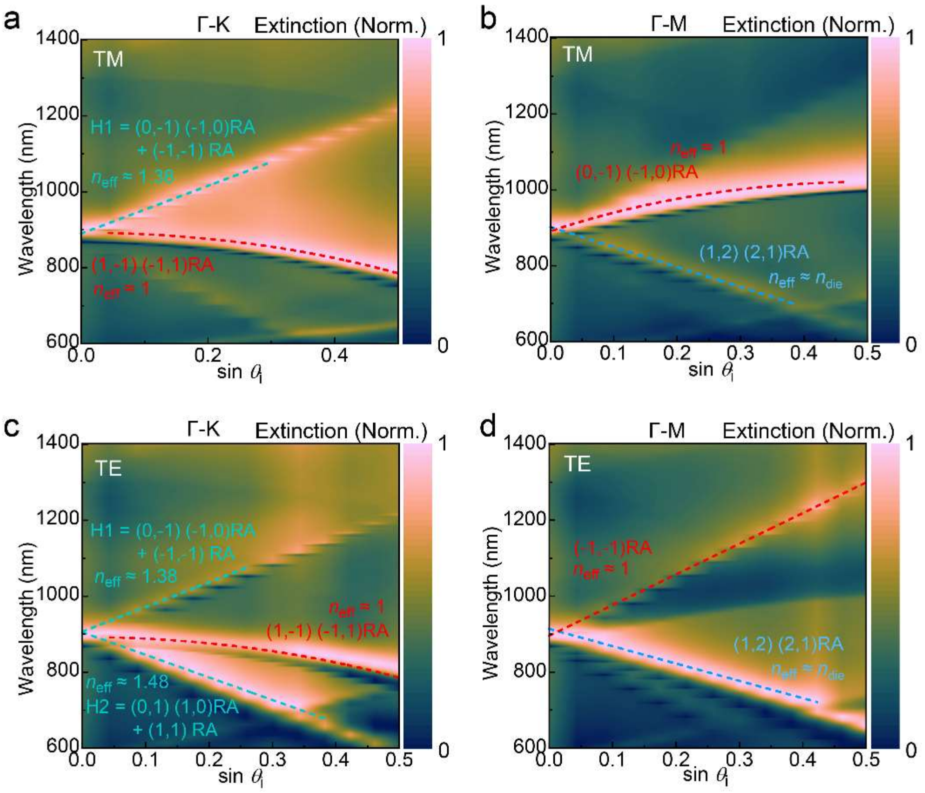

Figure S25. Angle-dependent extinction spectra (simulation results) of the Die-PEDOT-Nano array (r = 1.0 μm and d = 0.54 μm). (a) (b) (c) (d) are in $\Gamma-M_{TM}$, $\Gamma-M_{TE}$, $\Gamma-K_{TM}$ and $\Gamma-K_{TE}$ modes, respectively. The height of PEDOT nanoantennas and dielectric nanocylinders are 0.2 and 0.25 μm, respectively. In H1 and H2 modes along the Γ-K direction, we calculated that the slope of linear angular equation is 0.6~0.65r, corresponding to the complex of first (slope = 0.75r) and second diffraction orders (slope = 0.5r) of RAs.

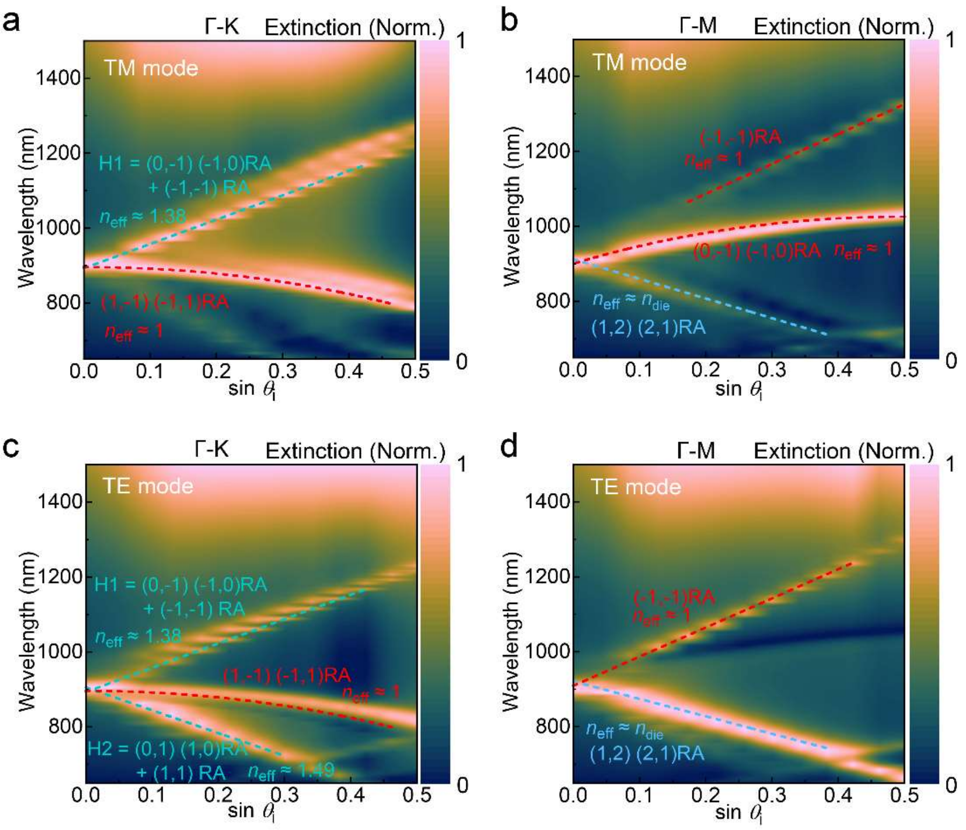

Figure S26. Angle-dependent extinction spectra (simulation results) of the Die-PEDOT-Nano array without substrate (r = 1.0 μm and d = 0.54 μm). (a) (b) (c) (d) are in Γ−M_{TM}, Γ−M_{TE}, Γ−K_{TM} and Γ−K_{TE} modes, respectively. The height of PEDOT nanoantennas and dielectric nanocylinders are 0.2 and 0.25 μm, respectively. In H1 and H2 modes along the Γ-K direction, we calculated that the slope of linear angular equation is 0.6~0.65r, corresponding to the complex of first (slope = 0.75r) and second diffraction orders (slope = 0.5r) of RAs.

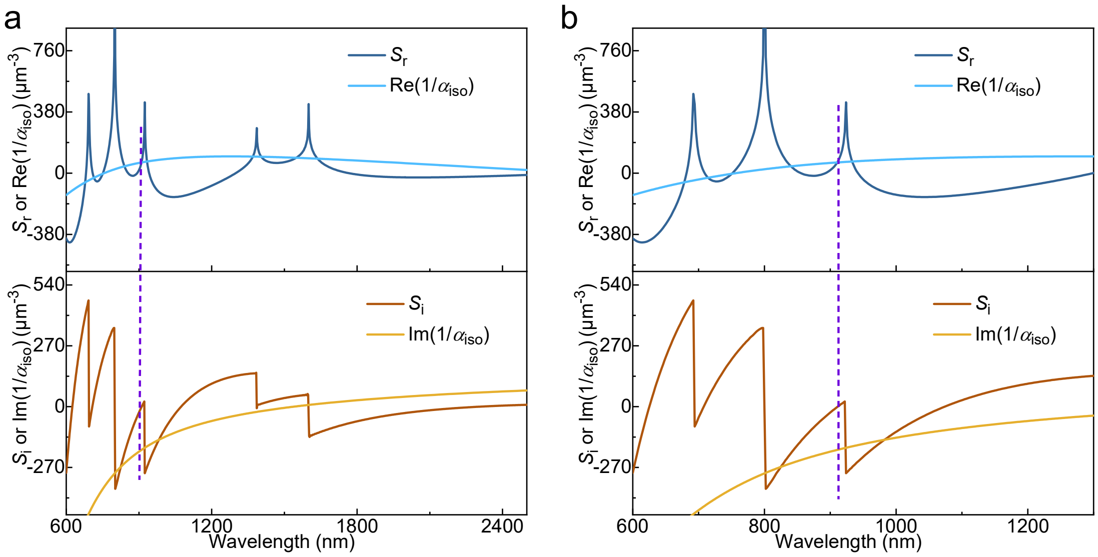

Figure S27. Analysis of second-order CLR-matching conditions for hexagonal Die-PEDOT-Nano arrays (n_{die} = 1.60). (a) (b) Full spectrum and specific spectrum scope (600-1300 nm) for the S-1/α_{iso} relationship. The acid-treated PEDOT:ToS was selected for the part of conducting polymer nanoantennas. The dielectric nanocylinders have a diameter d = 0.56 μm and a height h = 0.25 μm. The height of PEDOT layer is 0.2 μm. The periodic distance of

Die-PEDOT-Nano arrays was set as r = 1.0 μm. In the equation: $S = \sum_j^N \exp(i\mathbf{k}r_j)\left[\frac{(1-i\mathbf{k}r_j)(3\cos^2\theta_j - 1)}{r_j^3} + \frac{\mathbf{k}^2\sin^2\theta_j}{r_j}\right]$, here we used $n_{eff} = n_{die}$ = 1.60 in the calculation of the wave vector $|\mathbf{k}| = 2\pi n_{eff}/\lambda$. In (b), although the real part can reaches the equation $S_r = \mathrm{Re}(1/\alpha_{iso})$ around at 910 nm, here $S_i \approx 0$ is also observed at this cross point, implying that there is almost no suppression of damping relaxation at 910 nm. Thus, the periodic polarizability cannot be enhanced by S.

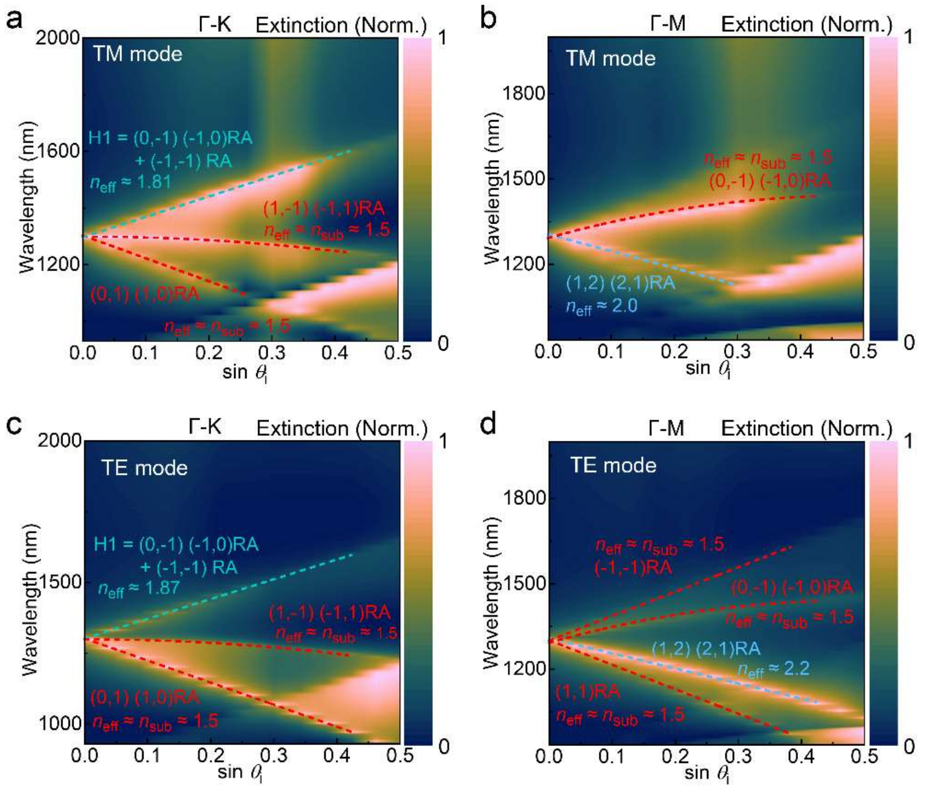

Figure S28. Angle-dependent extinction spectra (simulation results) of the Die-Nano array (r = 1.0 μm and d = 0.54 μm) with high n_{die} = 2.2. (a) (b) (c) (d) are in Γ−M$_{TM}$, Γ−M$_{TE}$, Γ−K$_{TM}$ and Γ−K$_{TE}$ modes, respectively. The refractive index of the substrate was set as n_{sub} = 1.5. In H1 along the Γ-K direction, we calculated that the slope of linear angular equation is 0.68~0.7r, which is intermediate between the first (slope = 0.75r) and second diffraction orders (slope = 0.5r) of RAs. Meanwhile, the n_{eff} is also intermediate bwtween n_{sub} and n_{die}. Along the Γ-M direction, the calculated n_{eff} in the second diffraction orders of (1,2) (2,1) RAs ranges from 2.0 to 2.2, approximating n_{die} = 2.2. These results confirm that the second diffraction orders of RAs mainly occurs in the dielectric nanocylinders.

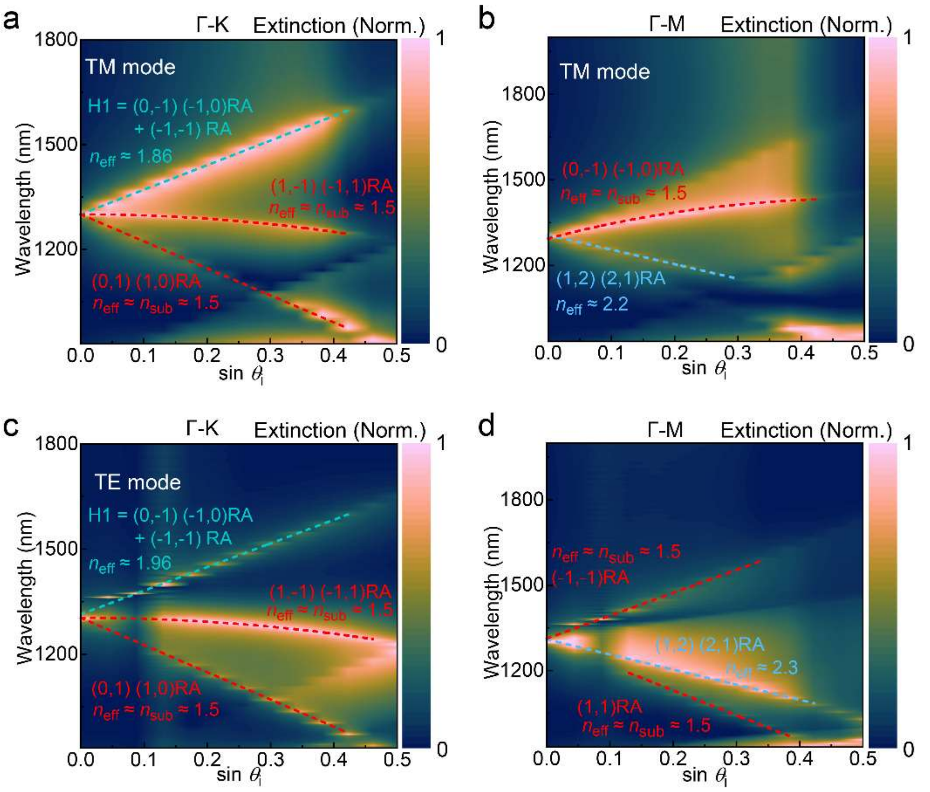

Figure S29. Angle-dependent extinction spectra (simulation results) of the Die- Nano array (r = 1.0 µm and d = 0.54 µm) with high n_{die} = 2.4. (a) (b) (c) (d) are in Γ−M$_{TM}$, Γ−M$_{TE}$, Γ−K$_{TM}$ and Γ−K$_{TE}$ modes, respectively. The refractive index of the substrate was set as n_{sub} = 1.5. In H1 along the Γ-K direction, we calculated that the slope of linear angular equation is 0.65~0.7r, which is intermediate between the first (slope = 0.75r) and second diffraction orders (slope = 0.5r) of RAs. Meanwhile, the n_{eff} is also intermediate bwtween n_{sub} and n_{die}. Along the Γ-M direction, the calculated n_{eff} in the second diffraction orders of (1,2) (2,1) RAs ranges from 2.2 to 2.4, approximating n_{die} = 2.4. These results confirm that the second diffraction orders of RAs mainly occurs in the dielectric nanocylinders.

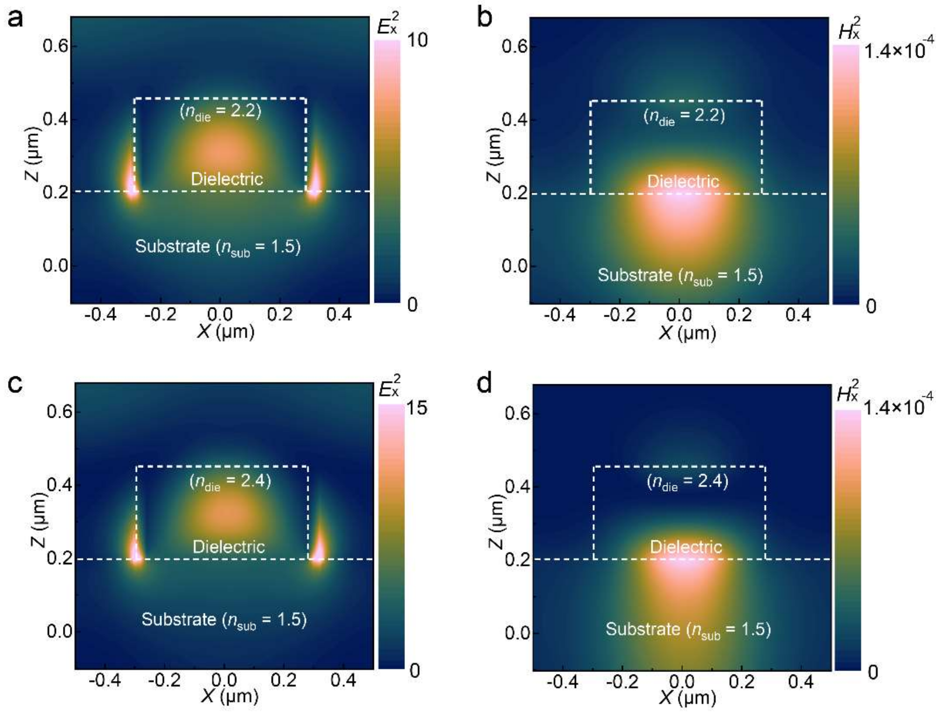

Figure S30. Near-field distribution of Die-Nano in air. (a) (b) Intensity of electric (E_x^2, in TM mode) and magnetic field (H_x^2, in TE mode) along X-axis, respectively, for n_{die} = 2.2. (c) (d) Intensity of electric (E_x^2, in TM mode) and magnetic field (H_x^2, in TE mode) along X-axis, respectively, for n_{die} = 2.4. The near-field distribution is based on the resonance wavelength $\lambda_r \approx 1300$ nm, which is approximate to $\lambda_r \approx \sqrt[3]{2/3}n_{sub}r$. The refractive index of substrate was set as n_{sub} = 1.5. In (a) and (c), the directions of wave vector, electric vector and magnetic vector of incident light are along Z-axis, X-axis and Y-axis, respectively. In (b) and (d), the directions of wave vector, electric vector and magnetic vector of incident light are along Z-axis, Y-axis and X-axis, respectively. Here we use E_x and H_x rather than $|\mathbf{E}|$ and $|\mathbf{H}|$, because the electric and magnetic fields along Z-axis (E_z and H_z, respectively), that are distributed along the interface between dielectric nanocylinders and the substrate, are also strong enough to interfere the observation of light confinement.

Supporting Note 4: Demonstrations of nonlocal Mie resonance under other periodicities

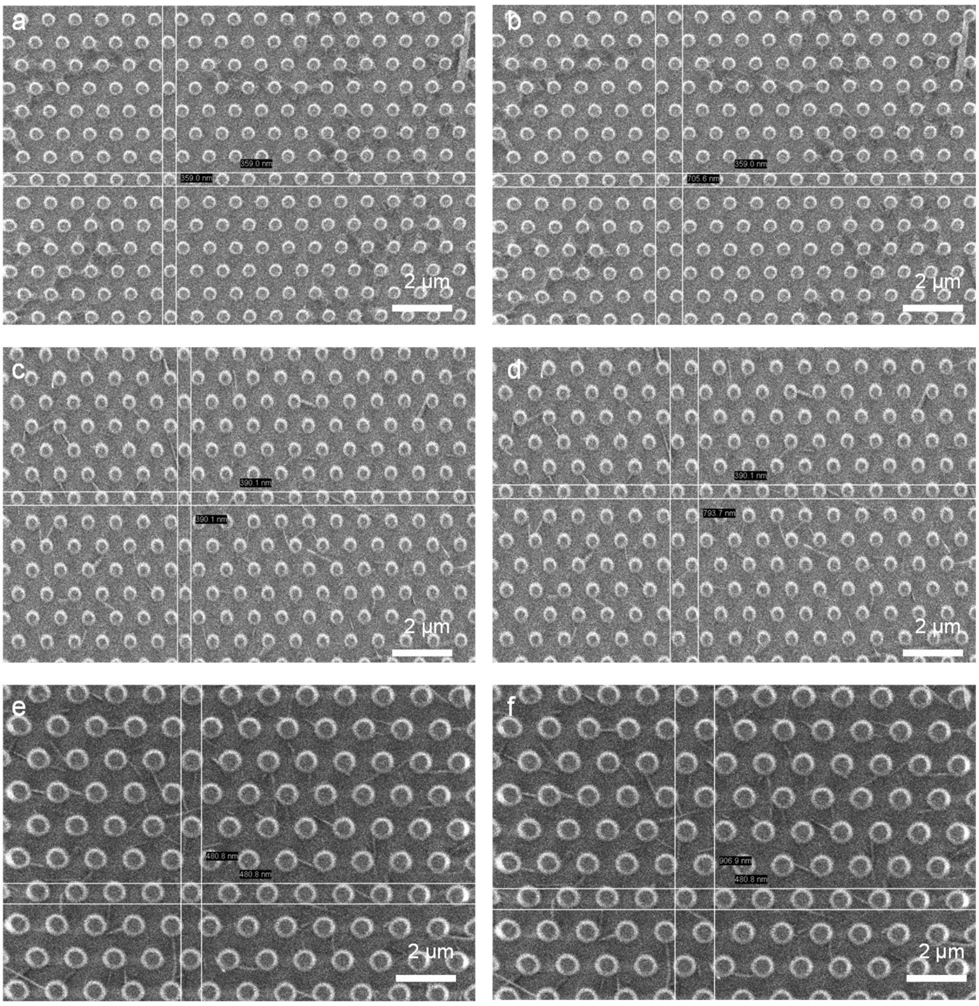

Figure S31. SEM images of Die-PEDOT-Nano arrays prepared through electron beam lithography (hexagonal shapes, *r* = 0.7-0.9 µm**).** The hybridized nanoantenna units have diameters d = 0.36, 0.39 and 0.48 µm in hexagonal arrays with the periodicity r = 0.7 (in [a] and [b]), 0.8 (in [c] and [d]) and 0.9 µm (in [e] and [f]), respectively.

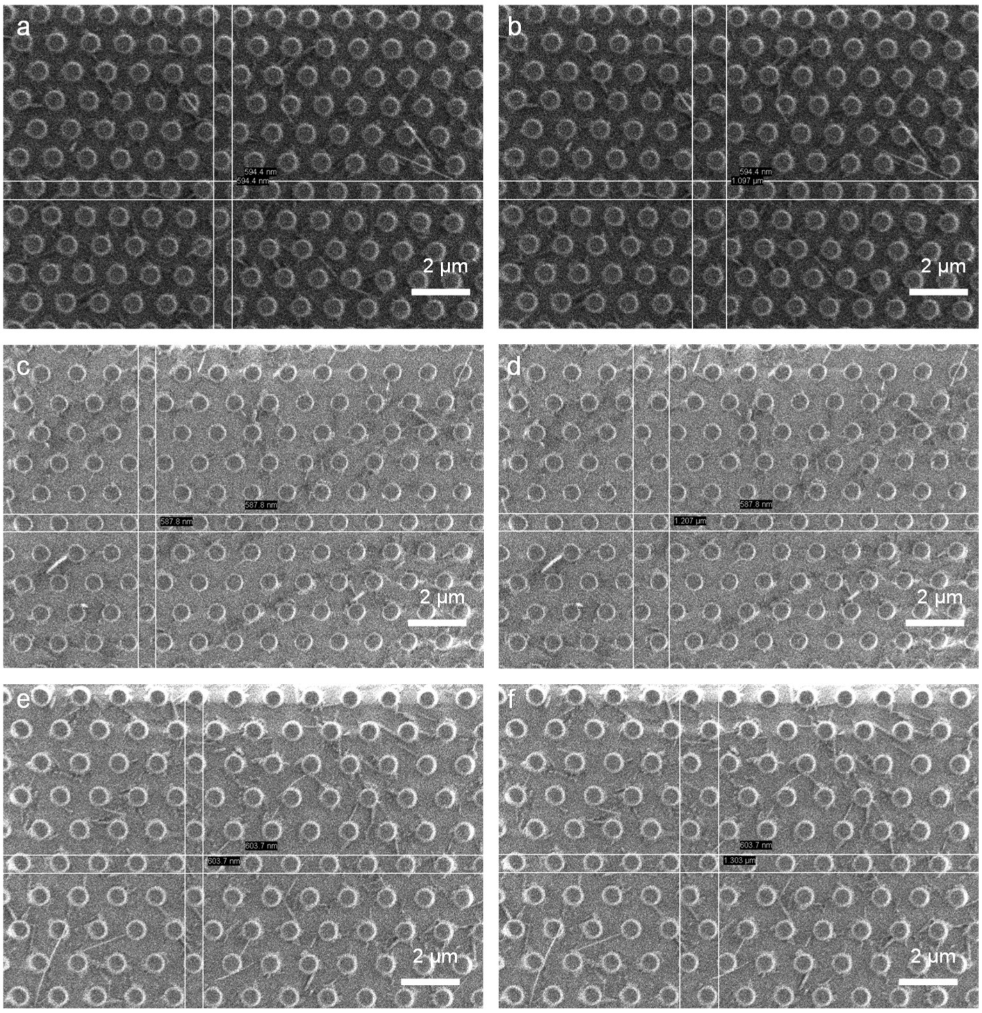

Figure S32. SEM images of Die-EDOT-Nano arrays prepared through electron beam lithography (hexagonal shapes, *r* = 1.1-1.3 μm**).** The hybridized nanoantennas have diameters *d* = 0.595, 0.59 and 0.6 μm in a hexagonal array with the periodicity *r* = 1.1 (in [a] and [b]), 1.2 (in [c] and [d]) and 1.3 μm (in [e] and [f]), respectively.

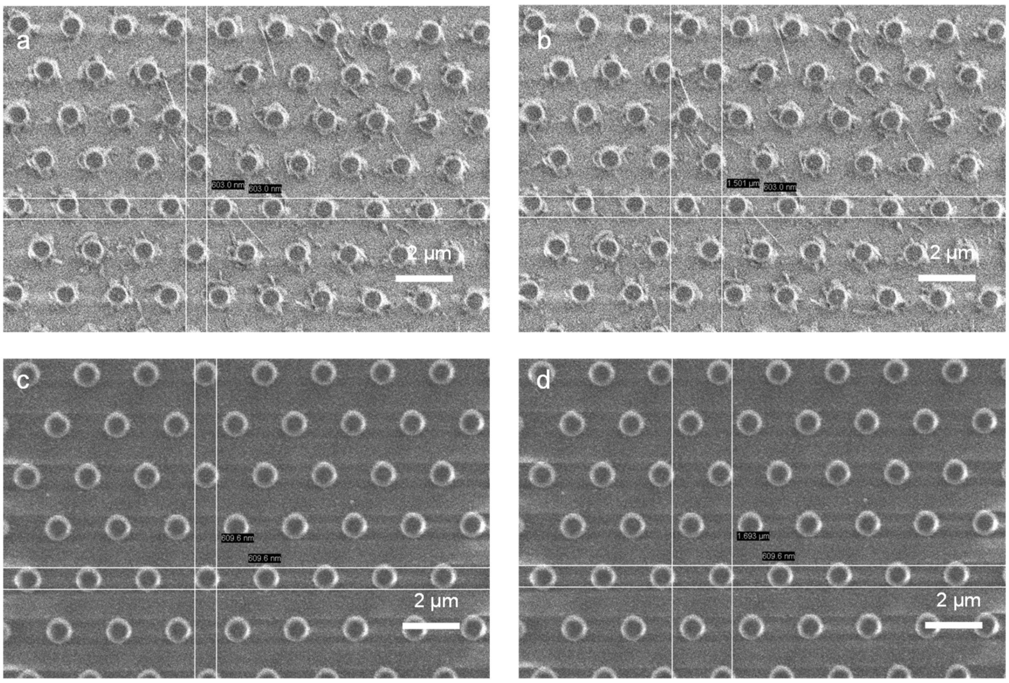

Figure S33. SEM images of Die-EDOT-Nano arrays prepared through electron beam lithography (hexagonal shapes, *r* = 1.5 and 1.7 µm). The hybridized nanoantennas have diameters *d* = 0.6 and 0.61 µm in a hexagonal array with the periodicity *r* = 1.5 (in [a] and [b]) and 1.7 µm (in [c] and [d]), respectively.

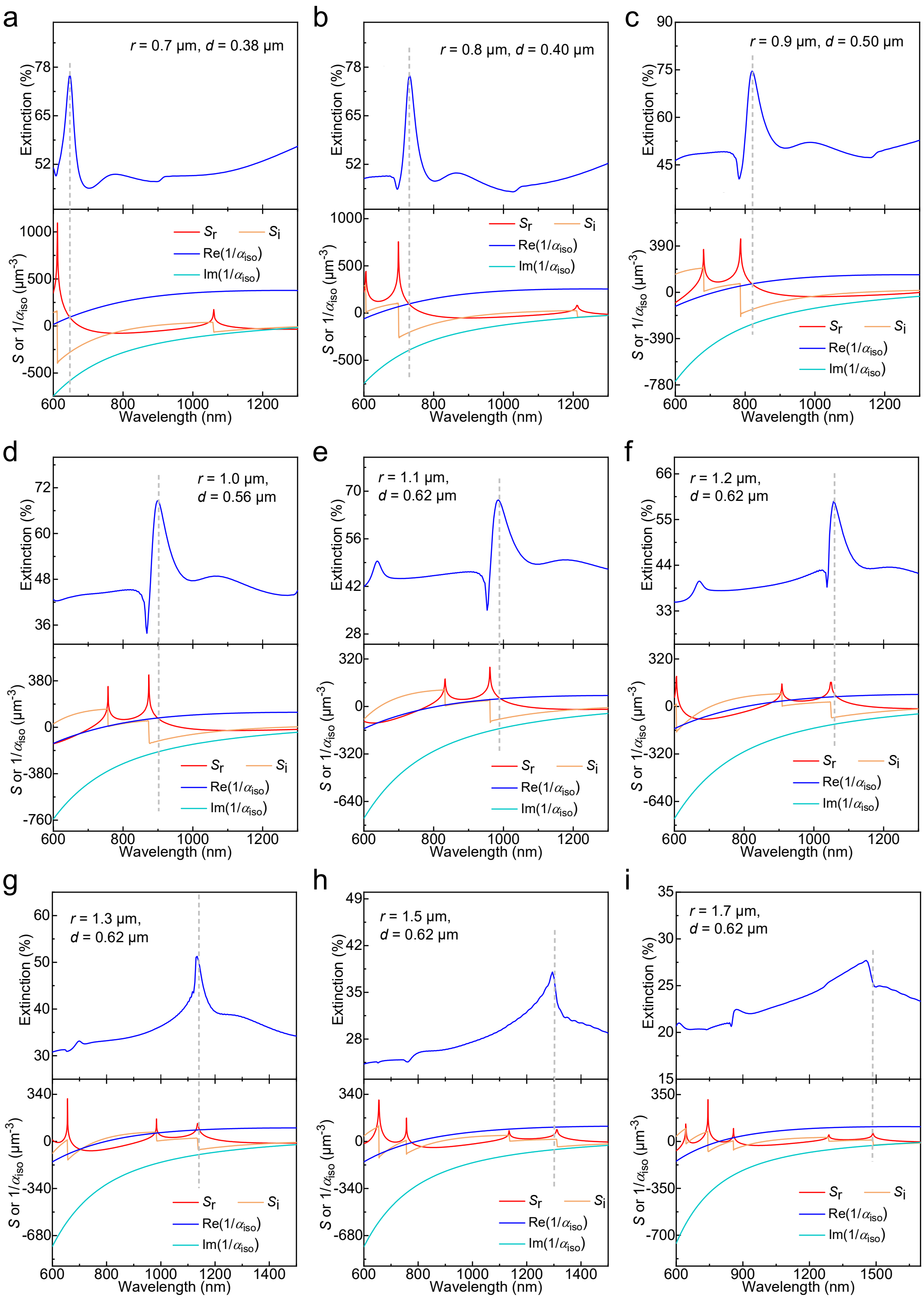

Figure S34. Analysis of CLR-matching conditions and extinction spectra for hexagonal Die-PEDOT-Nano arrays with different *r*. The acid-treated PEDOT:ToS was selected for the part of conducting polymer nanoantennas. The extinction spectra were achieved via FDTD simulations. In all panels, dielectric nanocylinders (n_{die} = 1.60) have a diameter d = 0.56 μm and a height h = 0.25 μm. The height of PEDOT layer is 0.2 μm.

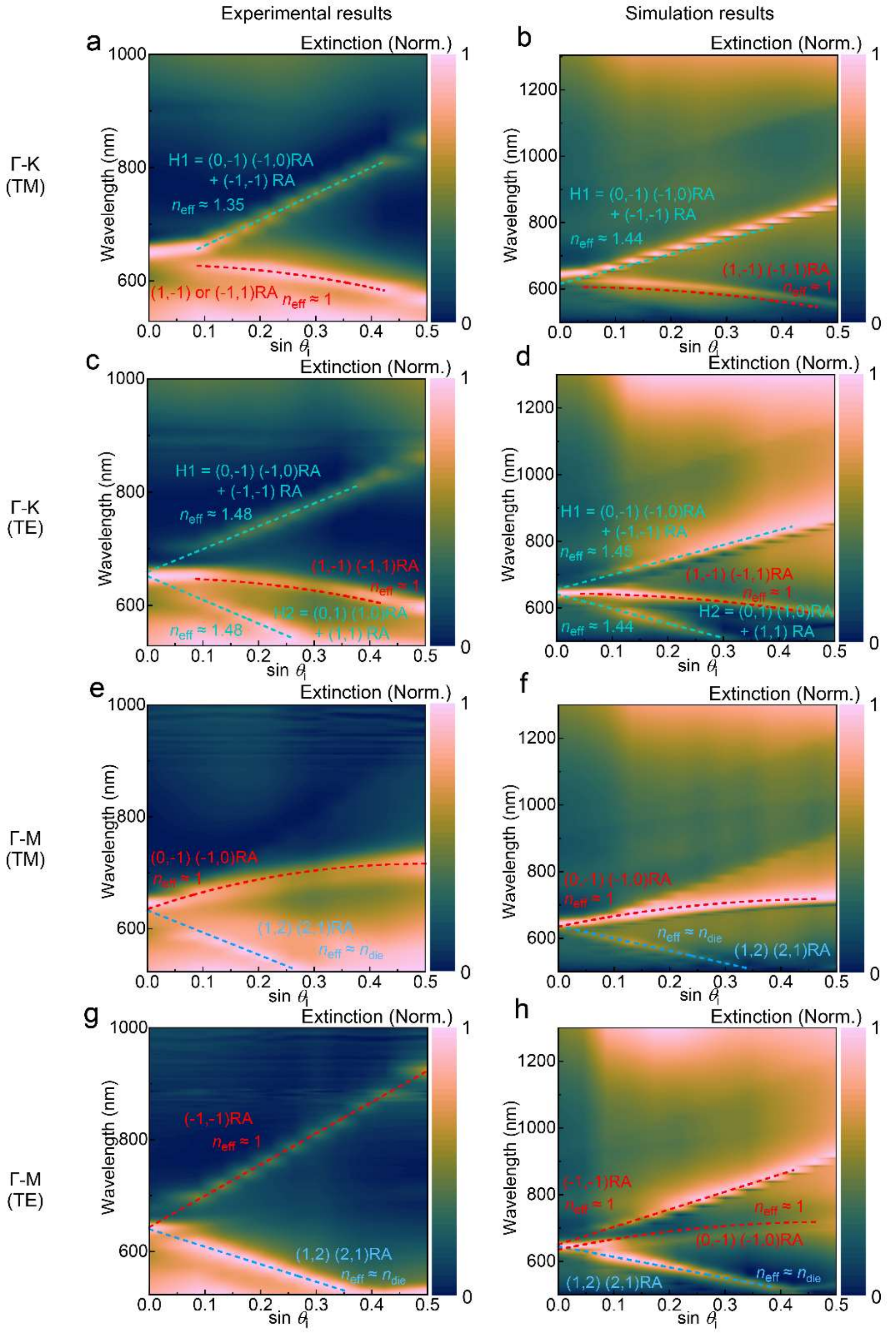

Figure S35. Angle-dependent extinction spectra of the hexagonal Die-PEDOT-Nano array (r = 0.7 µm). (a) (c) (e) (g) are experimental results in $\Gamma-M_{TM}$, $\Gamma-M_{TE}$, $\Gamma-K_{TM}$ and $\Gamma-K_{TE}$ modes, respectively. The diameter of the hybridized nanoantenna unit is 0.36 µm. (b) (d) (f) (h) are simulation results in $\Gamma-M_{TM}$, $\Gamma-M_{TE}$, $\Gamma-K_{TM}$ and $\Gamma-K_{TE}$ modes, respectively. The diameter of the hybridized nanoantenna unit is 0.38 µm. The height of PEDOT and dielectric layer are 0.2 and 0.25 µm, respectively. In H1 and H2 modes, we calculated that the slope of linear angular equation is 0.6~0.65r, corresponding to the mixture of first (slope = 0.75r) and second diffraction orders (slope = 0.5r) of RAs.

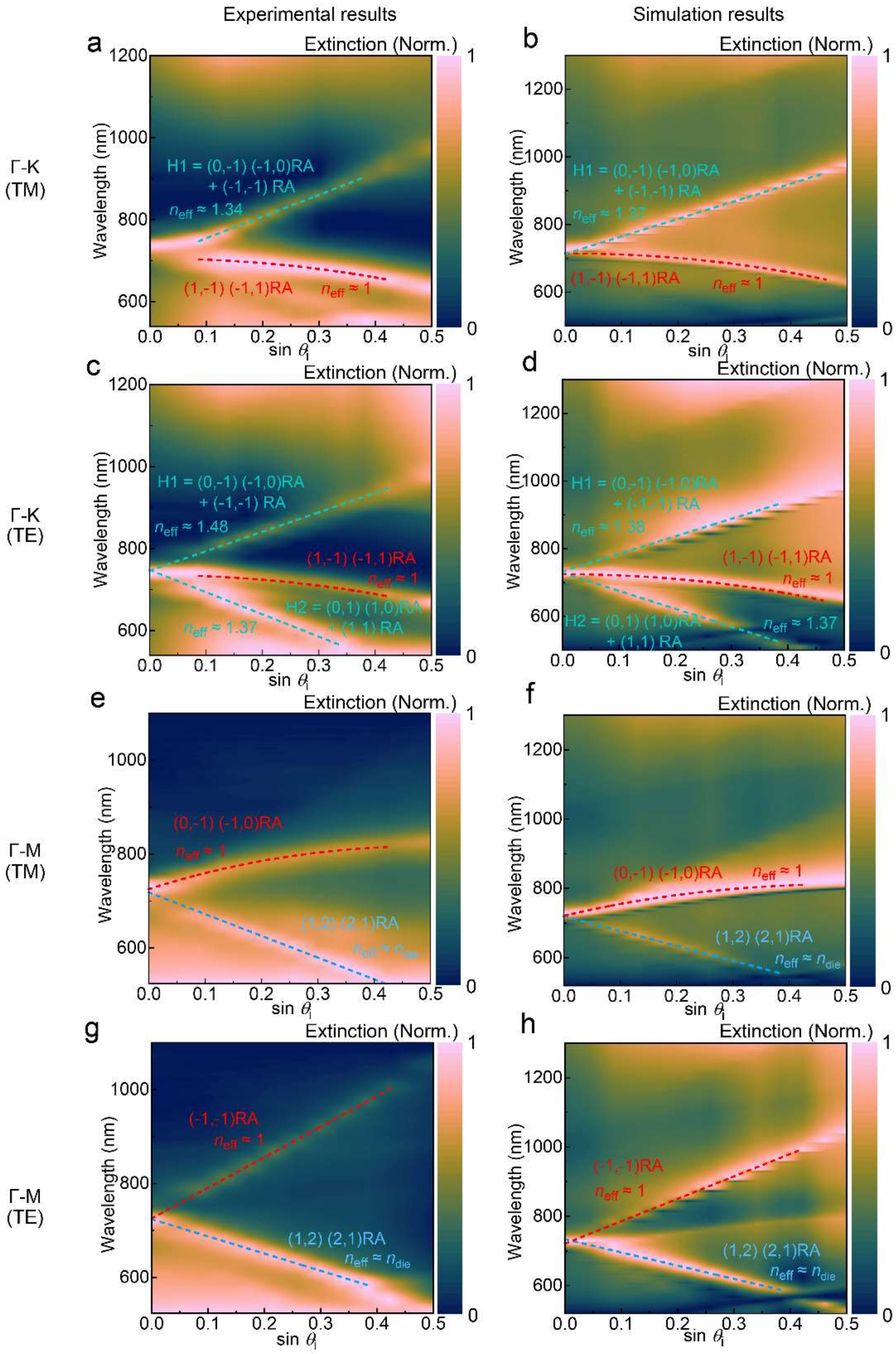

Figure S36. Angle-dependent extinction spectra of the hexagonal Die-PEDOT-Nano array (*r* = 0.8 μm). (a) (c) (e) (g) are experimental results in $\Gamma-M_{TM}$, $\Gamma-M_{TE}$, $\Gamma-K_{TM}$ and $\Gamma-K_{TE}$ modes, respectively. The diameter of the hybridized nanoantenna unit is 0.38 μm. (b) (d) (f) (h) are simulation results in $\Gamma-M_{TM}$, $\Gamma-M_{TE}$, $\Gamma-K_{TM}$ and $\Gamma-K_{TE}$ modes, respectively. The diameter of the hybridized nanoantenna unit is 0.4μm. The height of PEDOT and dielectric layers are 0.2 and 0.25 μm, respectively. In H1 and H2 modes, we calculated that the slope of linear angular equation is 0.59~0.67*r*, corresponding to the mixture of first (slope = 0.75*r*) and second diffraction orders (slope = 0.5*r*) of RAs.

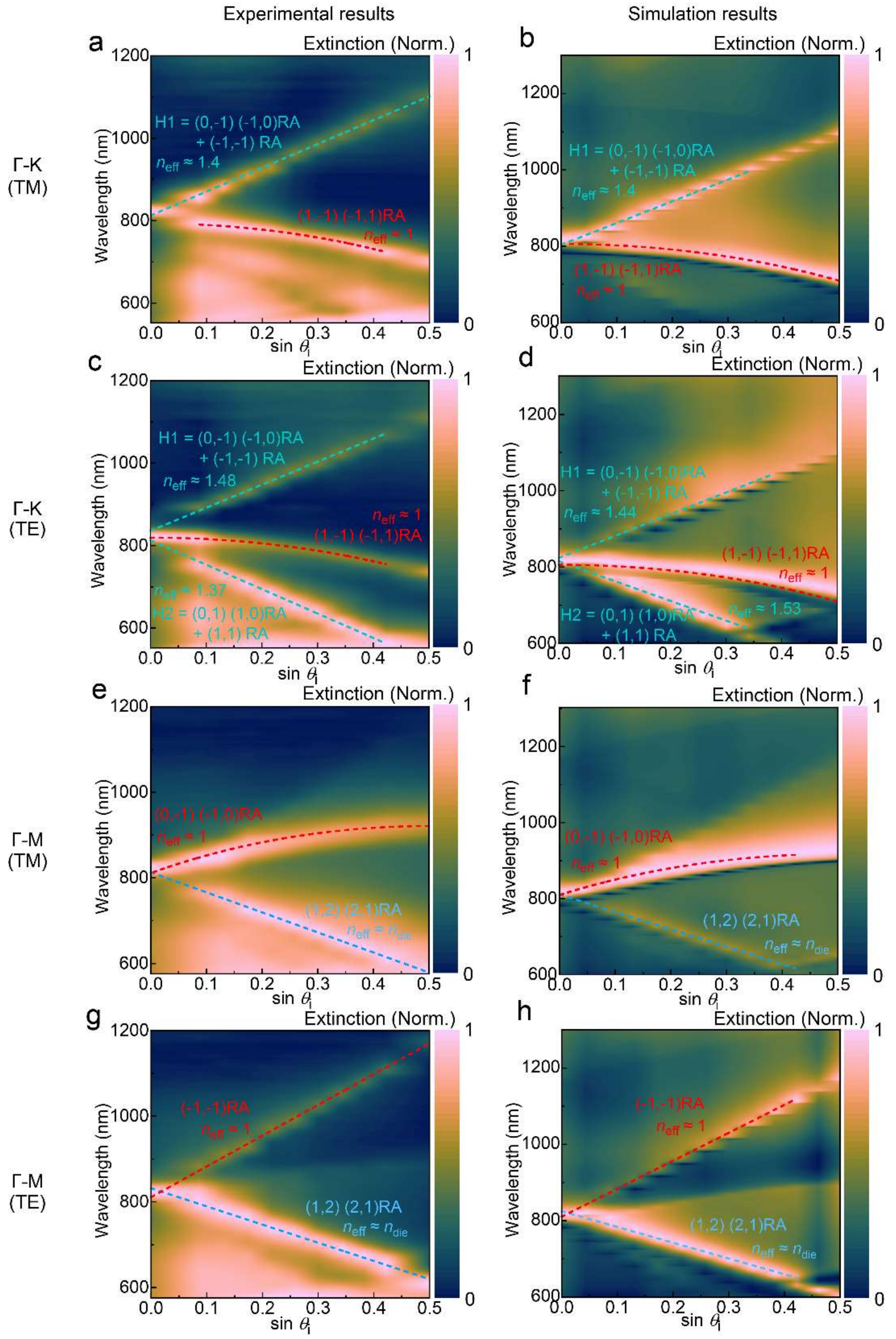

Figure S37. Angle-dependent extinction spectra of the hexagonal Die-PEDOT-Nano array (r = 0.9 μm). (a) (c) (e) (g) are experimental results in $\Gamma-M_{TM}$, $\Gamma-M_{TE}$, $\Gamma-K_{TM}$ and $\Gamma-K_{TE}$ modes, respectively. The diameter of the hybridized nanoantenna unit is 0.48 μm. (b) (d) (f) (h) are simulation results in $\Gamma-M_{TM}$, $\Gamma-M_{TE}$, $\Gamma-K_{TM}$ and $\Gamma-K_{TE}$ modes, respectively. The diameter of the hybridized nanoantenna unit is 0.5 μm. The height of PEDOT and dielectric layers are 0.2 and 0.25 μm, respectively. In H1 and H2 modes, we calculated that the slope of linear angular equation is 0.58~0.65r, corresponding to the mixture of first (slope = 0.75r) and second diffraction orders (slope = 0.5r) of RAs.

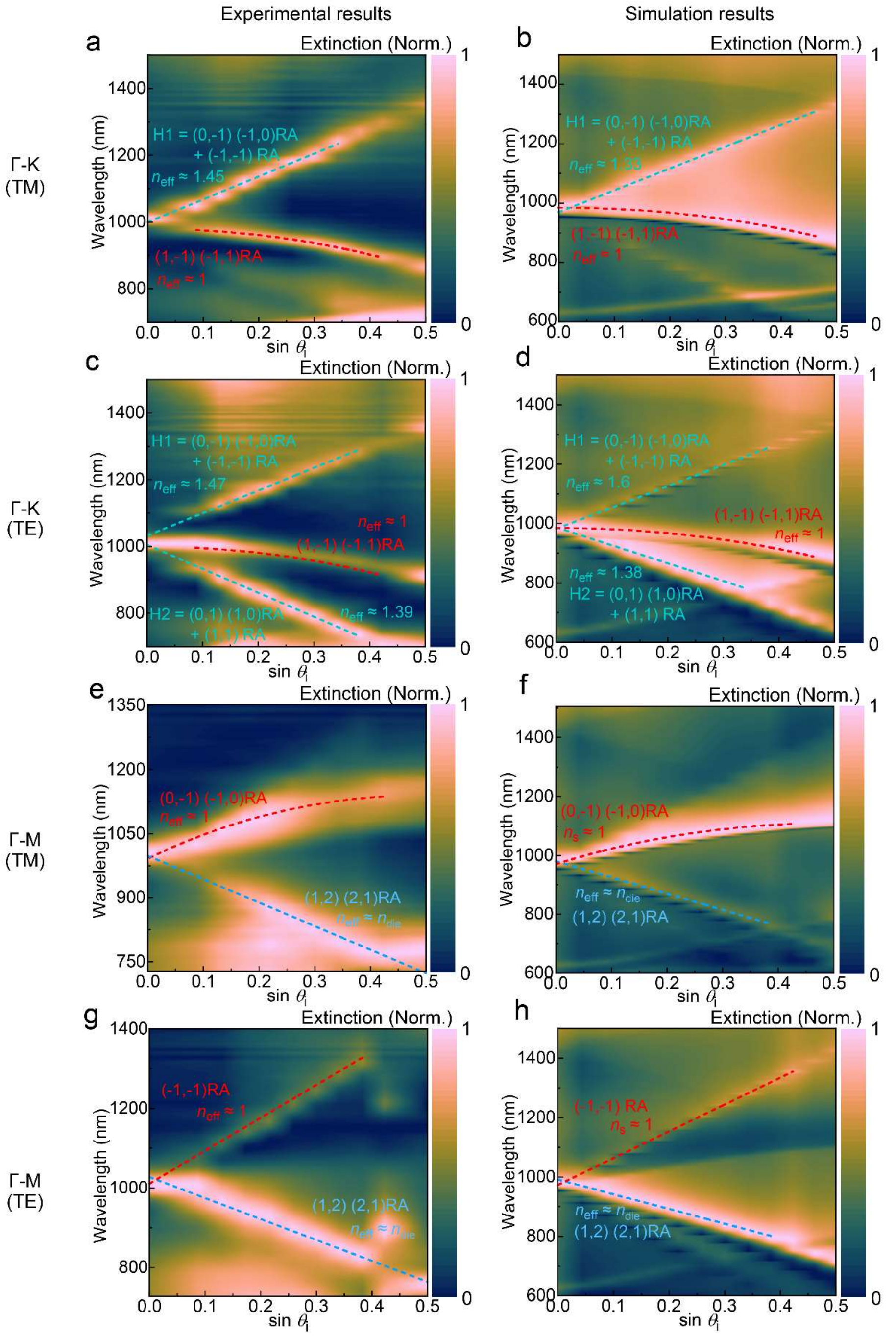

Figure S38. Angle-dependent extinction spectra of the hexagonal Die-PEDOT-Nano array (r = 1.1 μm). (a) (c) (e) (g) are experimental results in $\Gamma-M_{TM}$, $\Gamma-M_{TE}$, $\Gamma-K_{TM}$ and $\Gamma-K_{TE}$ modes, respectively. The diameter of the hybridized nanoantenna unit is 0.6 μm. (b) (d) (f) (h) are simulation results in $\Gamma-M_{TM}$, $\Gamma-M_{TE}$, $\Gamma-K_{TM}$ and $\Gamma-K_{TE}$ modes, respectively. The diameter of the hybridized nanoantenna unit is 0.62 μm. The height of PEDOT and dielectric layers are 0.2 and 0.25 μm, respectively. In H1 and H2 modes, we calculated that the slope of linear angular equation is 0.54~0.64r, corresponding to the mixture of first (slope = 0.75r) and second diffraction orders (slope = 0.5r) of RAs.

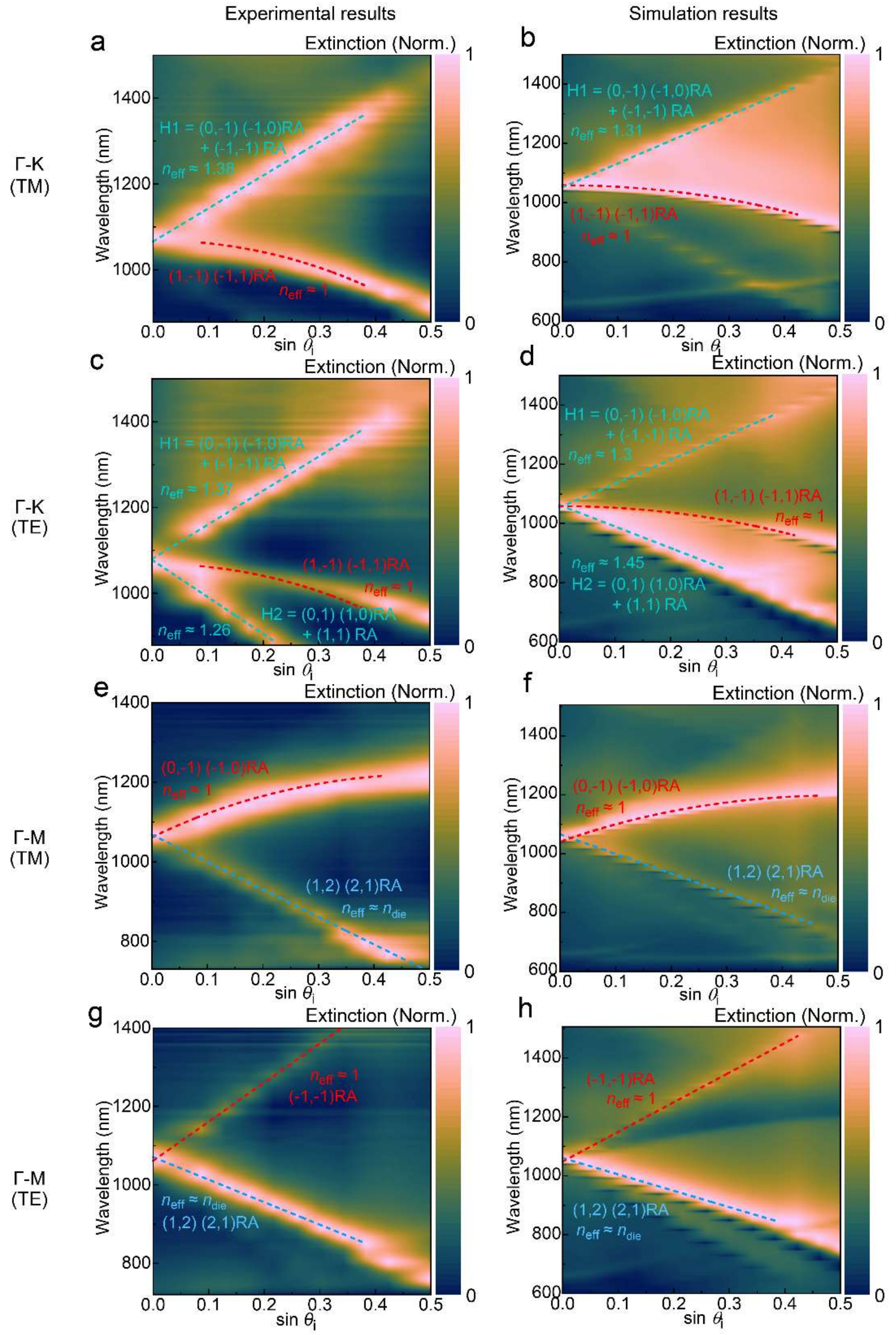

Figure S39. Angle-dependent extinction spectra of the hexagonal Die-PEDOT-Nano array (*r* = 1.2 μm). (a) (c) (e) (g) are experimental results in $\Gamma{-}M_{TM}$, $\Gamma{-}M_{TE}$, $\Gamma{-}K_{TM}$ and $\Gamma{-}K_{TE}$ modes, respectively. The diameter of the hybridized nanoantenna unit is 0.6 μm. (b) (d) (f) (h) are simulation results in $\Gamma{-}M_{TM}$, $\Gamma{-}M_{TE}$, $\Gamma{-}K_{TM}$ and $\Gamma{-}K_{TE}$ modes, respectively. The diameter of the hybridized nanoantenna unit is 0.62 μm. The height of PEDOT and dielectric layers are 0.2 and 0.25 μm, respectively. In H1 and H2 modes, we calculated that the slope of linear angular equation is 0.6~0.67*r*, corresponding to the mixture of first (slope = 0.75*r*) and second diffraction orders (slope = 0.5*r*) of RAs.

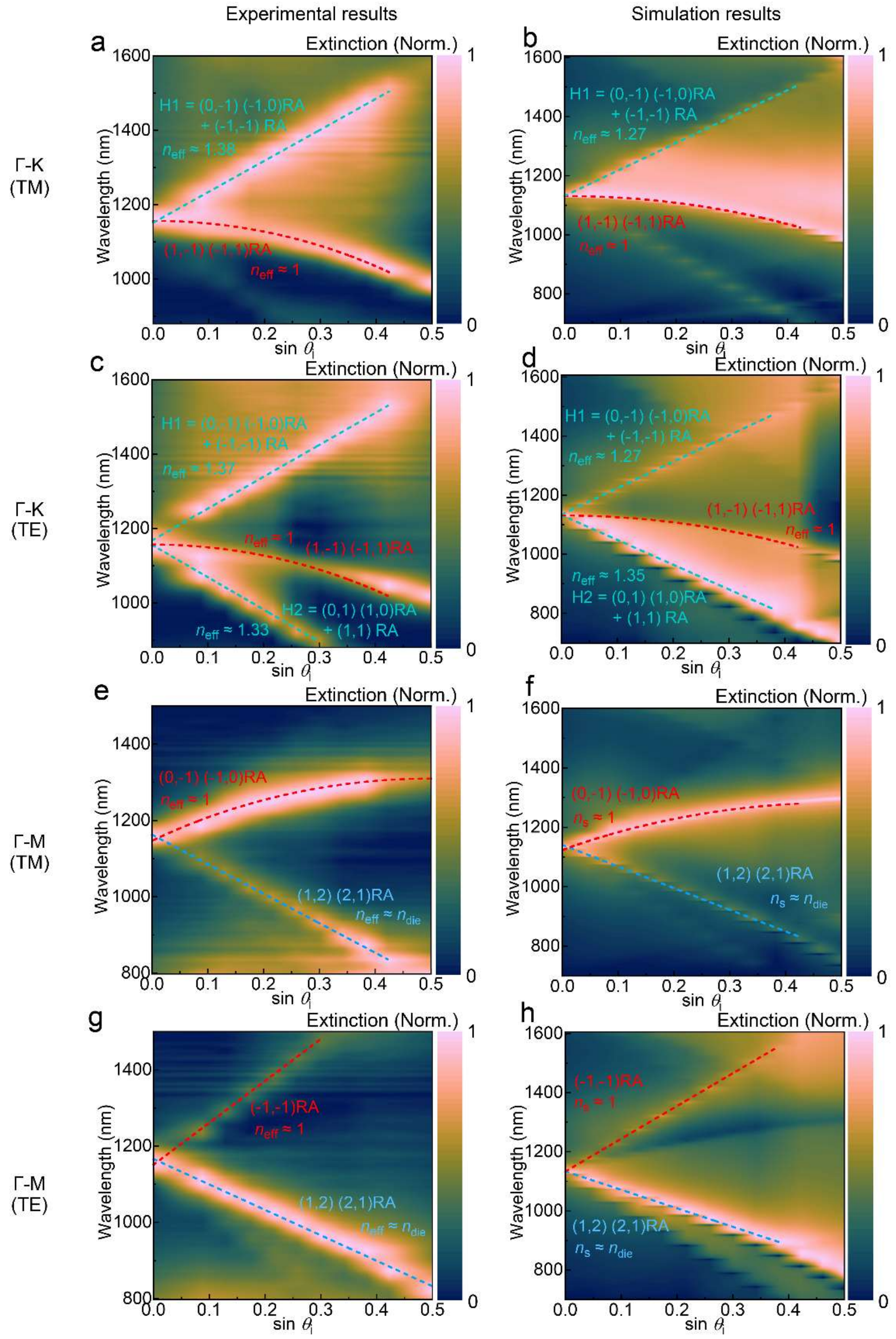

Figure S40. Angle-dependent extinction spectra of the hexagonal Die-PEDOT-Nano array (*r* = 1.3 μm). (a) (c) (e) (g) are experimental results in $\Gamma-M_{TM}$, $\Gamma-M_{TE}$, $\Gamma-K_{TM}$ and $\Gamma-K_{TE}$ modes, respectively. The diameter of the hybridized nanoantenna unit is 0.61 μm. (b) (d) (f) (h) are simulation results in $\Gamma-M_{TM}$, $\Gamma-M_{TE}$, $\Gamma-K_{TM}$ and $\Gamma-K_{TE}$ modes, respectively. The diameter of the hybridized nanoantenna unit is 0.62 μm. The height of PEDOT and dielectric layers are 0.2 and 0.25 μm, respectively. In H1 and H2 modes, we calculated that the slope of linear angular equation is 0.62~0.68*r*, corresponding to the mixture of first (slope = 0.75*r*) and second diffraction orders (slope = 0.5*r*) of RAs.

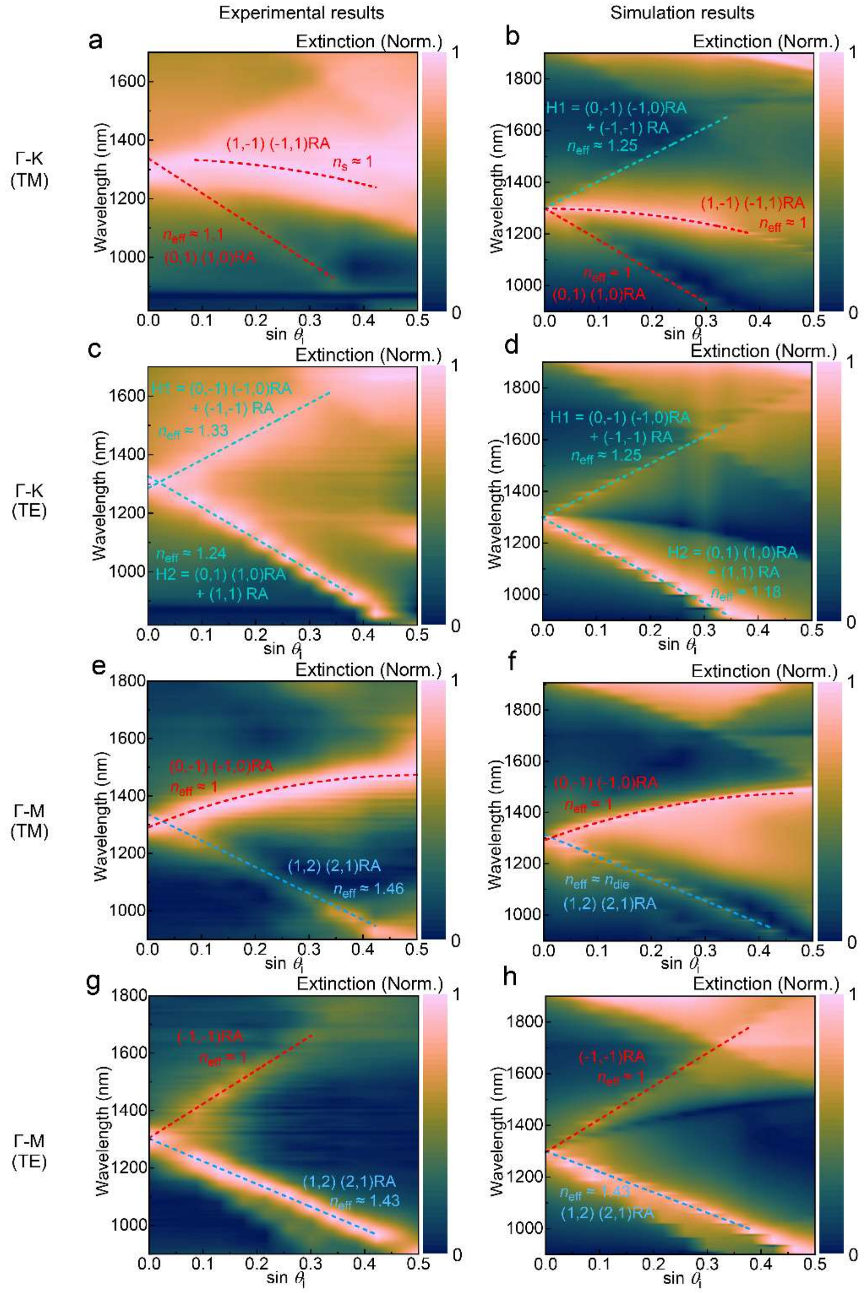

Figure S41. Angle-dependent extinction spectra of the hexagonal Die-PEDOT-Nano array (r = 1.5 μm). (a) (c) (e) (g) are experimental results in $\Gamma-\mathrm{M}_{\mathrm{TM}}$, $\Gamma-\mathrm{M}_{\mathrm{TE}}$, $\Gamma-\mathrm{K}_{\mathrm{TM}}$ and $\Gamma-\mathrm{K}_{\mathrm{TE}}$ modes, respectively. The diameter of the hybridized nanoantenna unit is 0.62 μm. (b) (d) (f) (h) are simulation results in $\Gamma-\mathrm{M}_{\mathrm{TM}}$, $\Gamma-\mathrm{M}_{\mathrm{TE}}$, $\Gamma-\mathrm{K}_{\mathrm{TM}}$ and $\Gamma-\mathrm{K}_{\mathrm{TE}}$ modes, respectively. The diameter of the hybridized nanoantenna unit is 0.62 μm. The height of PEDOT and dielectric layers are 0.2 and 0.25 μm, respectively. In H1 and H2 modes, we calculated that the slope of linear angular equation is 0.69~0.73r, corresponding to the mixture of first (slope = 0.75r) and second diffraction orders (slope = 0.5r) of RAs. It is noted that n_{eff} is obviously lower than those in short-r hexagonal arrays, which is probably due to the diminished intensity of electric dipole resonance within dielectric nanocylinders. In this case, the contribution from second-order diffraction grating effect is also reduced, and the coupling is mainly derived from the first diffraction orders of RAs.

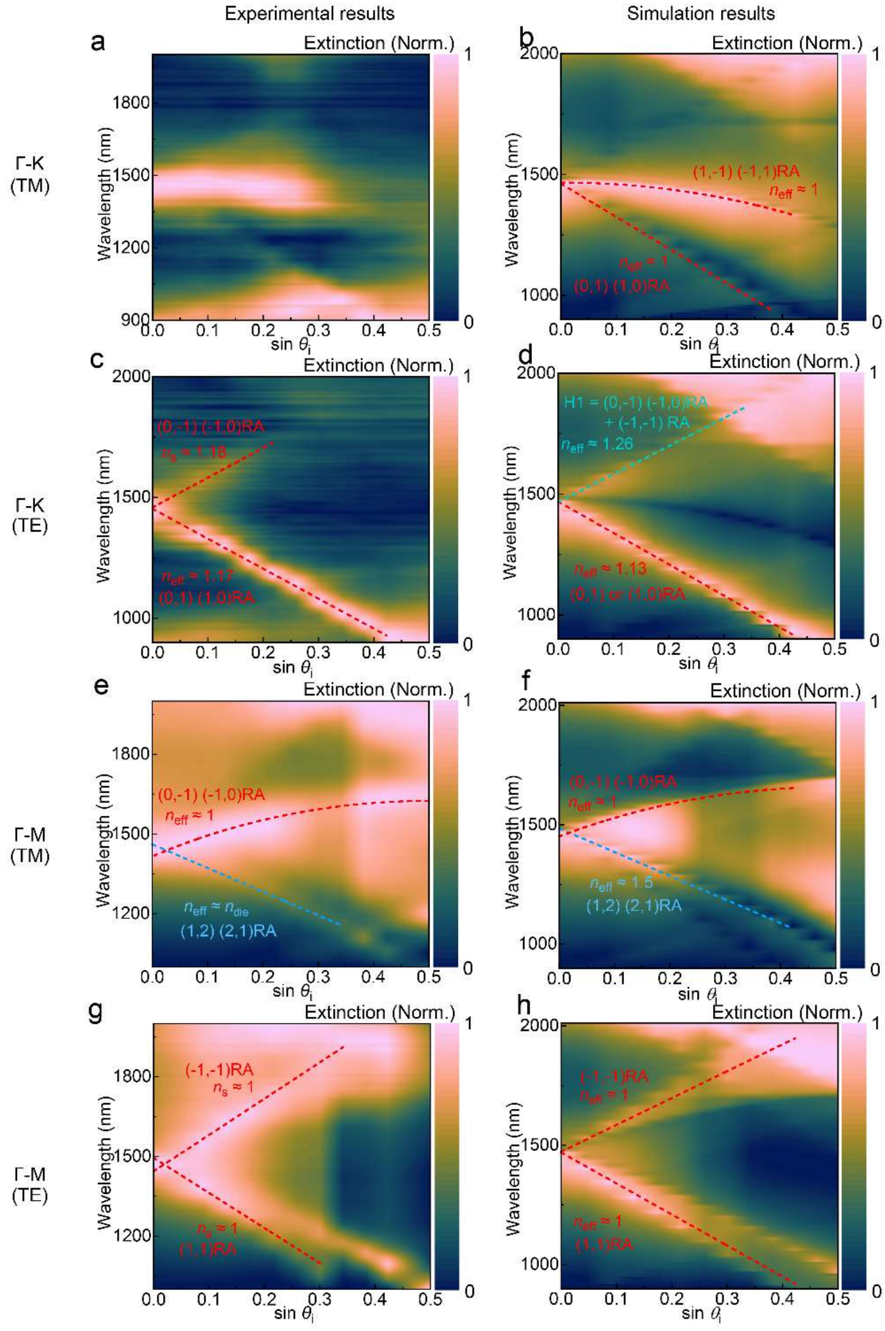

Figure S42. Angle-dependent extinction spectra of the hexagonal Die-PEDOT-Nano array (*r* = 1.7 μm). (a) (c) (e) (g) are experimental results in $\Gamma-M_{TM}$, $\Gamma-M_{TE}$, $\Gamma-K_{TM}$ and $\Gamma-K_{TE}$ modes, respectively. The diameter of the hybridized nanoantenna unit is 0.62 μm. (b) (d) (f) (h) are simulation results in $\Gamma-M_{TM}$, $\Gamma-M_{TE}$, $\Gamma-K_{TM}$ and $\Gamma-K_{TE}$ modes, respectively. The diameter of the hybridized nanoantenna unit is 0.62 μm. The height of PEDOT and dielectric layers are 0.2 and 0.25 μm, respectively. Here we can find that the mixing coupling modes along the Γ−K direction are almost eliminated, and the second diffraction orders of (1,2) (2,1) RAs are also vanished in $\Gamma-M_{TE}$ mode. These results are related to sharply eliminated electric dipole resonance within dielectric nanocylinders.

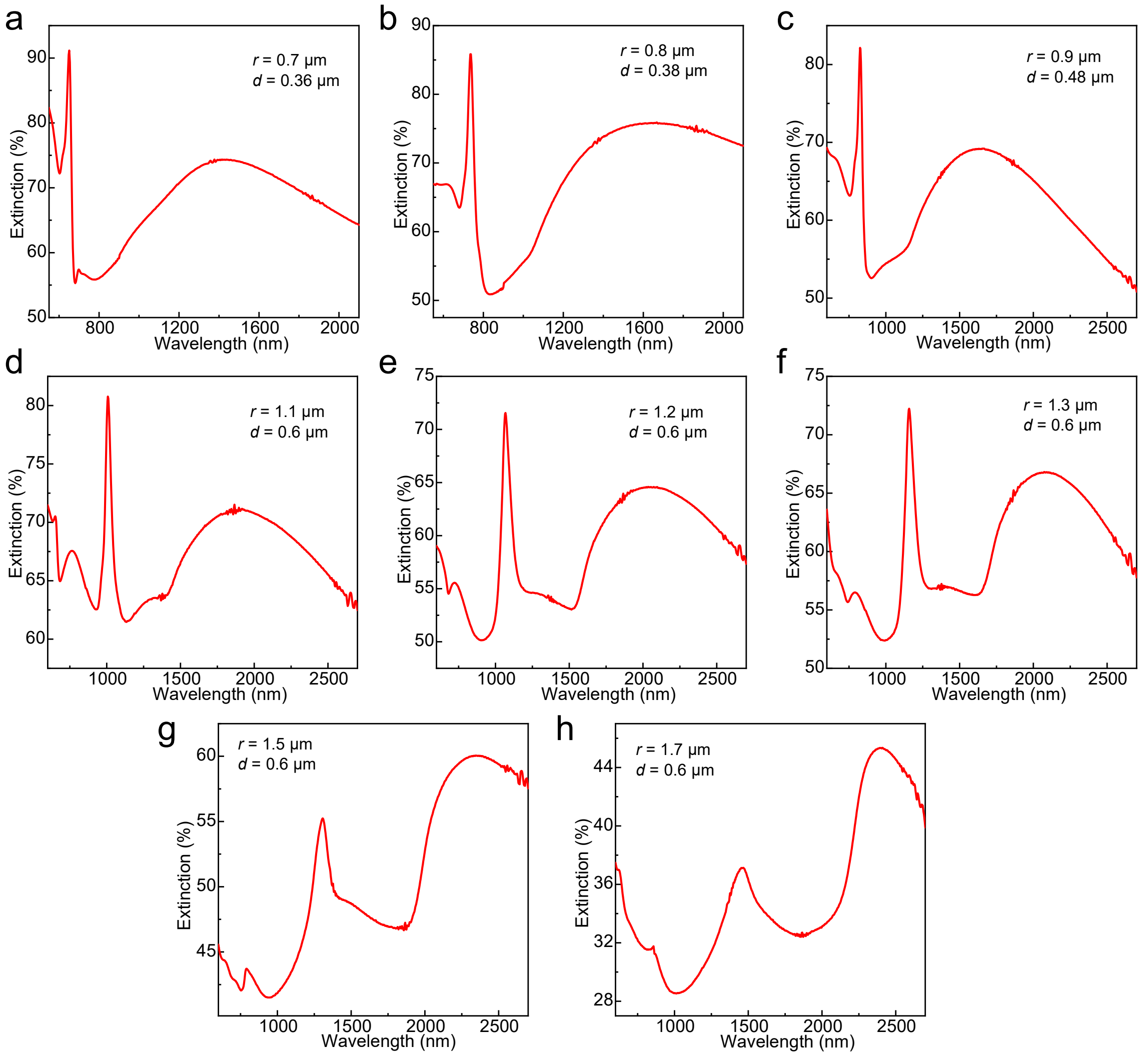

Figure S43. Extinction spectra (experimental results) of hexagonal Die-PEDOT-Nano arrays. The periodicity includes r = 0.7 (a, d = 0.36 μm), 0.8 (b, d = 0.36 μm), 0.9 (c, d = 0.48 μm), 1.1 (d, d = 0.6 μm), 1.2 (e, d = 0.6 μm), 1.3 (f, d = 0.6 μm), 1.5 (g, d = 0.6 μm), and 1.7 μm (h, d = 0.6 μm). The height of dielectric is about 0.25-0.3 μm, and the height of PEDOT layer is about 0.2 μm.

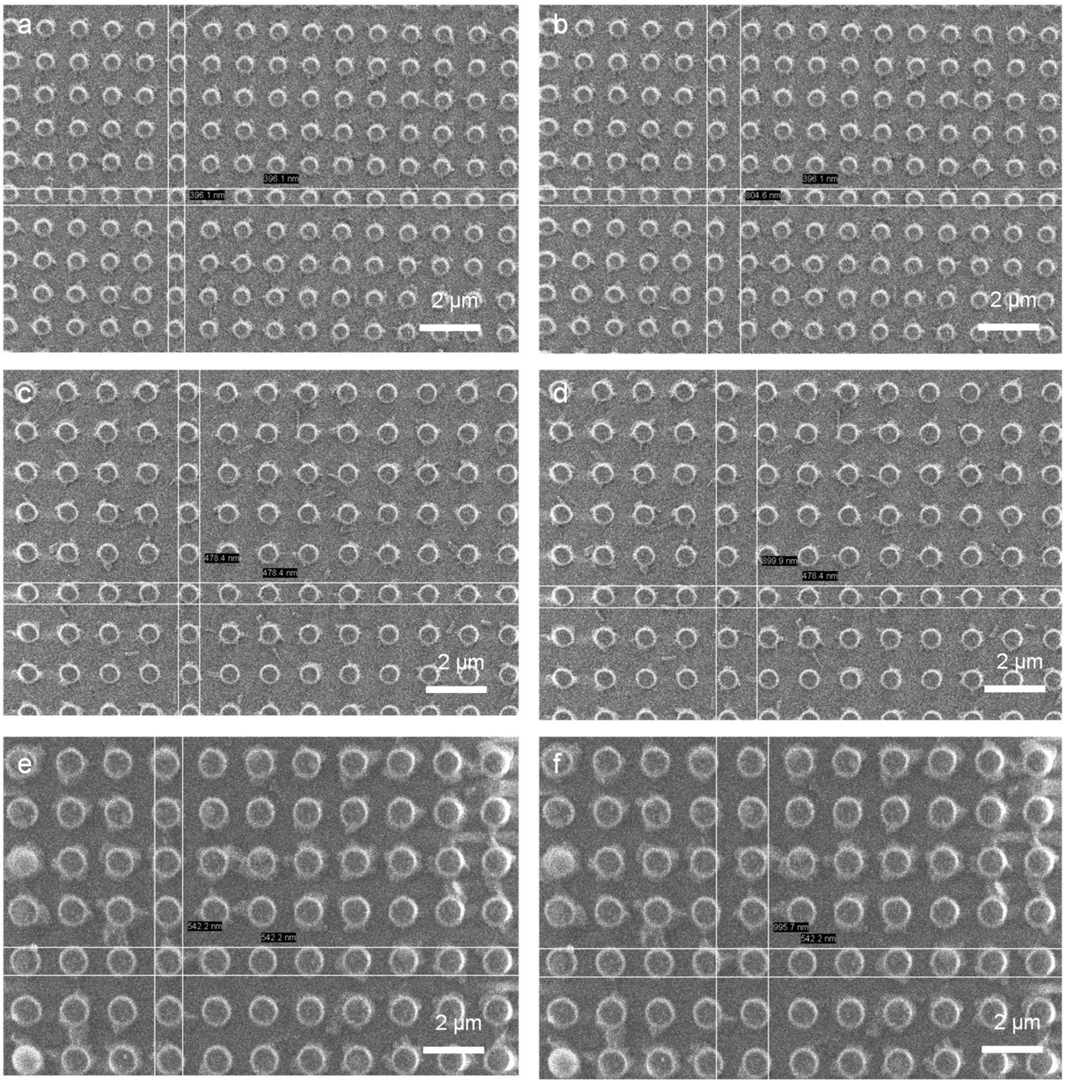

Figure S44. SEM images of Die-EDOT-Nano arrays prepared through electron beam lithography (square shapes, *r* = 0.8-1.0 μm**).** The hybridized nanoantenna units have diameters *d* = 0.4, 0.48 and 0.54 μm in a square array with the periodicity *r* = 0.8 (in [a] and [b]), 0.9 (in [c] and [d]) and 1.0 μm (in [e] and [f]), respectively.

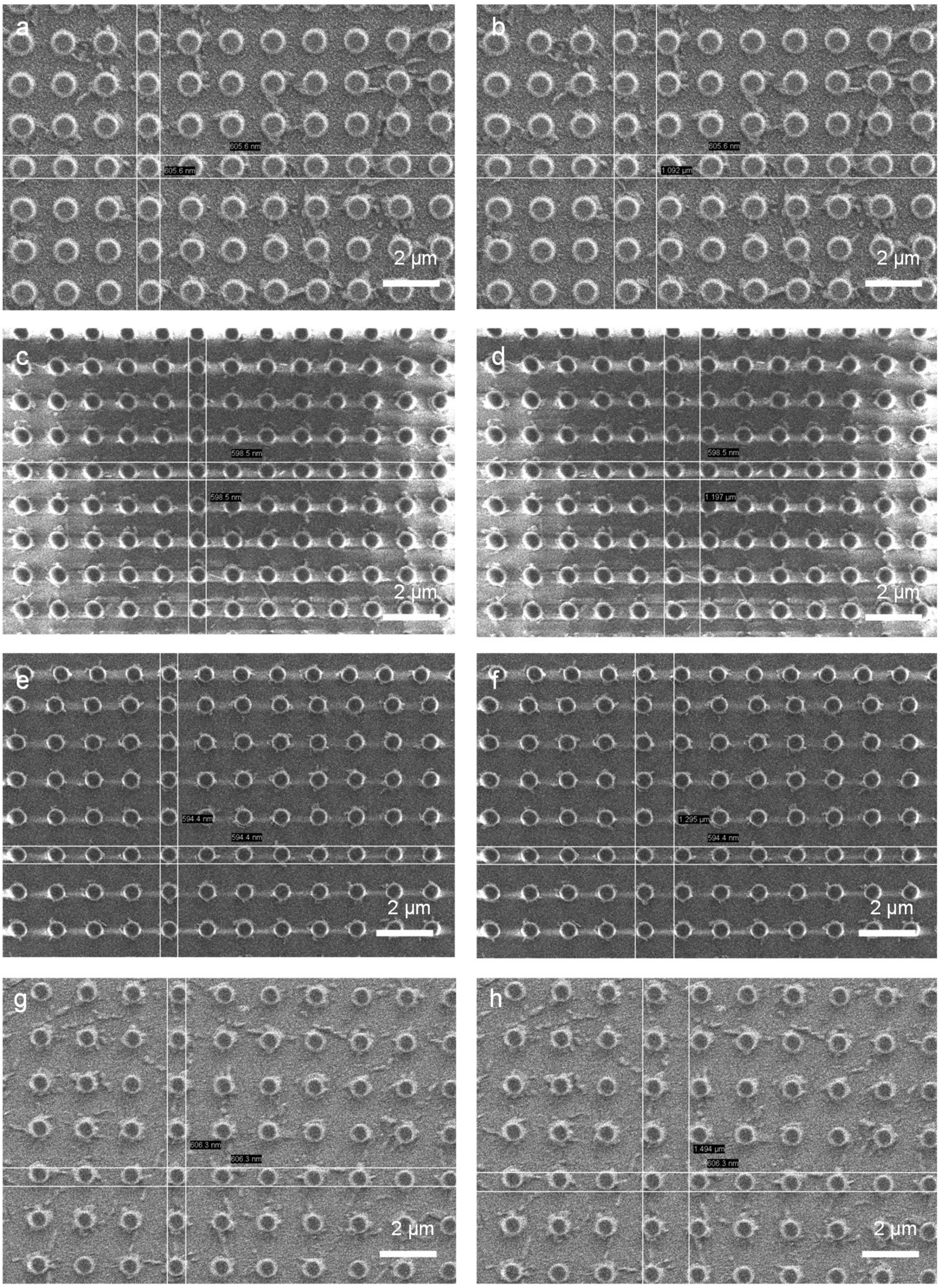

Figure S45. SEM images of Die-EDOT-Nano arrays prepared through electron beam lithography (square shapes, *r* = 1.1-1.5 µm). The hybridized nanoantenna unit has a diameter *d* = 0.6-0.61 µm in a square array with the periodicity *r* = 1.1 (in [a] and [b]), 1.2 (in [c] and [d]), 1.3 (in [e] and [f]) and 1.5 µm (in [g] and [h]), respectively.

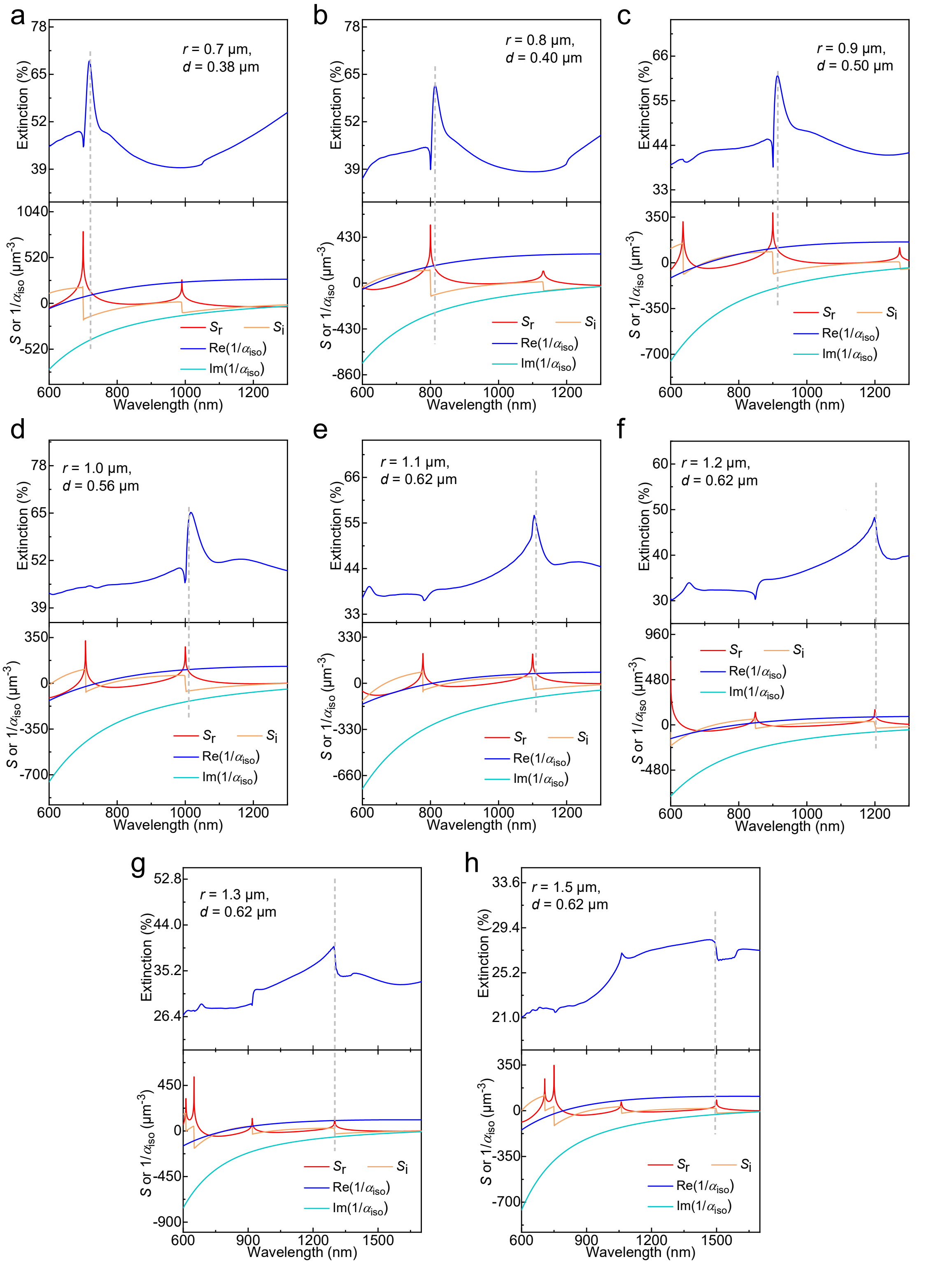

Figure S46. Analysis of CLR-matching conditions and extinction spectra for square Die-PEDOT-Nano arrays with different *r*. The acid-treated PEDOT:ToS was selected for the part of conducting polymer nanoantennas. The extinction spectra were achieved via FDTD simulations. In all panels, the dielectric nanocylinders (n_{die} = 1.60) have a diameter d = 0.56 μm and a height h = 0.25 μm. The height of PEDOT is 0.2 μm.

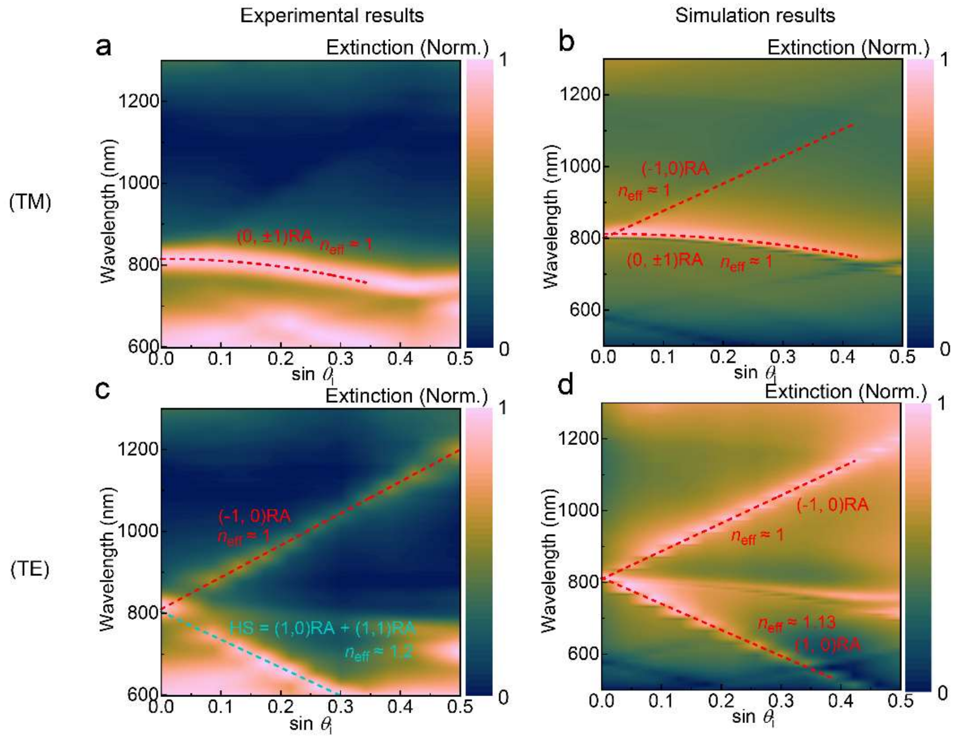

Figure S47. Angle-dependent extinction spectra of the square Die-PEDOT-Nano array (*r* = 0.8 μm). (a) (c) are experimental results in $\Gamma{-}M_{TM}$, $\Gamma{-}M_{TE}$, $\Gamma{-}K_{TM}$ and $\Gamma{-}K_{TE}$ modes, respectively. The diameter of the hybridized nanoantenna unit is 0.4 μm. (b) (d) are simulation results in $\Gamma{-}M_{TM}$, $\Gamma{-}M_{TE}$, $\Gamma{-}K_{TM}$ and $\Gamma{-}K_{TE}$ modes, respectively. The diameter of the hybridized nanoantenna unit is 0.4 μm. The only mixed coupling (HS) can be showed in TE mode (experimental results) with $n_{\mathrm{eff}} = 1.2$ and the slope of linear angular equation equaling $0.85r$, approximating the intermediate part between the first diffraction orders of (1,0) RAs ($\lambda_r = r\sin\theta_i + rn_{\mathrm{eff}}$) and the second diffraction orders of (1,1) RA ($\lambda_r \approx \frac{1}{2}r\sin\theta_i + \frac{\sqrt{2}}{2}rn_{\mathrm{eff}}$). It is noted that n_{eff} is obviously lower than $0.5(n_{\mathrm{air}} + n_{\mathrm{die}})$ and the slope of HS angular equation is more approximate to the first diffraction orders, reflecting that the dominant coupling comes from the first diffraction orders.

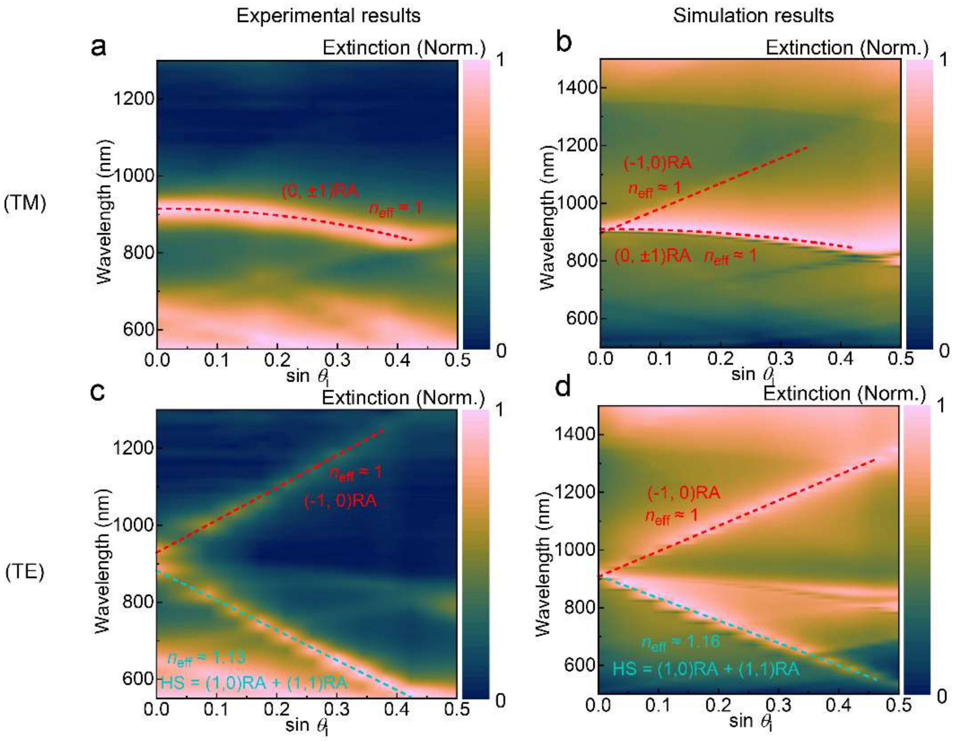

Figure S48. Angle-dependent extinction spectra of the square Die-PEDOT-Nano array (*r* = 0.9 μm). (a) (c) are experimental results in $\Gamma-M_{TM}$, $\Gamma-M_{TE}$, $\Gamma-K_{TM}$ and $\Gamma-K_{TE}$ modes, respectively. The diameter of the hybridized nanoantenna unit is 0.48 μm. (b) (d) are simulation results in $\Gamma-M_{TM}$, $\Gamma-M_{TE}$, $\Gamma-K_{TM}$ and $\Gamma-K_{TE}$ modes, respectively. The diameter of the hybridized nanoantenna unit is 0.5 μm. The only mixed coupling (HS) can be showed in TE mode (experimental results) with n_{eff} = 1.13-1.16 and the slope of linear angular equation equaling 0.87r, approximating the intermediate part between the first diffraction orders of (1,0) RAs ($\lambda_r = r\sin\theta_i + rn_{eff}$) and the second diffraction orders of (1,1) RA ($\lambda_r \approx \frac{1}{2}r\sin\theta_i + \frac{\sqrt{2}}{2}rn_{eff}$). It is noted that n_{eff} is obviously lower than $0.5(n_{air} + n_{die})$ and the slope of HS angular equation is more approximate to the first diffraction orders.

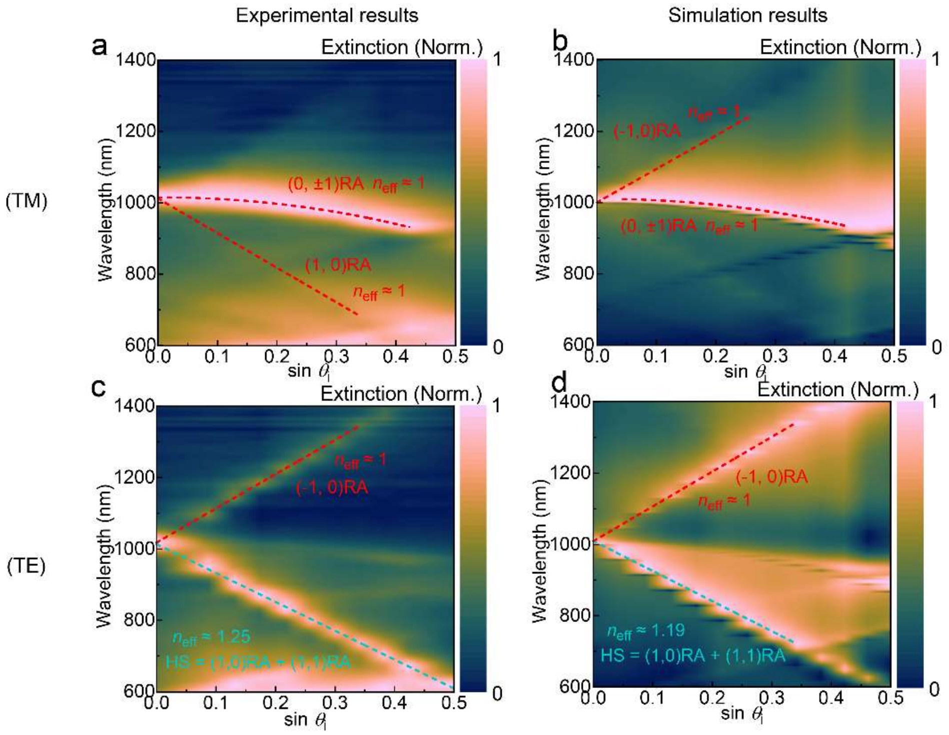

Figure S49. Angle-dependent extinction spectra of the square Die-PEDOT-Nano array (*r* = 1.0 µm). (a) (c) are experimental results in Γ−M$_{TM}$, Γ−M$_{TE}$, Γ−K$_{TM}$ and Γ−K$_{TE}$ modes, respectively. The diameter of the hybridized nanoantenna unit is 0.54 µm. (b) (d) are simulation results in Γ−M$_{TM}$, Γ−M$_{TE}$, Γ−K$_{TM}$ and Γ−K$_{TE}$ modes, respectively. The diameter of the hybridized nanoantenna unit is 0.54 µm. The only mixed coupling (HS) can be showed in TE mode (experimental results) with n_{eff} = 1.20-1.25 and the slope of linear angular equation equaling 0.80-0.84*r*, approximating the intermediate part between the first diffraction orders of (1,0) RAs ($\lambda_r = r\sin\theta_i + rn_{\mathrm{eff}}$) and the second diffraction orders of (1,1) RA ($\lambda_r \approx \frac{1}{2}r\sin\theta_i + \frac{\sqrt{2}}{2}rn_{\mathrm{eff}}$). It is noted that n_{eff} is obviously lower than $0.5(n_{\mathrm{air}} + n_{\mathrm{die}})$ and the slope of HS angular equation is more approximate to the first diffraction orders.

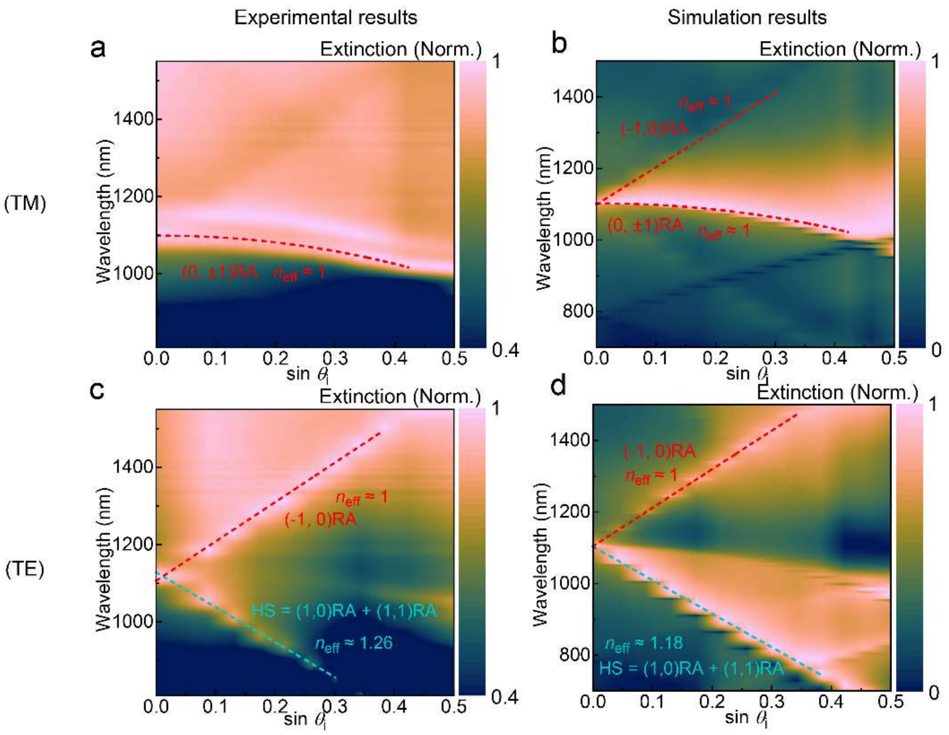

Figure S50. Angle-dependent extinction spectra of the square Die-PEDOT-Nano array (*r* = 1.1 μm). (a) (c) are experimental results in $\Gamma-M_{TM}$, $\Gamma-M_{TE}$, $\Gamma-K_{TM}$ and $\Gamma-K_{TE}$ modes, respectively. The diameter of the hybridized nanoantenna unit is 0.6 μm. (b) (d) are simulation results in $\Gamma-M_{TM}$, $\Gamma-M_{TE}$, $\Gamma-K_{TM}$ and $\Gamma-K_{TE}$ modes, respectively. The diameter of the hybridized nanoantenna unit is 0.62 μm. The only mixed coupling (HS) can be showed in TE mode (experimental results) with n_{eff} = 1.18-1.26 and the slope of linear angular equation equaling 0.85r, approximating the intermediate part between the first diffraction orders of (1,0) RAs ($\lambda_r = r\sin\theta_i + rn_{eff}$) and the second diffraction orders of (1,1) RA ($\lambda_r \approx \frac{1}{2}r\sin\theta_i + \frac{\sqrt{2}}{2}rn_{eff}$). It is noted that n_{eff} is obviously lower than $0.5(n_{air} + n_{die})$ and the slope of HS angular equation is more approximate to the first diffraction orders.

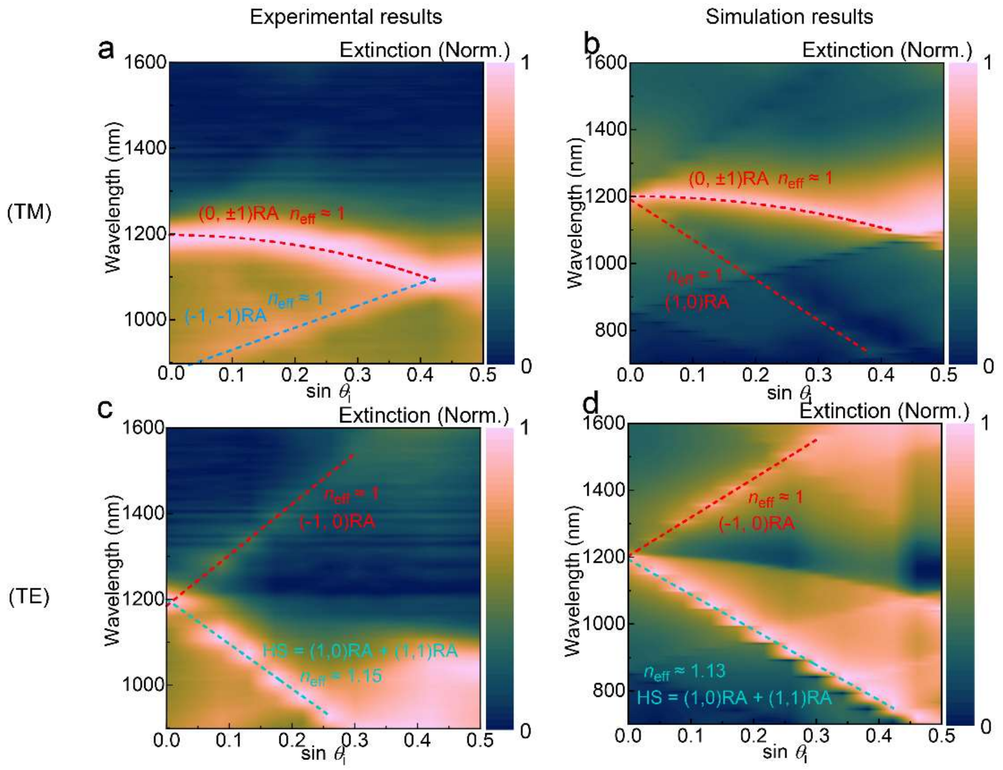

Figure S51. Angle-dependent extinction spectra of the square Die-PEDOT-Nano array (*r* = 1.2 μm). (a) (c) are experimental results in $\Gamma-M_{TM}$, $\Gamma-M_{TE}$, $\Gamma-K_{TM}$ and $\Gamma-K_{TE}$ modes, respectively. The diameter of the hybridized nanoantenna unit is 0.6 μm. (b) (d) are simulation results in $\Gamma-M_{TM}$, $\Gamma-M_{TE}$, $\Gamma-K_{TM}$ and $\Gamma-K_{TE}$ modes, respectively. The diameter of the hybridized nanoantenna unit is 0.62 μm. The only mixed coupling (HS) can be showed in TE mode (experimental results) with $n_{eff} = 1.13$ and the slope of linear angular equation equaling 0.87*r*, approximating the intermediate part between the first diffraction orders of (1,0) RAs ($\lambda_r = r\sin\theta_i + rn_{eff}$) and the second diffraction orders of (1,1) RA ($\lambda_r \approx \frac{1}{2}r\sin\theta_i + \frac{\sqrt{2}}{2}rn_{eff}$). It is noted that n_{eff} is obviously lower than $0.5(n_{air} + n_{die})$ and the slope of HS angular equation is more approximate to the first diffraction orders.

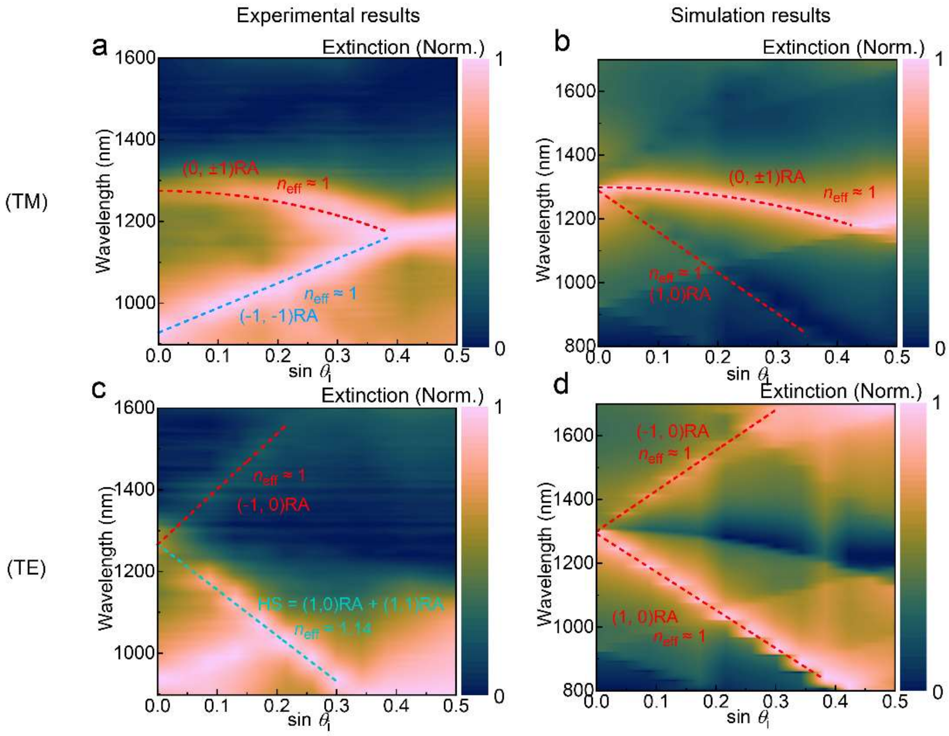

Figure S52. Angle-dependent extinction spectra of the square Die-PEDOT-Nano array (*r* = 1.3 µm). (a) (c) are experimental results in $\Gamma-M_{TM}$, $\Gamma-M_{TE}$, $\Gamma-K_{TM}$ and $\Gamma-K_{TE}$ modes, respectively. The diameter of the hybridized nanoantenna unit is 0.6 µm. (b) (d) are simulation results in $\Gamma-M_{TM}$, $\Gamma-M_{TE}$, $\Gamma-K_{TM}$ and $\Gamma-K_{TE}$ modes, respectively. The diameter of the hybridized nanoantenna unit is 0.62 µm. The only mixed coupling (HS) can be showed in TE mode (experimental results) with $n_{eff} = 1.14$ and the slope of linear angular equation equaling 0.85*r*, approximating the intermediate part between the first diffraction orders of (1,0) RAs ($\lambda_r = r\sin\theta_i + rn_{eff}$) and the second diffraction orders of (1,1) RA ($\lambda_r \approx \frac{1}{2}r\sin\theta_i + \frac{\sqrt{2}}{2}rn_{eff}$). It is noted that n_{eff} is obviously lower than $0.5(n_{air} + n_{die})$ and the slope of HS angular equation is more approximate to the first diffraction orders.

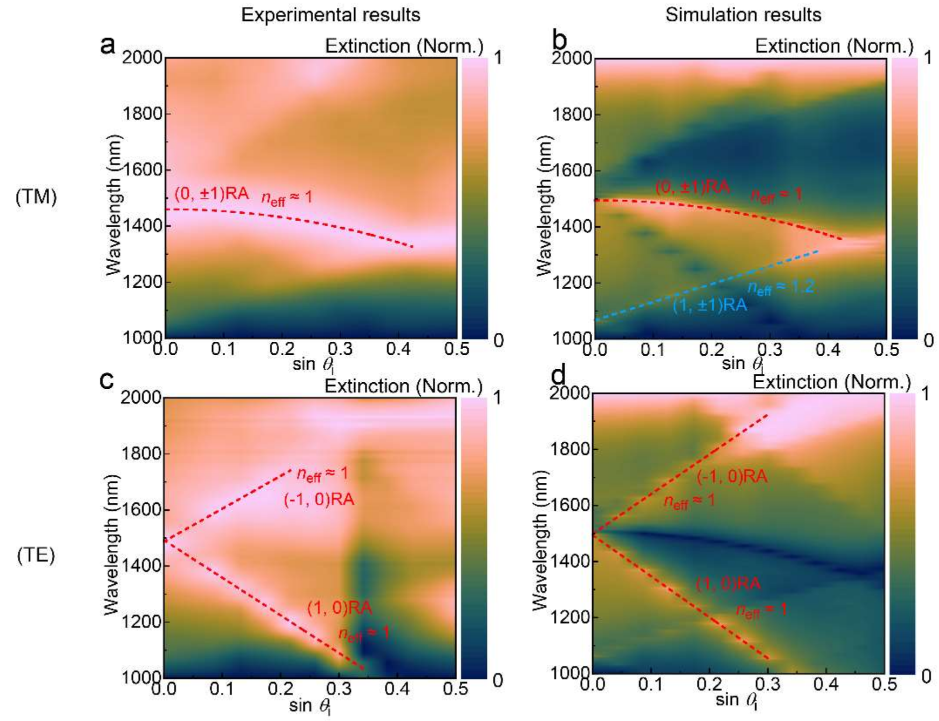

Figure S53. Angle-dependent extinction spectra of the square Die-PEDOT-Nano array (*r* = 1.5 μm). (a) (c) are experimental results in $\Gamma-M_{TM}$, $\Gamma-M_{TE}$, $\Gamma-K_{TM}$ and $\Gamma-K_{TE}$ modes, respectively. The diameter of the hybridized nanoantenna unit is 0.61 μm. (b) (d) are simulation results in $\Gamma-M_{TM}$, $\Gamma-M_{TE}$, $\Gamma-K_{TM}$ and $\Gamma-K_{TE}$ modes, respectively. The diameter of the hybridized nanoantenna unit is 0.62 μm.

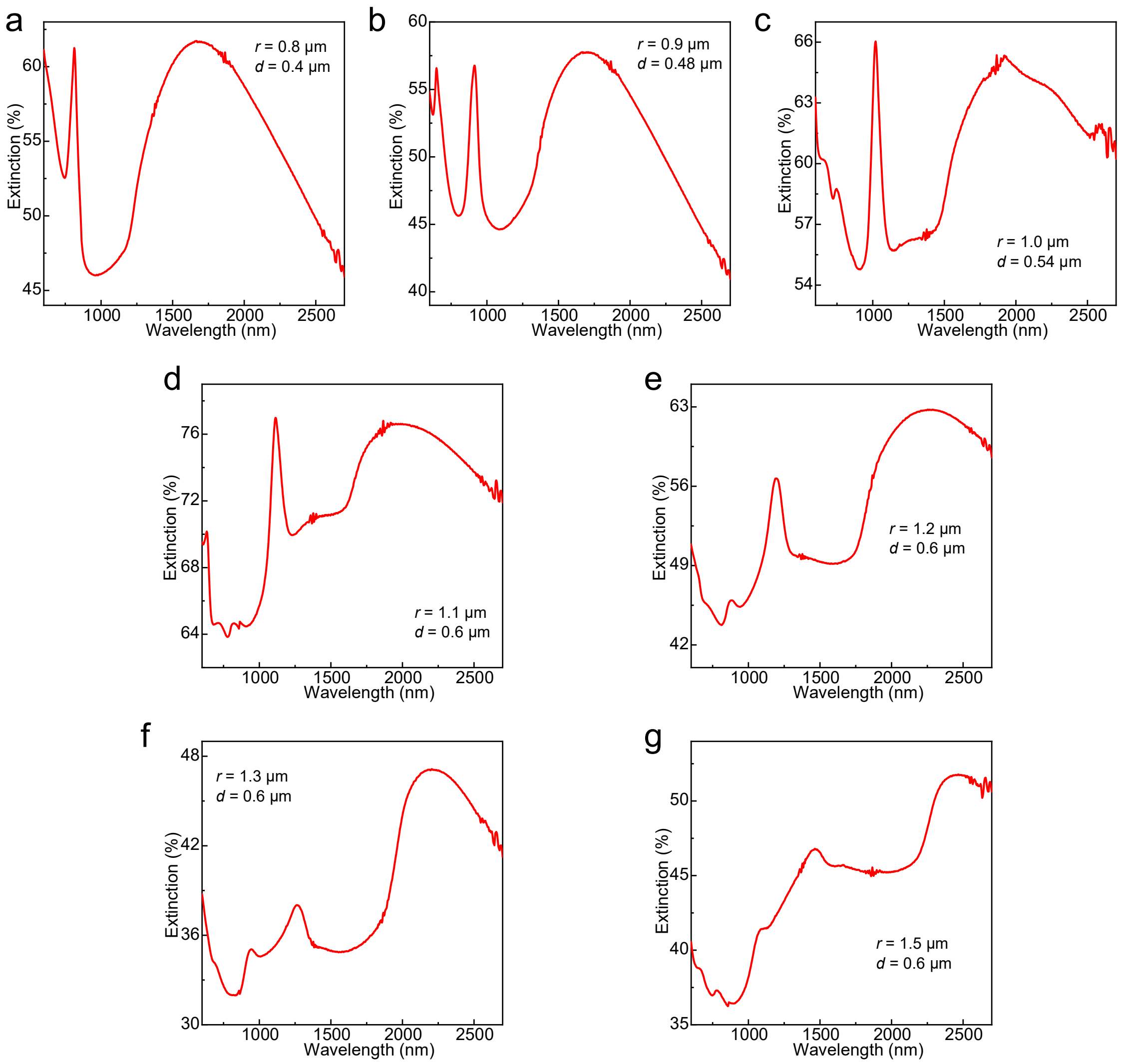

Figure S54. Extinction spectra (experimental results) of square Die-PEDOT-Nano arrays. The periodicity includes 0.8 (a, $d = 0.36$ μm), 0.9 (b, $d = 0.48$ μm), 1.0 (c, $d = 0.54$ μm), 1.1 (e, $d = 0.6$ μm), 1.2 (f, $d = 0.6$ μm), 1.3 (g, $d = 0.6$ μm), and 1.5 μm (h, $d = 0.6$ μm). The height of dielectric is about 0.25-0.3 μm, and the height of PEDOT layer is about 0.2 μm.

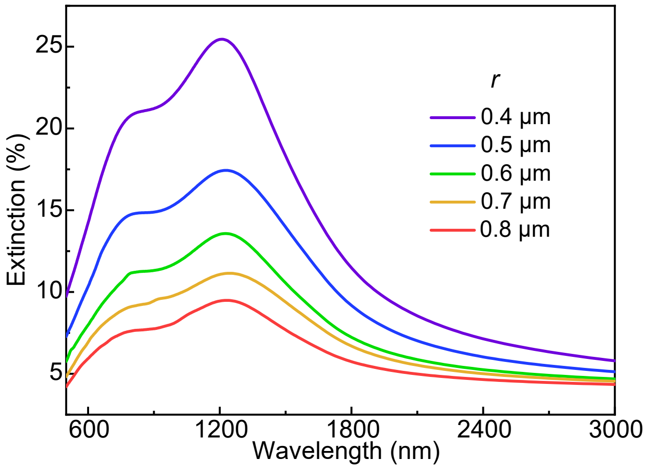

Figure S55. Extinction spectra (FDTD results) of the PEDOT nanoantenna array (without dielectric nanocylinders). The PEDOT nanoantennas have a diameter $d = 0.16$ μm and a height $h = 0.2$ μm. The PEDOT materials are acid-treated PEDOT:ToS. The periodicity r ranges from

0.4 to 0.8 μm. Along with increasing *r*, the resonance wavelength is still fixed around 1200 nm, suggesting that such surface plasmon resonance only belongs to localized mode.

Supporting Note 5: Redox switching of organic VIS-NIR CLRs

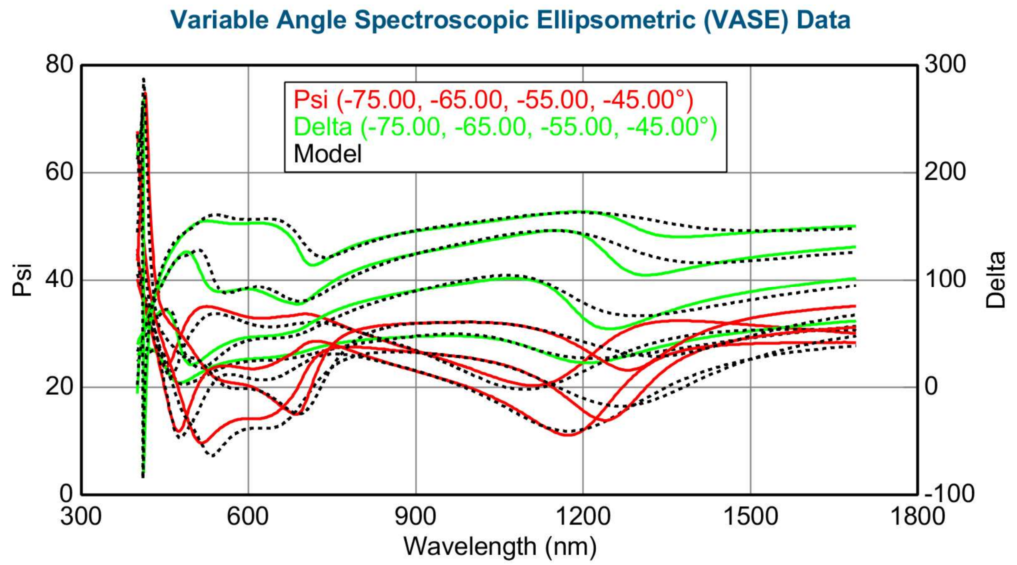

Figure S56. Spectroscopic ellipsometry data (ranging from 400 nm to 1690 nm) for the less reduced state of a PEDOT film (acid-treated PEDOT:ToS). The film thickness is about 200 nm. These raw data and the fitting were processed using the VASE software. The Psi (ψ, marked in the red line) and Delta (Δ, marked in the green line) were acquired at four angles (45°, 55°, 65°, and, 75°). The black dashed lines are the best fitting data by using the Drude-Lorents model. It is noted that although using B-spline model enables more accurate fitting with experimental spectroscopic ellipsometry, the almost perfect fitting also causes an overfitting problem, where the noise signals can also be fitting and form artificial refractive index[6, 7]. Thus, even with some deviations (but within reasonable scope), we still use Drude-Lorents model to fit the spectra (also including the following Figure S57-S59).

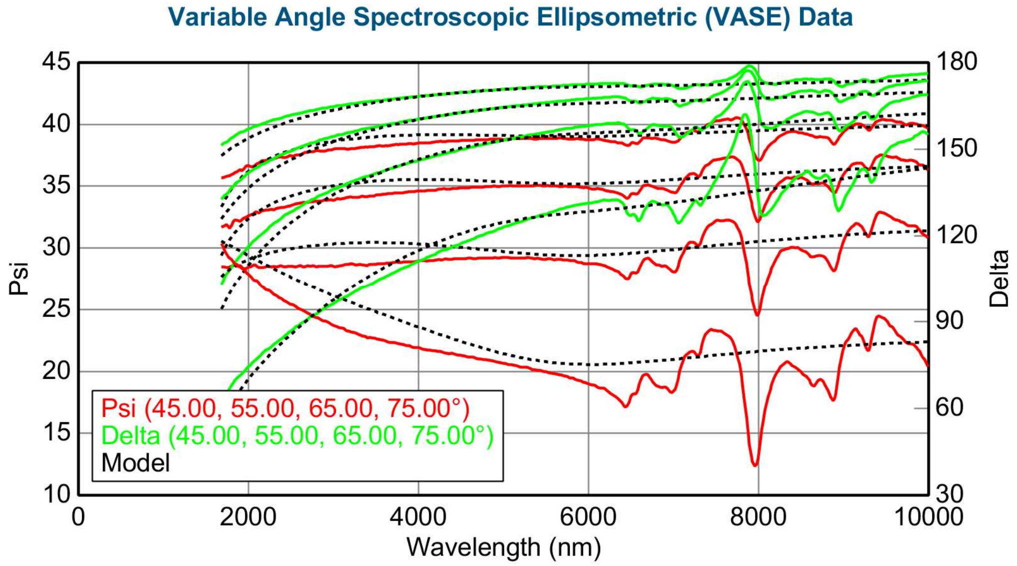

Figure S57. Spectroscopic ellipsometry data (ranging from 1690 nm to 10000 nm) for the less reduced state of a PEDOT film (acid-treated PEDOT:ToS). The film thickness is about 200 nm. These raw data and the fitting were processed using the VASE software. The Psi (ψ, marked in the red line) and Delta (Δ, marked in the green line) were acquired at four angles (45°, 55°, 65°, and, 75°). The black dashed lines are the best fitting data by using the Drude-Lorents model.

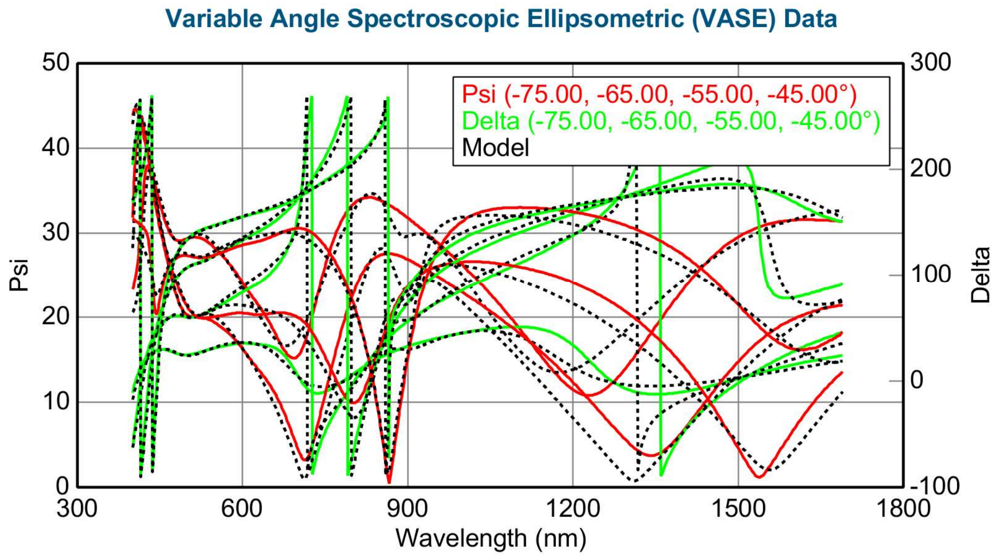

Figure S58. Spectroscopic ellipsometry data (ranging from 400 nm to 1690 nm) for the more reduced state of a PEDOT film (acid-treated PEDOT:ToS). The film thickness is about 200 nm. These raw data and the fitting were processed using the VASE software. The Psi (ψ, marked in the red line) and Delta (Δ, marked in the green line) were acquired at four angles (45°, 55°, 65°, and, 75°). The black dashed lines are the best fitting data by using the Drude-Lorents model.

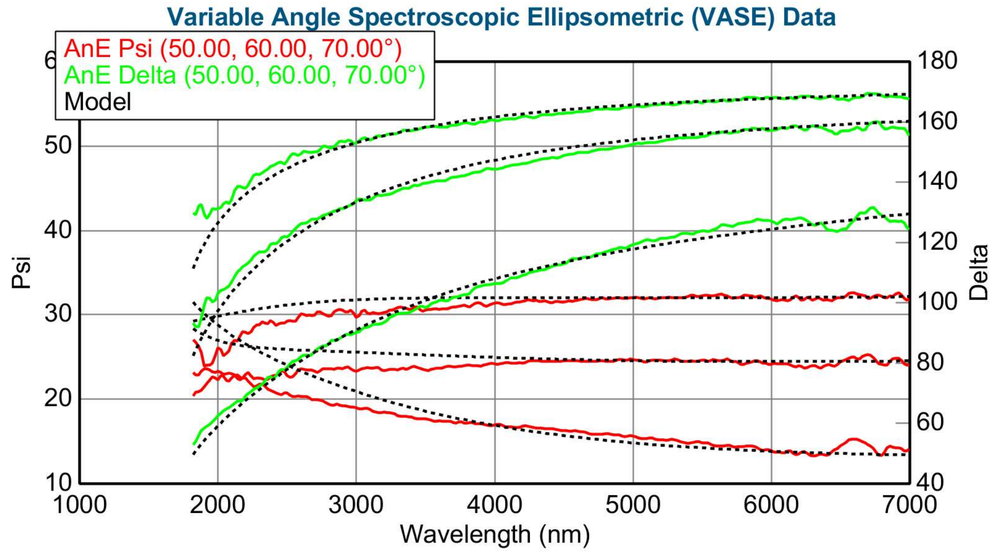

Figure S59. Spectroscopic ellipsometry data (ranging from 1690 nm to 10000 nm) for the more reduced state of a PEDOT film (acid-treated PEDOT:ToS). The film thickness is about 200 nm. These raw data and the fitting were processed using the VASE software. The Psi (ψ, marked in the red line) and Delta (Δ, marked in the green line) were acquired at four angles (45°, 55°, 65°, and, 75°). The black dashed lines are the best fitting data by using the Drude-Lorents model.

Here we use the Drude-Lorentz equation[3, 8]:

$$\varepsilon(\omega) = \varepsilon_{\infty} - \frac{\omega_{\mathrm{p}}^2}{\omega^2 + i\omega\gamma} - \sum_j \frac{A_{\mathrm{j}}}{\omega^2 - \omega_{\mathrm{j}}^2 + i\omega\gamma_{\mathrm{j}}} \quad \text{(S51)}$$

to fit the permittivities of less and more reduced states of PEDOT film, where ω is the angular frequency, ε_{∞} is the permittivity at infinitely high frequency (beyond the measurement range), γ is the momentum-averaged broadening (related to the damping relaxation), i is the imaginary

unit, ω_p is the plasma frequency. A_j, ω_j and γ_j are amplitude, resonance angular frequency, and broadening (or damping relaxation part) of the j-th Lorentz oscillator, respectively. The Drude part has a similar mathematic type of Lorentz oscillator, although with $\omega_j = 0$.

Table S1: Oscillators for less reduced state along the in-plane direction

$\varepsilon_\infty = 2.800$			
Oscillator No. (j th)	Frequency ω_j (eV)	Broadening γ_j (eV)	Amplitude A_j (eV^2)
Drude	0	0.531	2.969
1	2.008	0.911	2.441
2	1.320	0.350	0.544
3	7.472	4.000	9.349

Table S2: Oscillators for less reduced state along the out-of-plane direction

$\varepsilon_\infty = 0.946$			
Oscillator No. (j th)	Frequency ω_j (eV)	Broadening γ_j (eV)	Amplitude A_j (eV^2)
Drude	0	0.159	0.192
1	2.484	0.3108	2.373
2	4.601	0.159	62.883
3	3.366	1.000	1.011

Table S3: Oscillators for more reduced state along the in-plane direction

$\varepsilon_\infty = 2.249$			
Oscillator No. (j th)	Frequency ω_j (eV)	Broadening γ_j (eV)	Amplitude A_j (eV^2)
Drude	0	0.516	1.451
1	2.055	0.680	3.370
2	7.278	1.339	31.911

Table S4: Oscillators for more reduced state along the out-of-plane direction

$\varepsilon_\infty = 2.276$			
Oscillator No. (j th)	Frequency ω_j (eV)	Broadening γ_j (eV)	Amplitude A_j (eV^2)
Drude	0	0.329	0.134
1	6.426	2.782	22.133
2	1.364	0.117	0.149

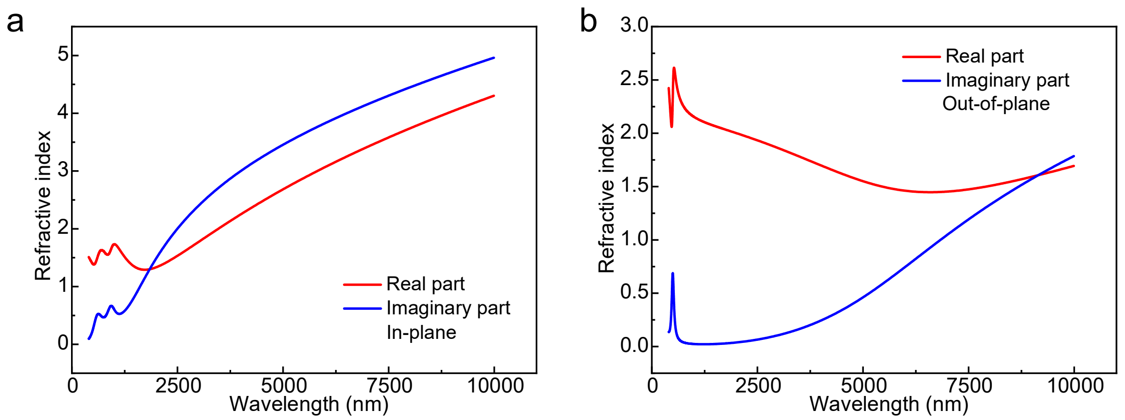

Figure S60. Refractive index of partially reduced state of PEDOT film. The PEDOT material was acid-treated PEDOT:ToS (thickness: ~ 200 nm). These data were obtained based on fitting spectroscopic ellipsometry through Drude-Lorentz model. (a) Along the in-plane direction. (b) Along the out-of-plane direction.

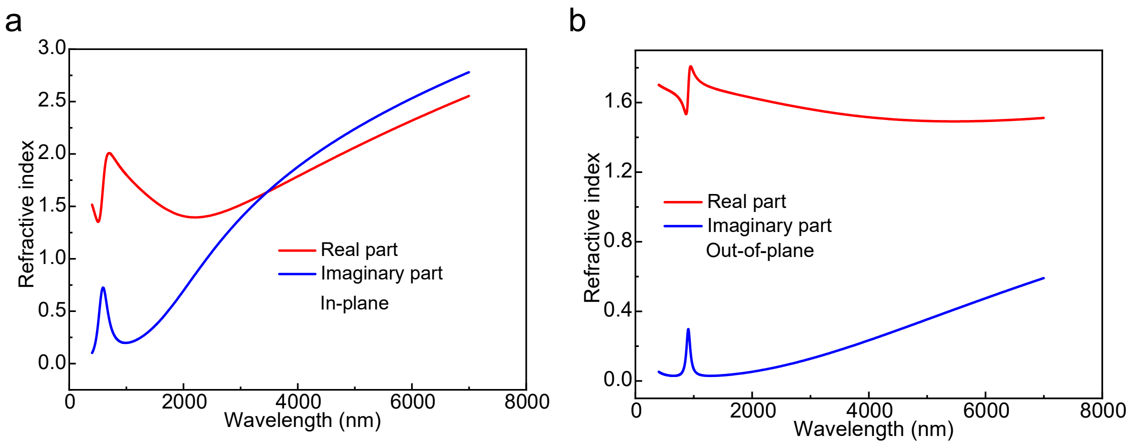

Figure S61. Refractive index of more reduced state of PEDOT film. The PEDOT material was acid-treated PEDOT:ToS (thickness: ~ 200 nm). These data were obtained based on fitting spectroscopic ellipsometry through Drude-Lorentz model. (a) Along the in-plane direction. (b) Along the out-of-plane direction.

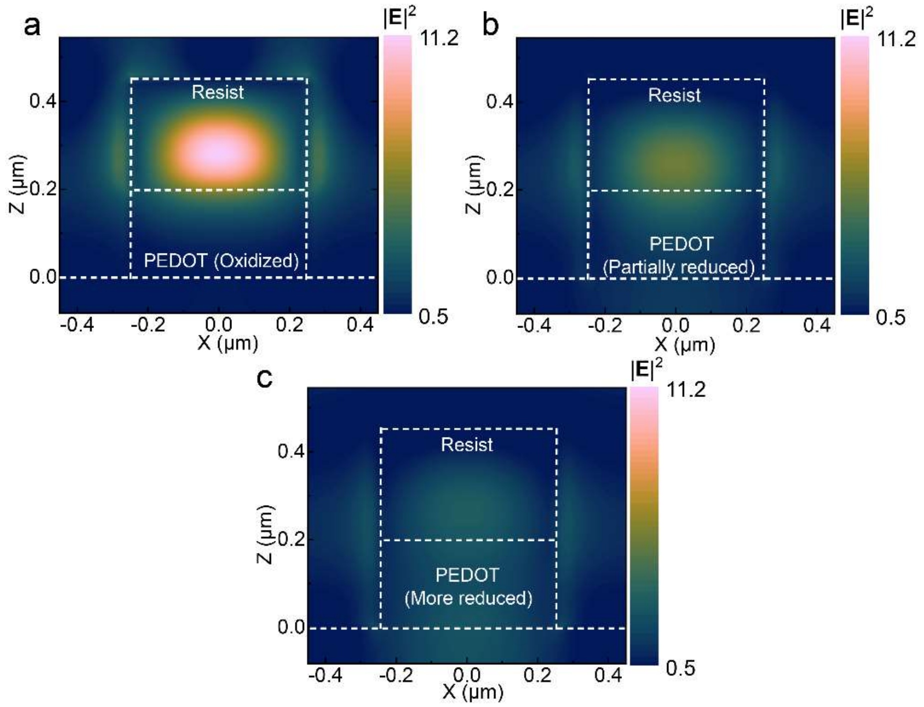

Figure S62. The electrical field distributions of Die-PEDOT-Nano in the hexagonal arrays (*r* = 0.9 μm), including the oxidized (a), partially reduced (b), and more reduced states (c). The diameter of complex nanoantenna unit is 0.48 μm. The scale bar of mapping is $|\mathbf{E}|^2$.

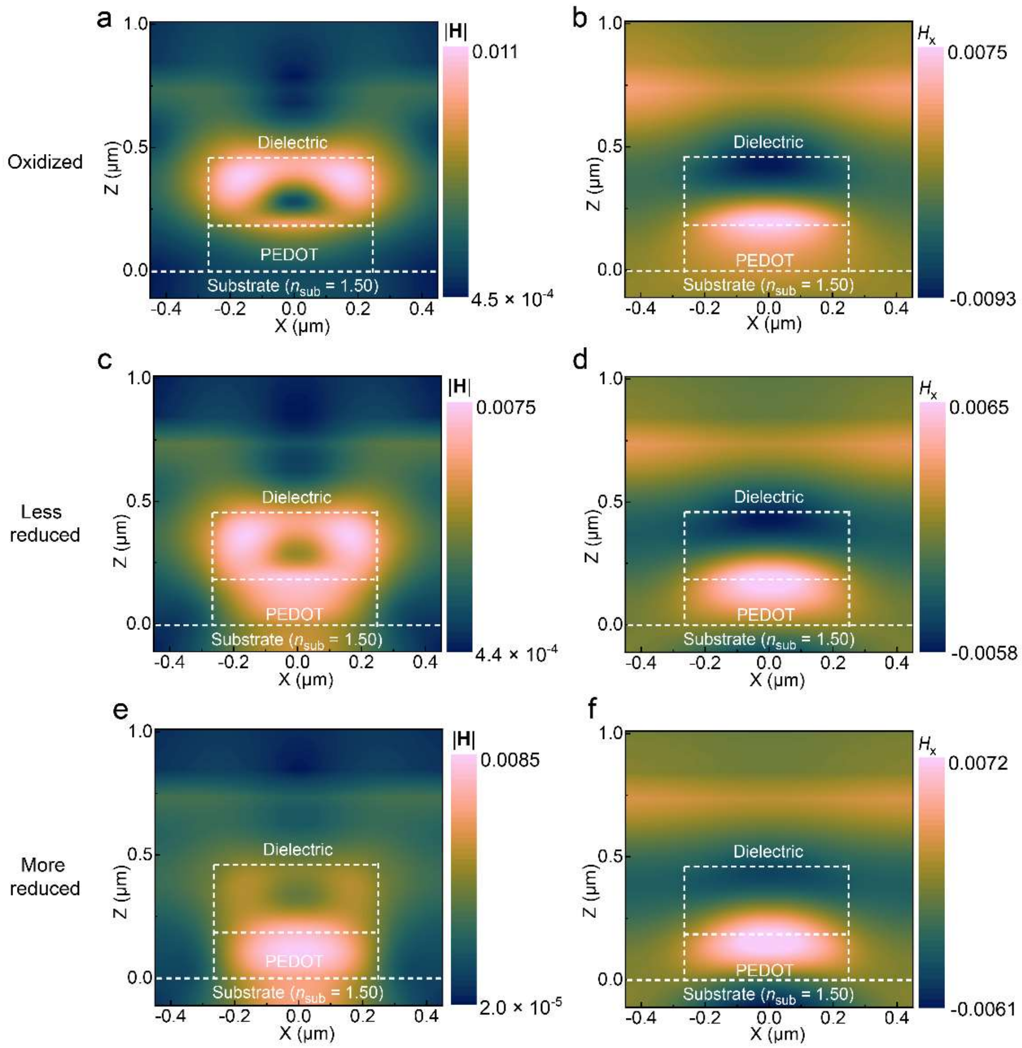

Figure S63. Magnetic near-field distribution of Die-PEDOT-Nano arrays in different redox states around at $\lambda_r \approx 815$ nm. (a) (b) Magnitude of magnetic field (|**H**|) and magnetic field along *X*-axis (H_x) in oxidized state, respectively. (c) (d) Magnitude of magnetic field (|**H**|) and magnetic field along *X*-axis (H_x) in less reduced state, respectively. (e) (f) Magnitude of magnetic field (|**H**|) and magnetic field along *X*-axis (H_x) in more reduced state, respectively. The periodic distance of these arrays is $r = 0.9$ μm. The directions of wave vector, electric vector and magnetic vector of incident light are along *Z*-axis, *Y*-axis and *X*-axis, respectively. These near-field distributions are based on the TE mode. When performing reduction reactions, the magnetic field is also expanded into PEDOT nanoantennas, which also confirms the elimination of light confinement.

*Mathematic equations about Fano resonance

Here the extinction spectra of Die-PEDOT-Nano arrays were fitted by Fano formula[9, 10]:

$$\sigma_{\mathrm{ext}} = D^2 \frac{(q+\Omega)^2}{1+\Omega^2} \tag{S52}$$

, where σ_{ext} is the normalized extinction, $\Omega = 2(E - E_0)/\Gamma$ is related to the resonance energy (E_0) and linewidth (Γ), q is the Fano parameter or asymmetry factor. Considering $q = \cot\delta$ and $D = 2\sin\delta$, where δ is the phase shift to the continuum, Equation (S52) is converted into:

$$\sigma_{\mathrm{ext}} = \frac{4(\cos\delta + \Omega\sin\delta)^2}{1+\Omega^2} \tag{S53}$$

. For the resonance from discrete state, the resonance frequency (with the energy unit: eV) is ω_d, and the corresponding linewidth is γ_{Fano} (with the energy unit: eV). The resonance frequency (with the energy unit: eV) of the continuum is ω_c. The δ is evaluated by the formula:

$$e^{i\delta} = \frac{1}{\omega_c - \omega_d + i\gamma_{Fano}} \tag{S54}$$

If fixing ω_c and ω_d, the increase of γ_{Fano} (by enhancing damping relaxation) enlarges the phase shift δ toward the continuum, which enhances the influence of a continuum on the extinction spectra.

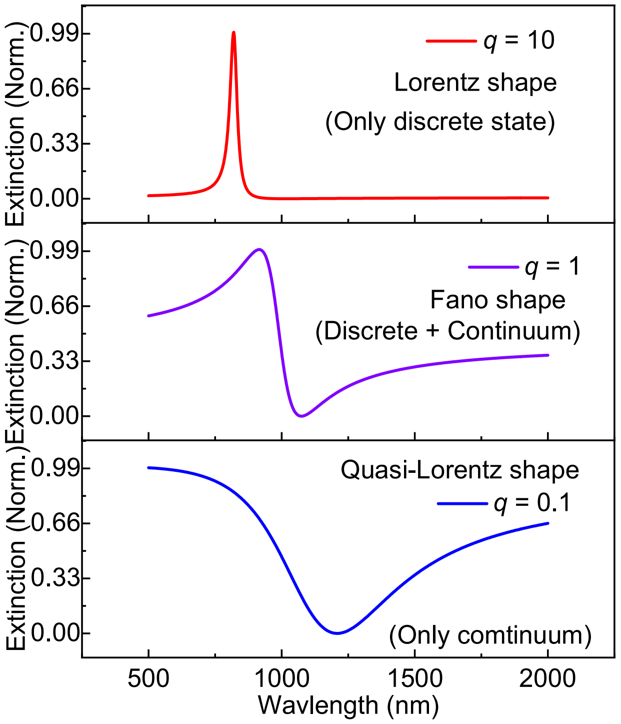

Figure S64. Different *q* values in Fano resonance. For $q = 10$, we use $\gamma_{\mathrm{Fano}} = 0.05$, $E_0 = 1.50$ eV and $\Gamma = 0.06$ eV to calculate the normalized extinction spectra in Lorentz shape (without coupling to the continuum). For $q = 1$, we use $\gamma_{\mathrm{Fano}} = 0.5$, $E_0 = 1.25$ eV and $\Gamma = 0.25$ eV to calculate the normalized extinction spectra in Fano shape (coupling discrete state to the continuum). For $q = 0.1$, we use $\gamma_{\mathrm{Fano}} = 5$, $E_0 = 1.5$ eV and $\Gamma = 0.5$ eV to calculate the normalized extinction spectra in Quasi-Lorentz shape (without coupling to discrete state).

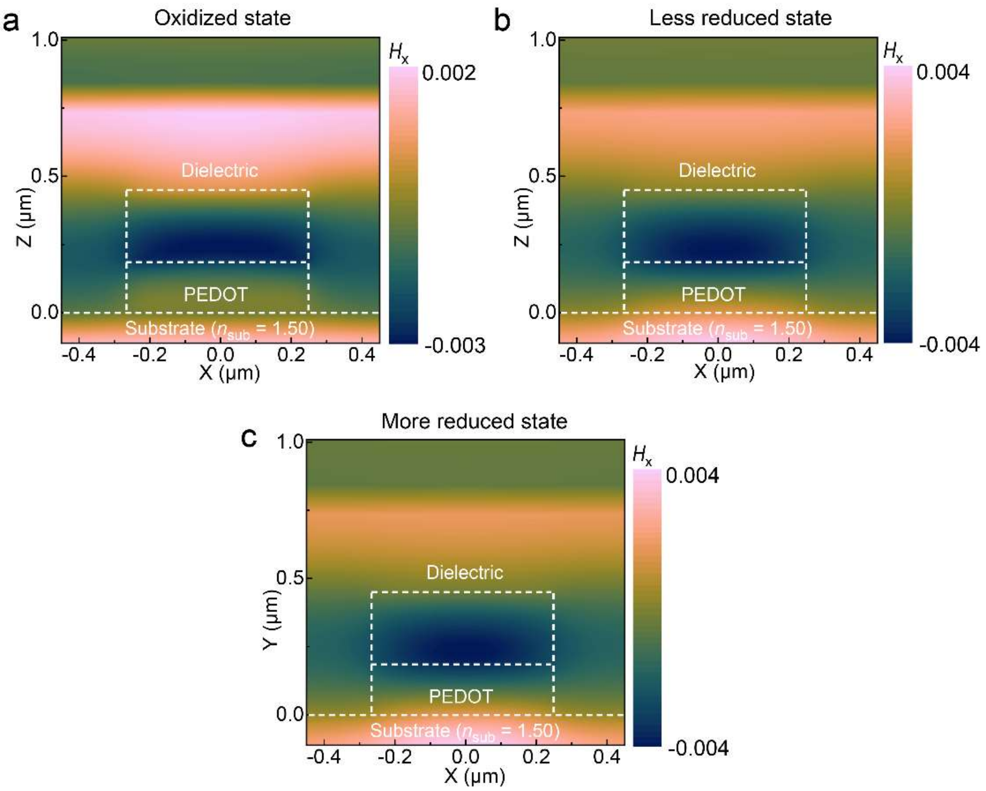

Figure S65. Magnetic near-field distribution of Die-PEDOT-Nano arrays in different redox states around at 1200 nm. (a) Magnetic field along *X*-axis (H_x) in oxidized state. (b) Magnetic field along X-axis (H_x) in less reduced state. (c) Magnetic field along *X*-axis (H_x) in more reduced state. The hybridized nanoantenna structures and periodic distances of hexagonal arrays are the same as those in Figure S62. The directions of wave vector, electric vector and magnetic vector of incident light are along *Z*-axis, *Y*-axis and *X*-axis, respectively. These near-field distributions are based on the TE mode. We observed that there was only an approximately planar wavefront when the magnetic field passes through the nanoantennas, indicating the negligible role of nanoantennas in scattering. Thus, these nearfield distributions are approximate to the feature of Fano invisibility[9, 10], consistent with the minimum point in the extinction spectra.

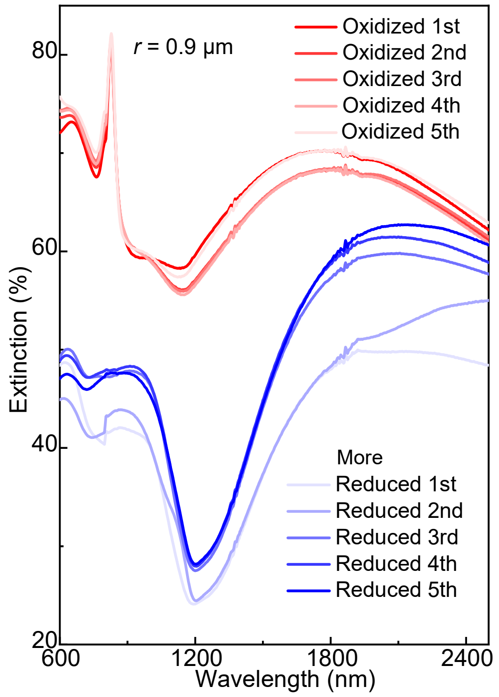

Figure S66. Measured extinction spectra of Die-PEDOT-Nano arrays (*d* = 0.5 μm and *r* = 0.9 μm) during redox cycles in 5 times, in the wavelength ranges of 600-2500 nm.

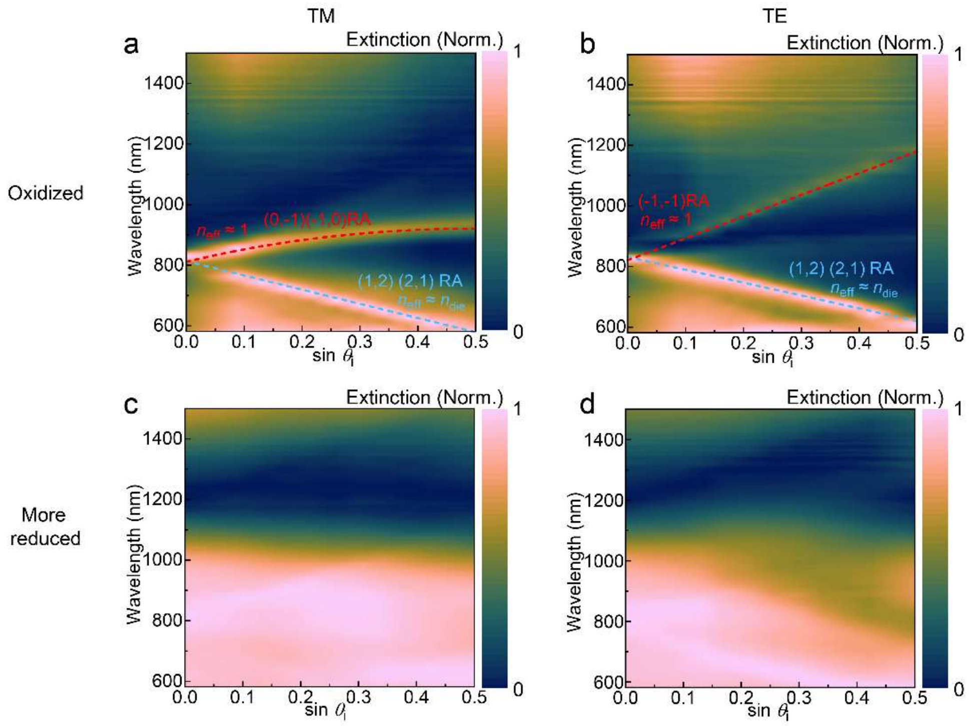

Figure S67. Angle-dependent extinction spectra of the hexagonal Die-PEDOT-Nano array (*r* = 0.9 μm) after 5 times of redox cycles. (a) (b) TM and TE modes along the Γ−M direction, respectively, when the PEDOT layer was in oxidized state. (c) (d) TM and TE modes along the Γ−M direction, respectively, when the PEDOT layer was in more reduced state. The diameter of the hybridized nanoantenna unit is 0.48 μm.

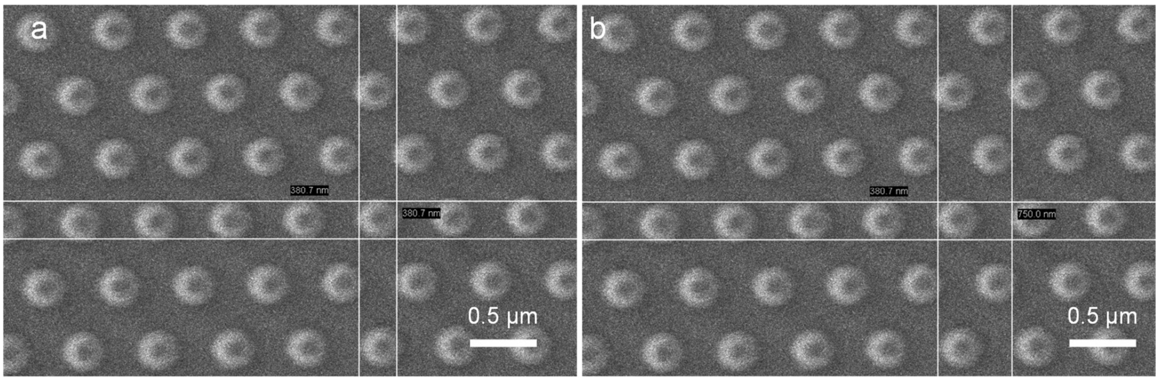

Figure S68. SEM images of Die-EDOT-Nano arrays prepared through electron beam lithography (hexagonal shapes, *r* = 0.75 μm**).** The PEDOT nanoantenna has a diameter *d* = 0.38 μm.

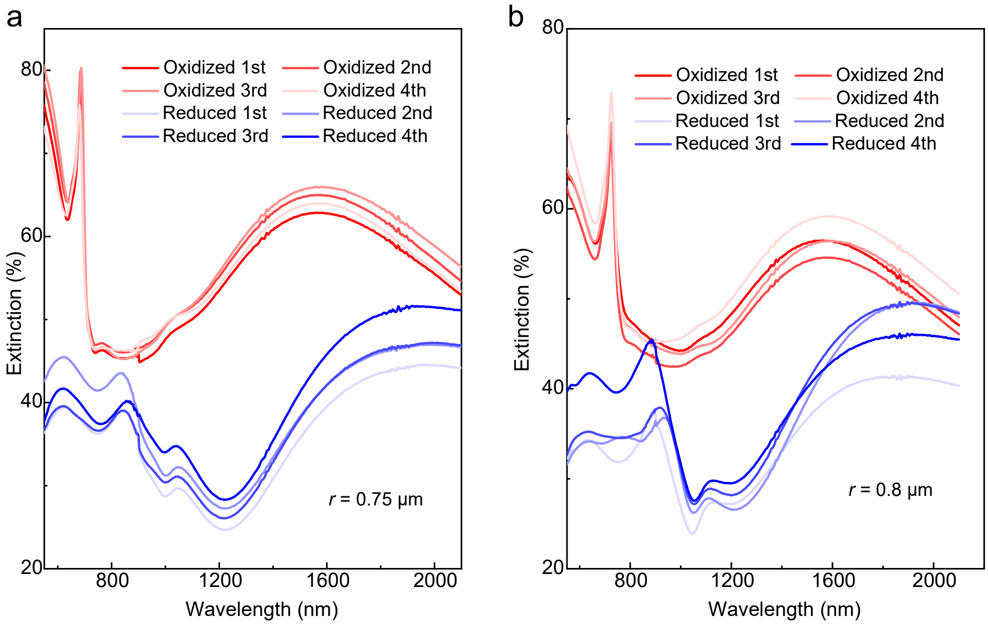

Figure S69. Dynamic switching of Visible nonlocal Mie resonances in 5 times of redox cycles. (a) *r* = 0.75 μm (*d* = 0.38 μm); (b) *r* = 0.8 μm (*d* = 0.41 μm). The SEM images are shown in Figure S60 and S25.

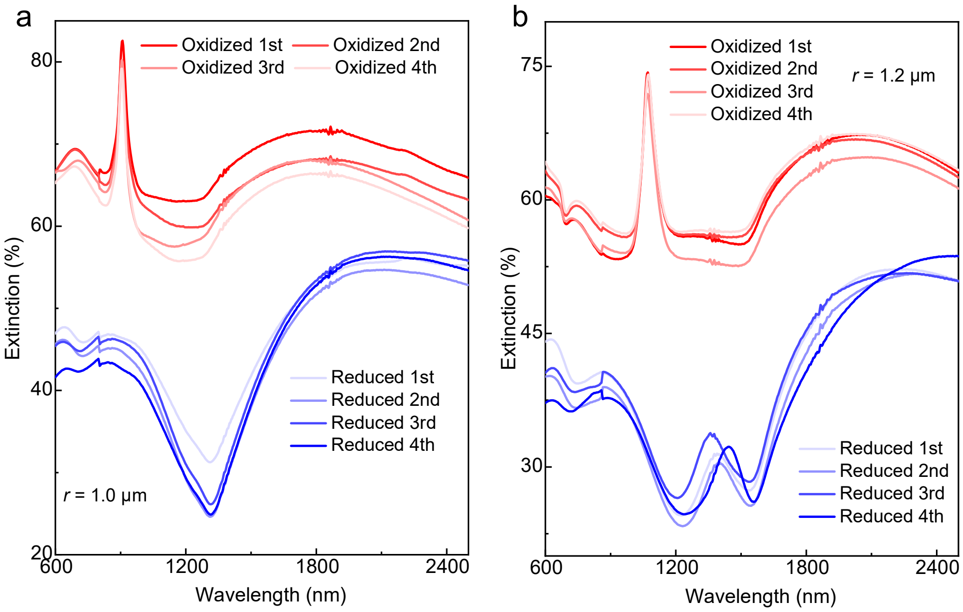

Figure S70. Dynamic switching of NIR nonlocal Mie resonances in 5 times of redox cycles. (a) r = 1.0 μm (d = 0.53 μm); (b) r = 1.2 μm (d = 0.61 μm). The SEM images are shown in Figure S25 and S26.

References:

1. Castellanos GW, Bai P, Gómez Rivas J. Lattice resonances in dielectric metasurfaces. *J. Appl. Phys.* **125**, 213105 (2019).
2. Lin D*, et al.* Switchable narrow nonlocal conducting polymer plasmonics. *Nat. Commun.* **16**, 4484 (2025).
3. Chen S*, et al.* Conductive polymer nanoantennas for dynamic organic plasmonics. *Nat. Nanotechnol.* **15**, 35-40 (2020).
4. Kazi S*, et al.* n-Type redox-tuneable conducting polymer optical nanoantennas. *J. Mater. Chem. C* **12**, 17469-17474 (2024).
5. Guo R, Hakala TK, Törmä P. Geometry dependence of surface lattice resonances in plasmonic nanoparticle arrays. *Phys. Rev. B* **95**, 155423 (2017).
6. Likhachev DV. Selecting the right number of knots for B-spline parameterization of the dielectric functions in spectroscopic ellipsometry data analysis. *Thin Solid Films* **636**, 519-526 (2017).
7. Likhachev DV. Certain topics in ellipsometric data modeling with splines: a review of recent developments. *Adv. Opt. Technol.* **11**, 93-115 (2022).
8. Chen S*, et al.* On the anomalous optical conductivity dispersion of electrically conducting polymers: ultra-wide spectral range ellipsometry combined with a Drude–Lorentz model. *J. Mater. Chem. C* **7**, 4350-4362 (2019).
9. Rybin MV*, et al.* Switchable invisibility of dielectric resonators. *Phys. Rev. B* **95**, 165119 (2017).
10. Limonov MF, Rybin MV, Poddubny AN, Kivshar YS. Fano resonances in photonics. *Nat. Photonics* **11**, 543-554 (2017).
